# Supplementary material for: Assessing Human Genome-wide Variation in the Massim Region of Papua New Guinea and Implications for the Kula Trading Tradition
Source: Mol Biol Evol. 2022 Aug 3;39(8):msac165. doi: 10.1093/molbev/msac165 (PMC9372566; doi:10.1093/molbev/msac165)
Supplement: msac165_Supplementary_Data [file msac165_supplementary_data.pdf]

Supplementary Materials for

Assessing human genome-wide variation in the Massim region of Papua New Guinea and implications for the Kula trading tradition

Dang Liu, Benjamin M. Peter, Wulf Schiefenhövel, Manfred Kayser* and Mark Stoneking*

***Corresponding authors:** Mark Stoneking, stoneking@eva.mpg.de; Manfred Kayser, m.kayser@erasmusmc.nl

This PDF file includes:

Figures S1 to S22
Tables S1 to S2

Supplementary Figures

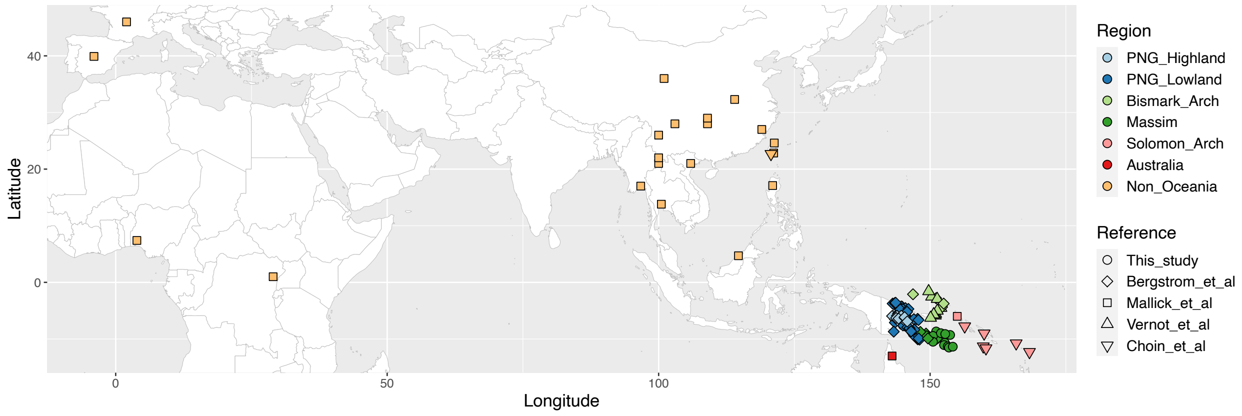

Fig. S1. Map showing the location of the studied groups.

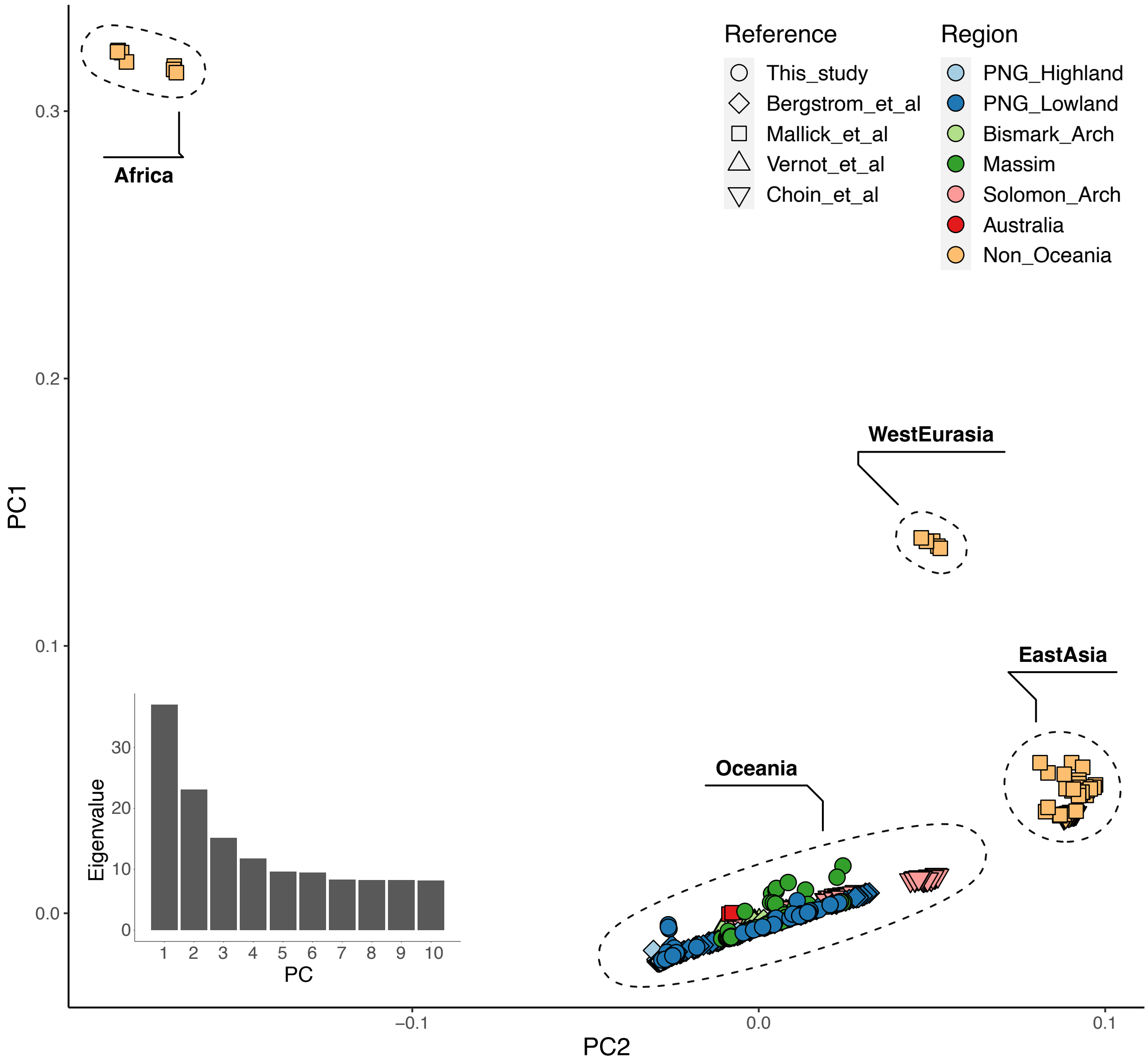

Fig. S2. Principal component analyses (PCA) plot of PC1 vs. PC2 for all individuals in the dataset. Symbol colors indicate region and shapes indicate the corresponding publication. The eigenvalues from PC1 to PC10 are shown on the bottom left.

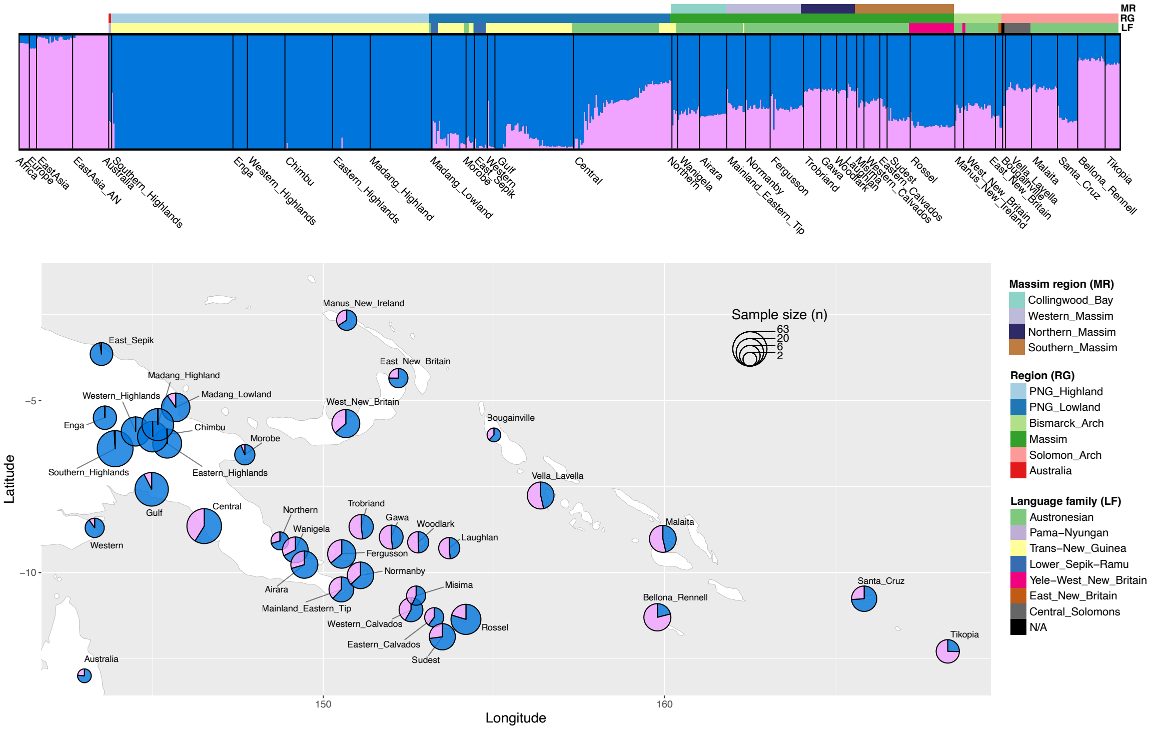

Fig. S3. K=2 ADMIXTURE analyses focusing on Oceanian structure. ADMIXTURE results for K=2 at the top, plotted on a map at the bottom. Color bars on the top indicate the region (RG), language family (LF) for the Oceanian groups and Massim region (MR) for the Massim groups. The size of the circle for each group is proportional to the sample size.

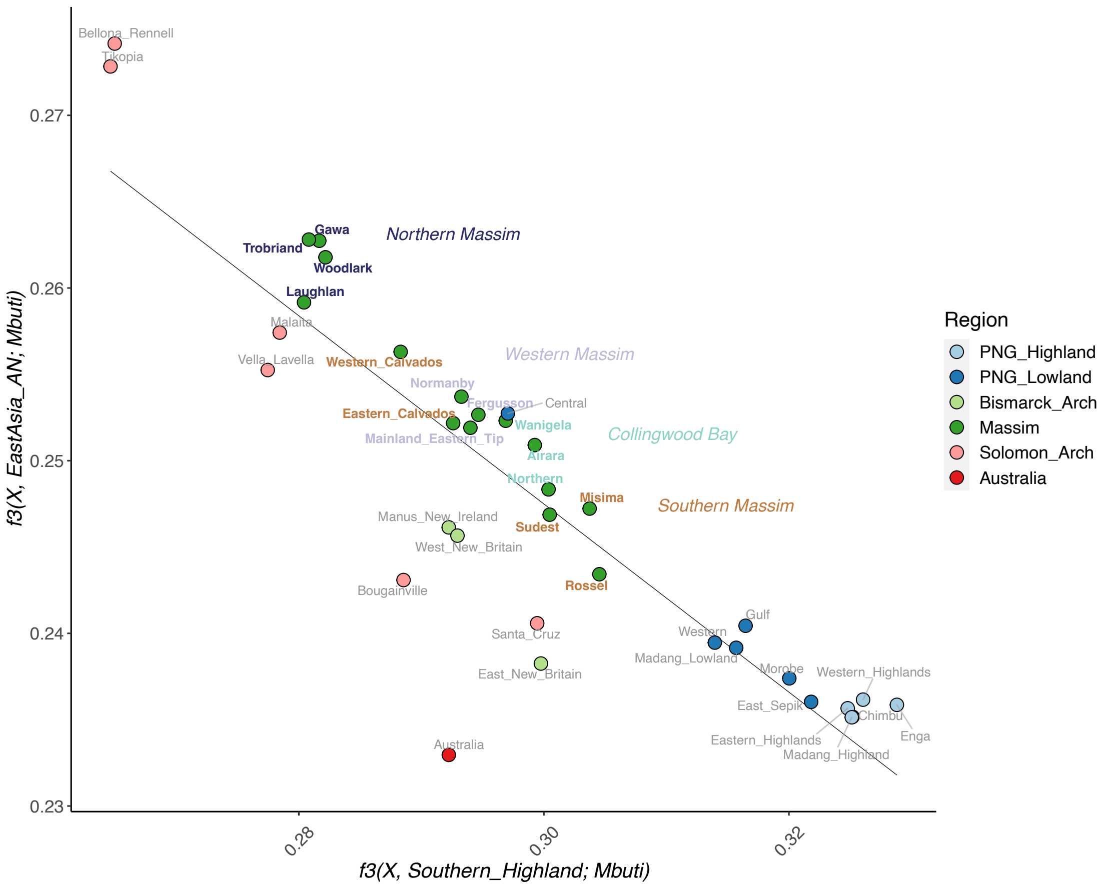

Fig. S4. F3 statistics measuring shared genetic drift between Oceanians and East Asian Austronesians vs. between Oceanians and PNG highlanders. The value of f3(X, Southern province highlanders; Mbuti) is on the x-axis, and the value of f3(X, East Asian Austronesians; Mbuti) is on the y-axis. X denotes the Oceanian groups, colored according to region, with Massim regions further highlighted. The linear regression line was computed using all values shown on the plot.

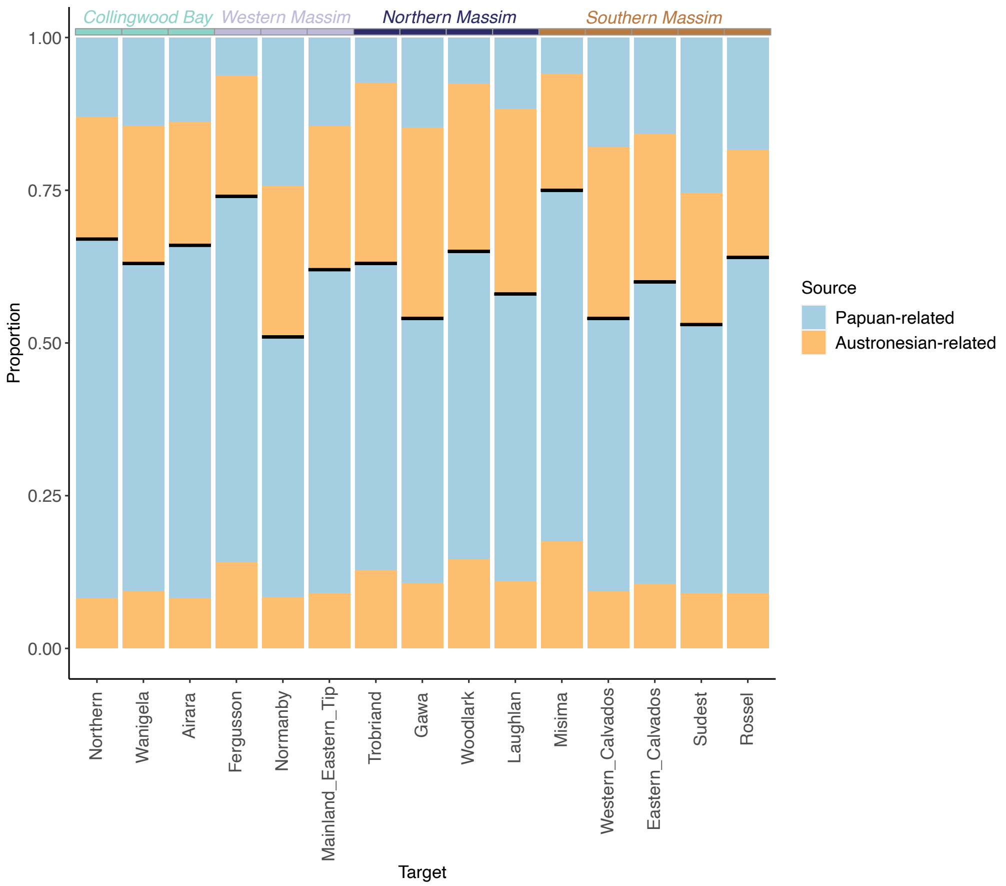

Fig. S5. Admixture sources inferred by GLOBETROTTER. The middle horizontal black line in each bar separates the minor source (top) from the major source (bottom).

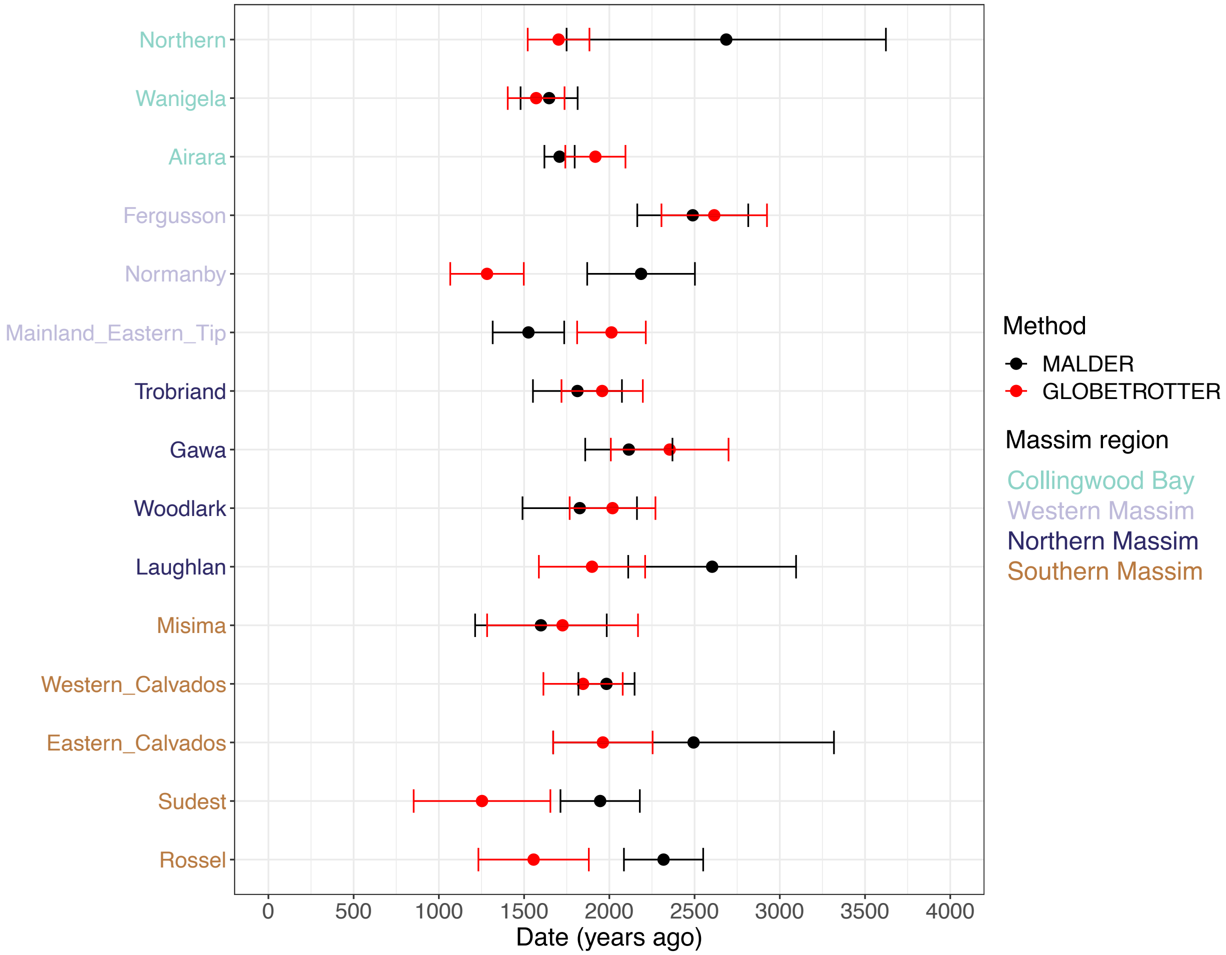

Fig. S6. Admixture dates inferred by GLOBETROTTER and MALDER. Massim group labels are colored according to Massim region. GLOBETROTTER results are in red while MALDER results are in black. MALDER was performed using East Asian Austronesians and Southern Highlanders as sources. Error bars indicate +/- one standard error.

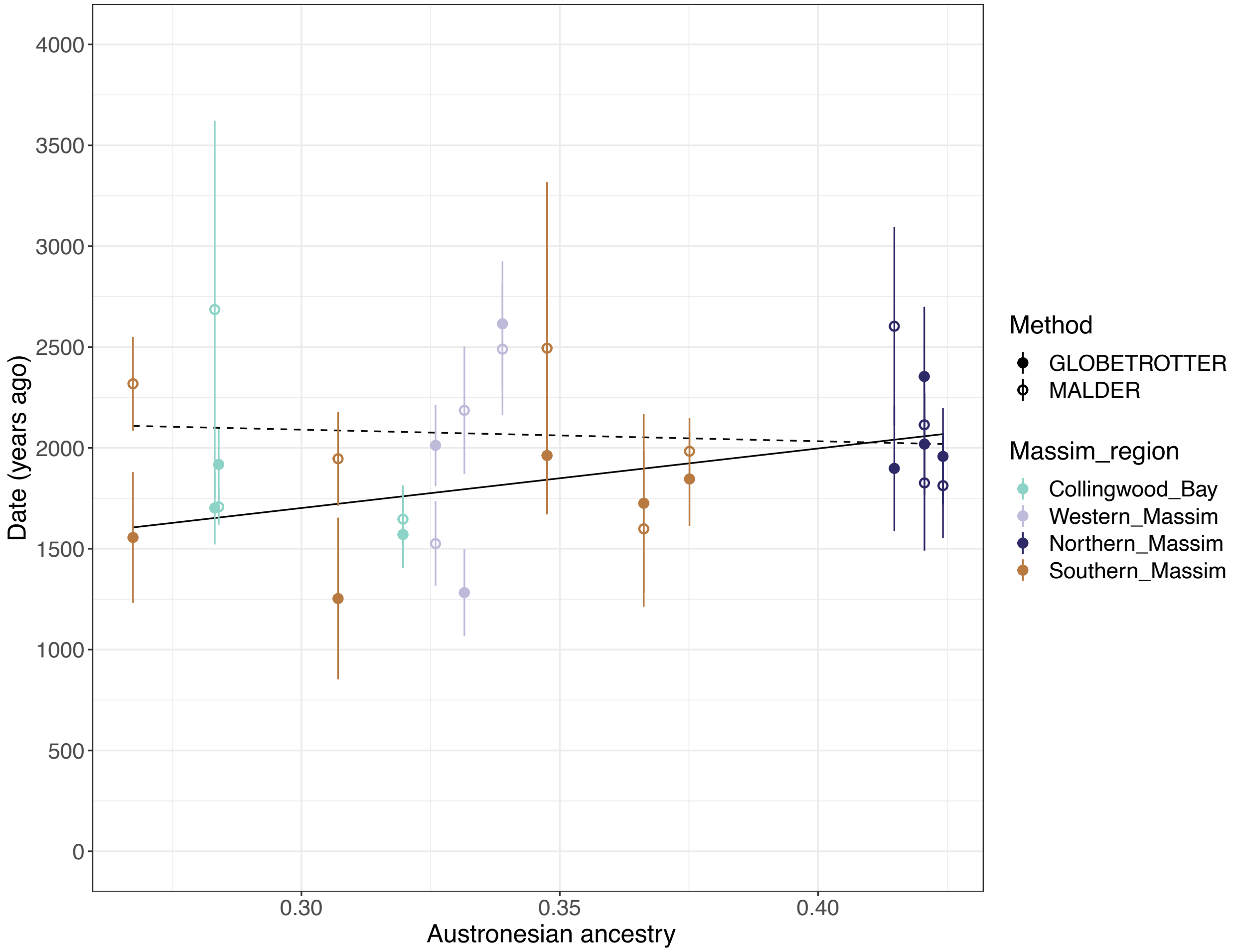

Fig. S7. Admixture dates vs. Austronesian ancestry for Massim groups. A solid or empty point indicates that the admixture date was inferred by GLOBETROTTER or MALDER, respectively; points are colored according to Massim region. Error bar indicates +/- one standard error of the inferred admixture dates. A solid or dashed regression line was calculated for the GLOBETROTTER or MALDER points, respectively. The linear regression results for GLOBETROTTER points: r^2=0.193 and p=0.102; and for MALDER points: r^2=0.006 and p=0.778.

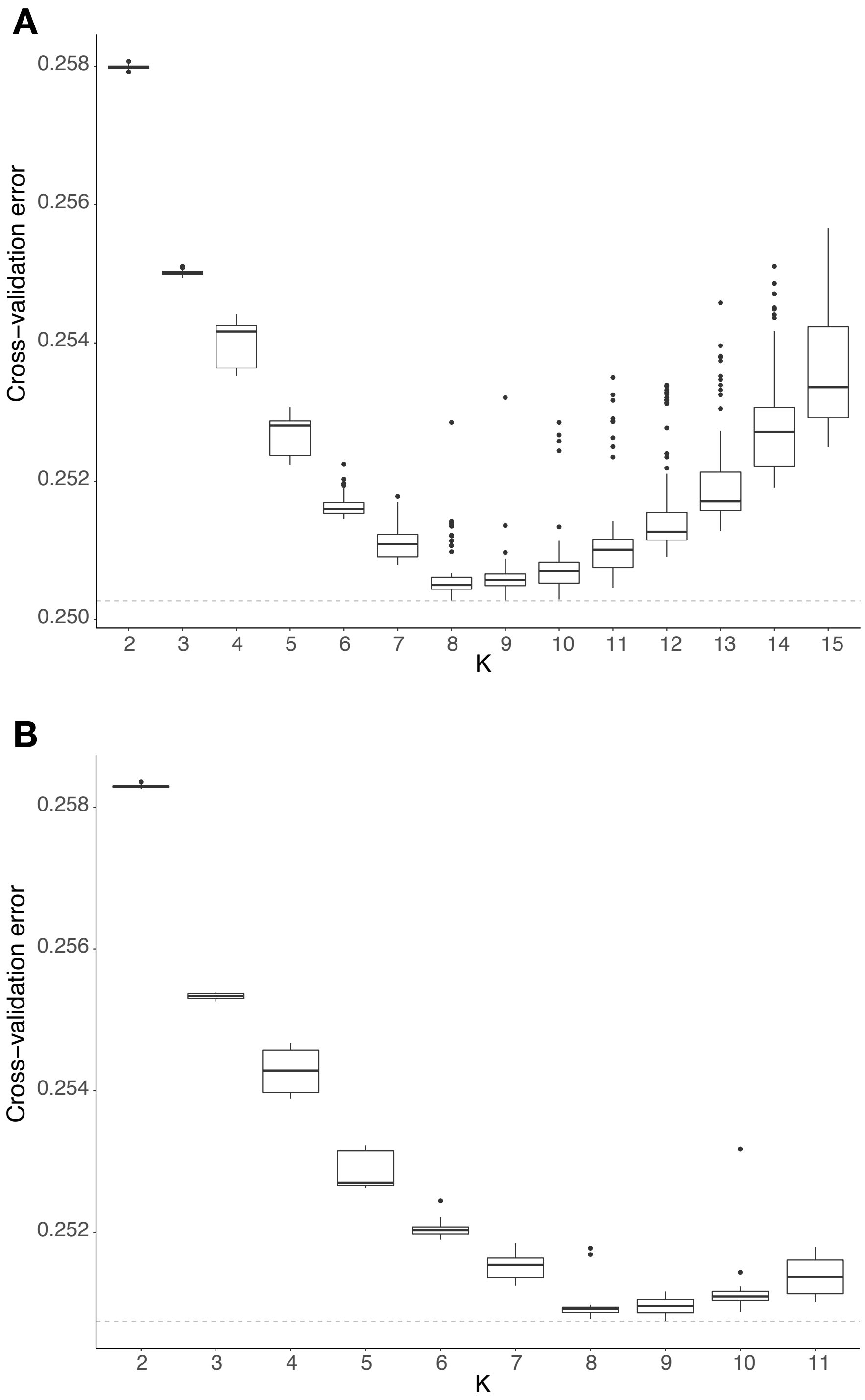

Fig. S8. Cross validation errors of ADMIXTURE runs. (A) for the entire dataset for K= 2 to K = 15, based on 100 runs for each K value. (B) with individuals with 2nd degree kinship excluded for K= 2 to K = 11, based on 20 runs for each K value. The lowest cross-validation error occurred at K = 8 for both datasets.

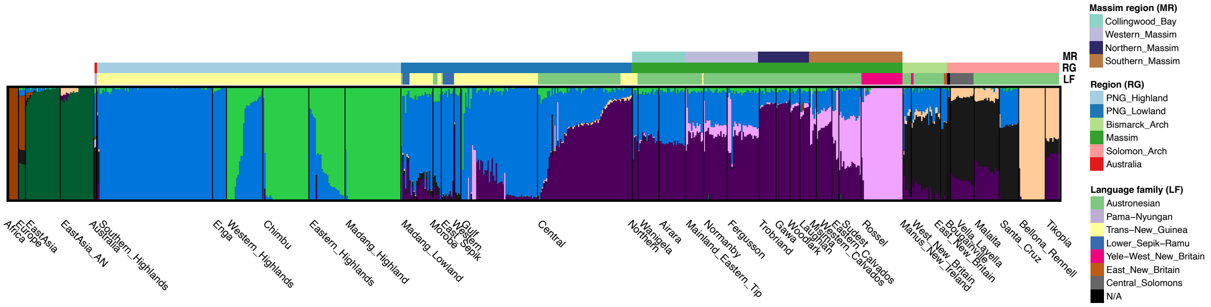

Fig. S9. K=8 ADMIXTURE analysis with 2nd degree kinship pairs excluded. The major mode for 20 runs is shown; the same overall ancestry components were observed as in Fig. 2 in all but two runs. For these two runs, a few individuals from the Massim received their own ancestry component instead of Bougainville. Color bars on the top indicate the region (RG), language family (LF) for the Oceanian groups and Massim region (MR) for the Massim groups.

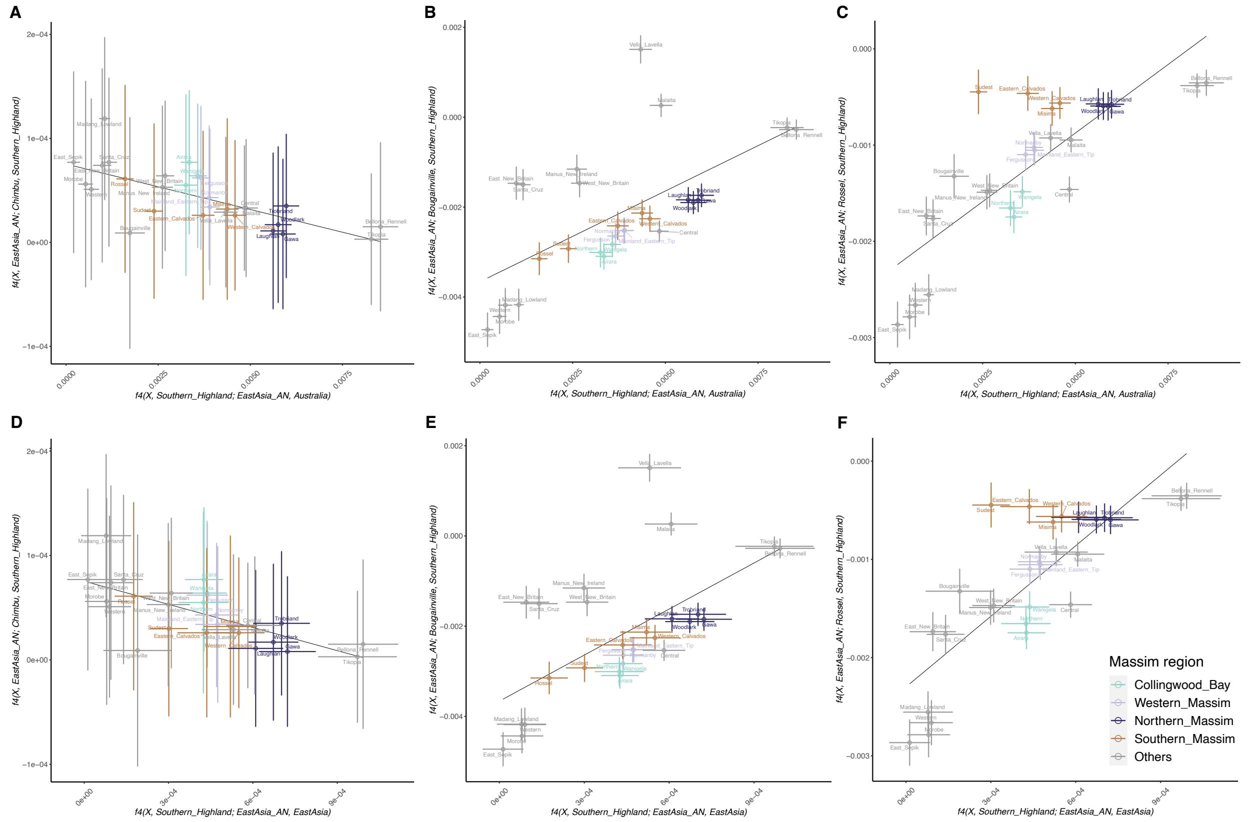

Fig. S10. F4 statistics measuring differential PNG ancestry affinities of Oceanian groups with respect to Austronesian ancestry affinity. The value of f4(Oceanian groups, Southern province highlanders; East Asian Austronesians; (A)-(C) Australians/ (D)-(F) non-Austronesian East Asians) is on the x-axis, and the value of f4(Oceania groups, East Asian Austronesians; (A) and (D) Chimbu province highlanders/ (B) and (E) Bougainville/ (C) and (F) Rossel islanders; Southern province highlanders) is on the y-axis. X denotes the Oceanian groups, colored according to Massim region. Error bars indicate +/- 3 SE. Linear regression lines were computed using all point values shown on the plot.

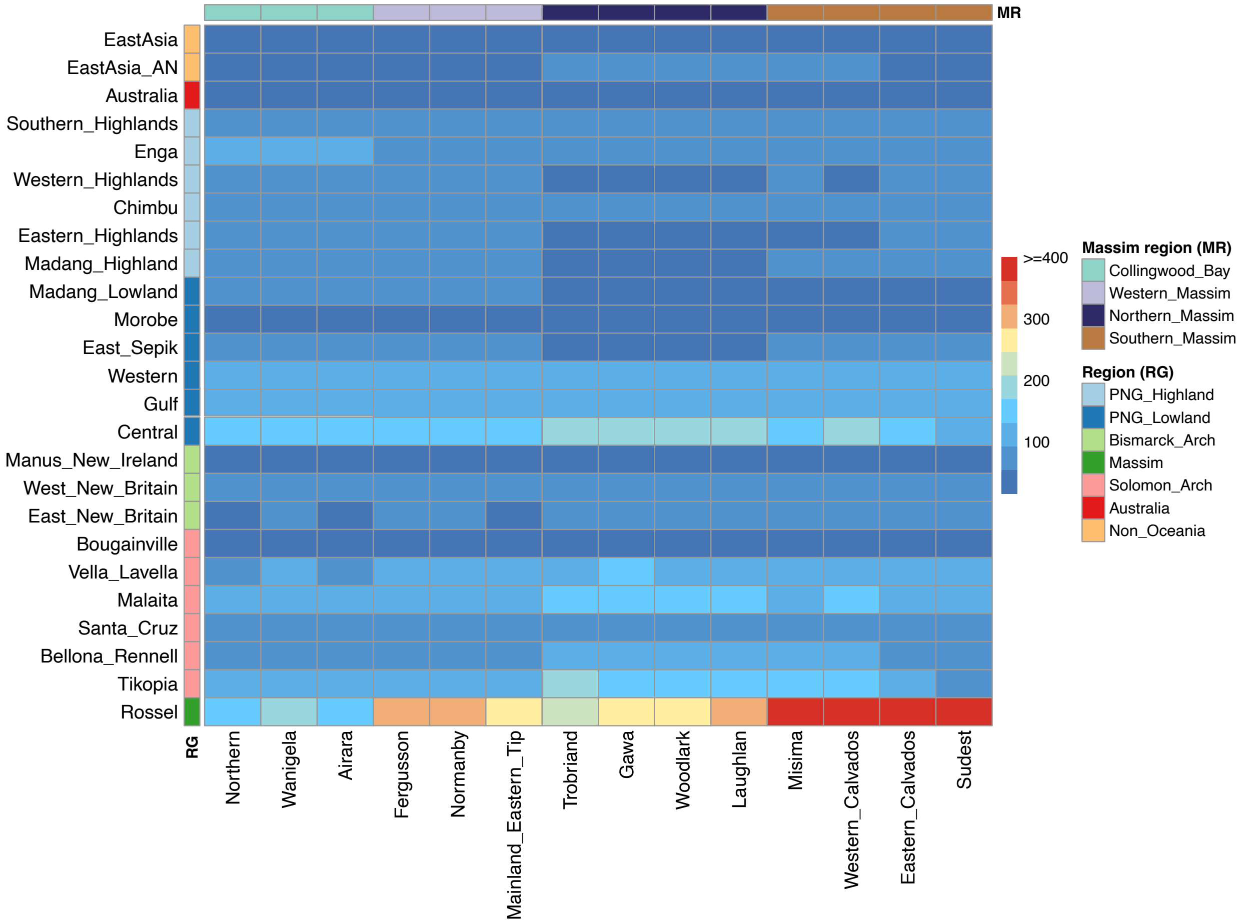

Fig. S11. ChromoPainter profile with Massim groups used only as recipients (except for Rossel, which was used as both a recipient and a donor). The heatmap is proportional to the total copied length (cM) of a recipient from a donor. Color bars at the left and top of the heatmap indicate the region (RG) and Massim subregion (MR), respectively.

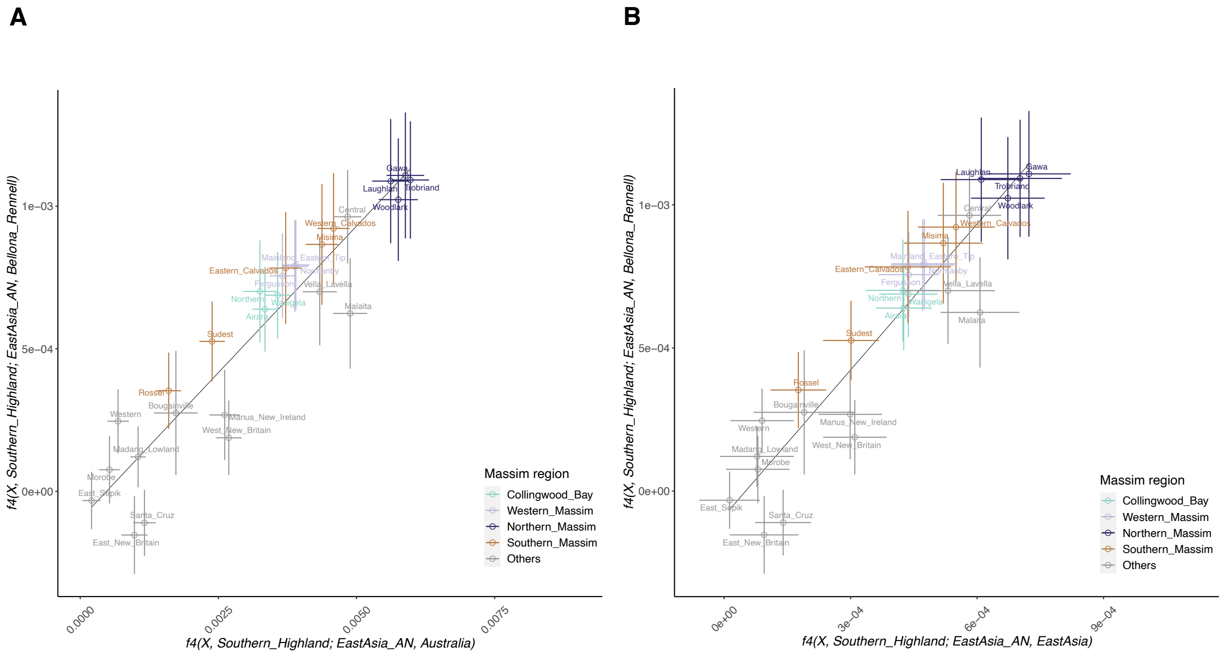

Fig. S12. F4 statistics measuring differential Austronesian ancestry affinities of Oceanian groups with respect to overall Austronesian ancestry affinity. The value of f4(Oceanian groups, Southern province highlanders; East Asian Austronesians; (A) Australians / (B) non-Austronesian East Asians) is on the x-axis, and the value of f4(Oceanian groups, Southern province highlanders; East Asian Austronesians; Bellona/Rennell) is on the y-axis. X denotes the Oceanian groups, colored according to Massim region. Error bars indicate +/- 3 SE. Linear regression lines were computed using all point values shown on the plot.

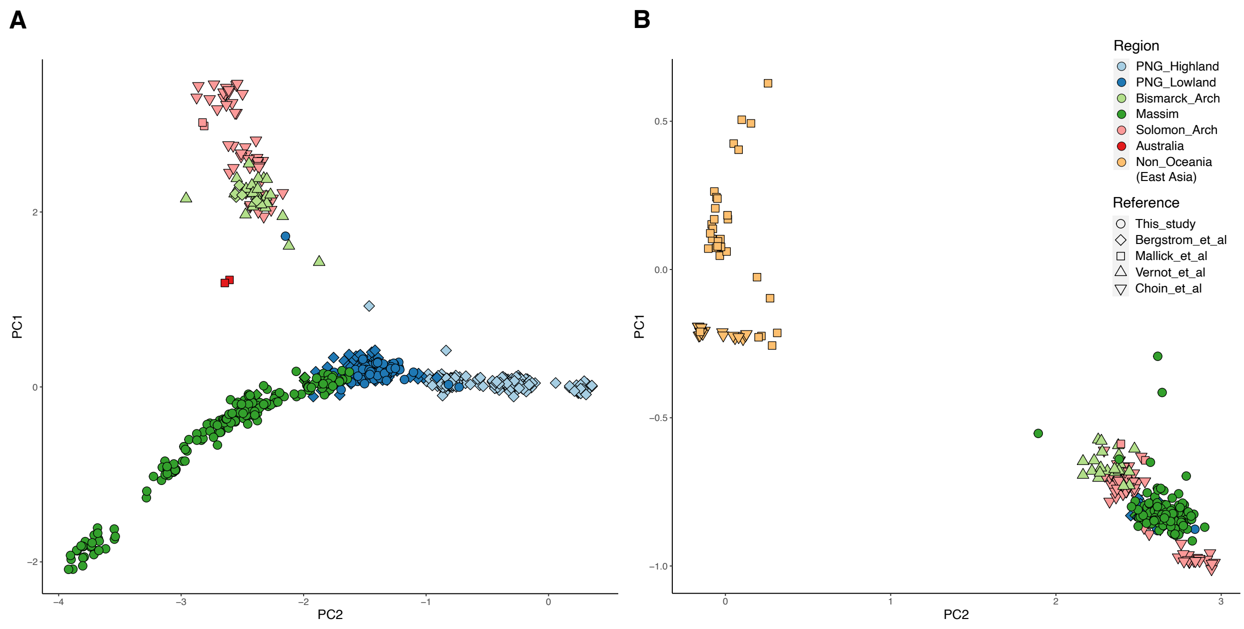

Fig. S13. Papuan/Austronesian ancestry-specific PCA of Oceanian and East Asian individuals. Plot of PC1 vs. PC2 for all individuals from Oceanian and East Asian groups and using only (A) Papuan or (B) Austronesian segments identified by RFMix, colored according to region.

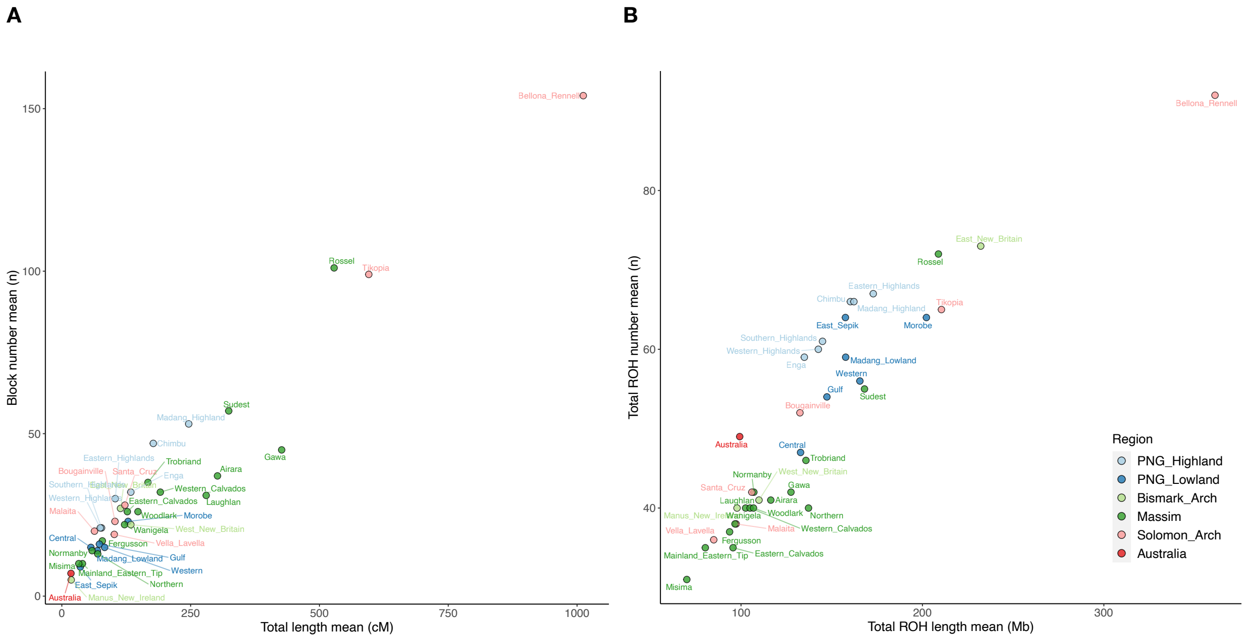

Fig. S14. IBD and ROH sharing within groups. (A) IBD and (B) ROH. The groups are colored according to region.

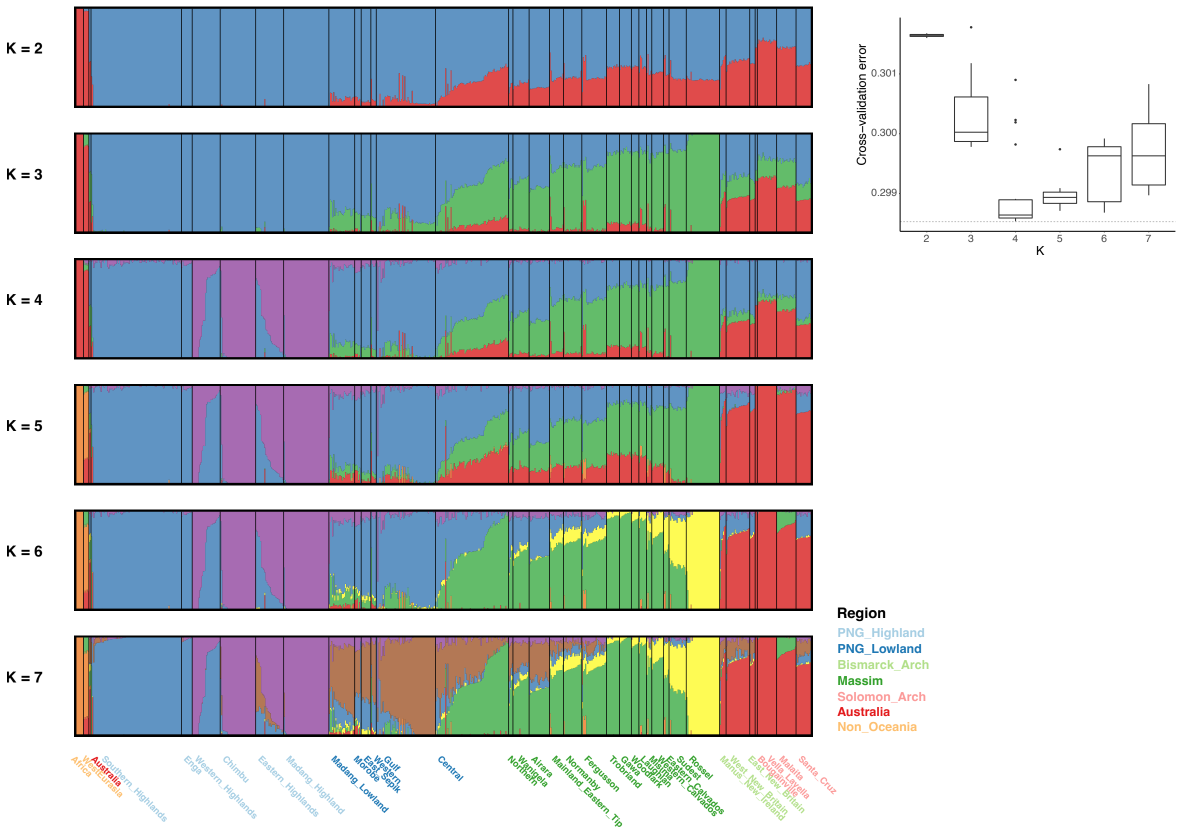

Fig. S15. Papuan ancestry-specific ADMIXTURE analysis. On the left are the results for K = 2 to K = 7 while on the right is the plot of the cross-validation errors, based on 20 runs for each K value. The groups are colored according to region.

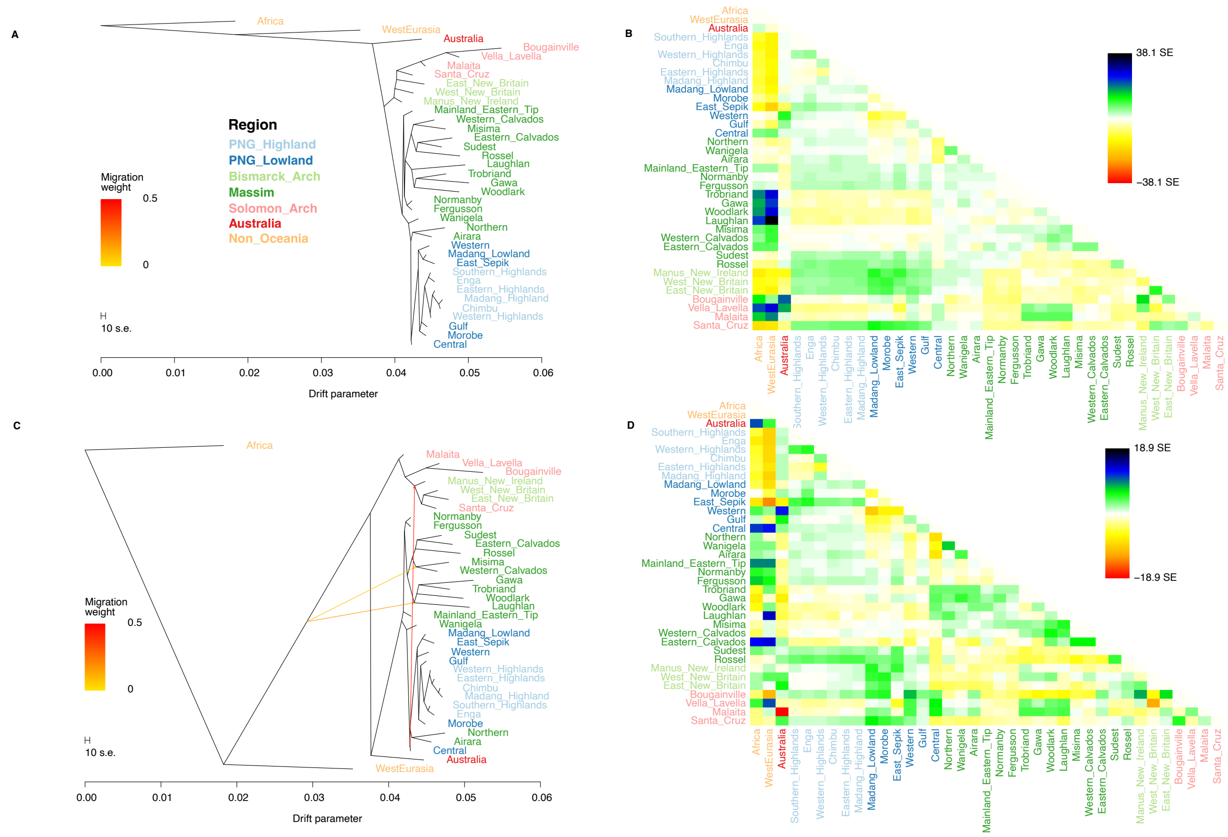

Fig. S16. Papuan ancestry-specific TreeMix analysis. Trees with 0 and 3 migrations are shown in (A) and (C), respectively; and the heat plots for the residuals (expressed in standard errors) are shown in (B) and (D), respectively. The groups are colored according to region. The overall tree topologies support distinct Papuan ancestries. Although there is a large reduction in the residuals with 3 migration edges, the tree is still a poor fit to the data, and the migrations inferred are probably artifacts due to the small amount of data and/or residual unmaksed Austronesian ancestry.

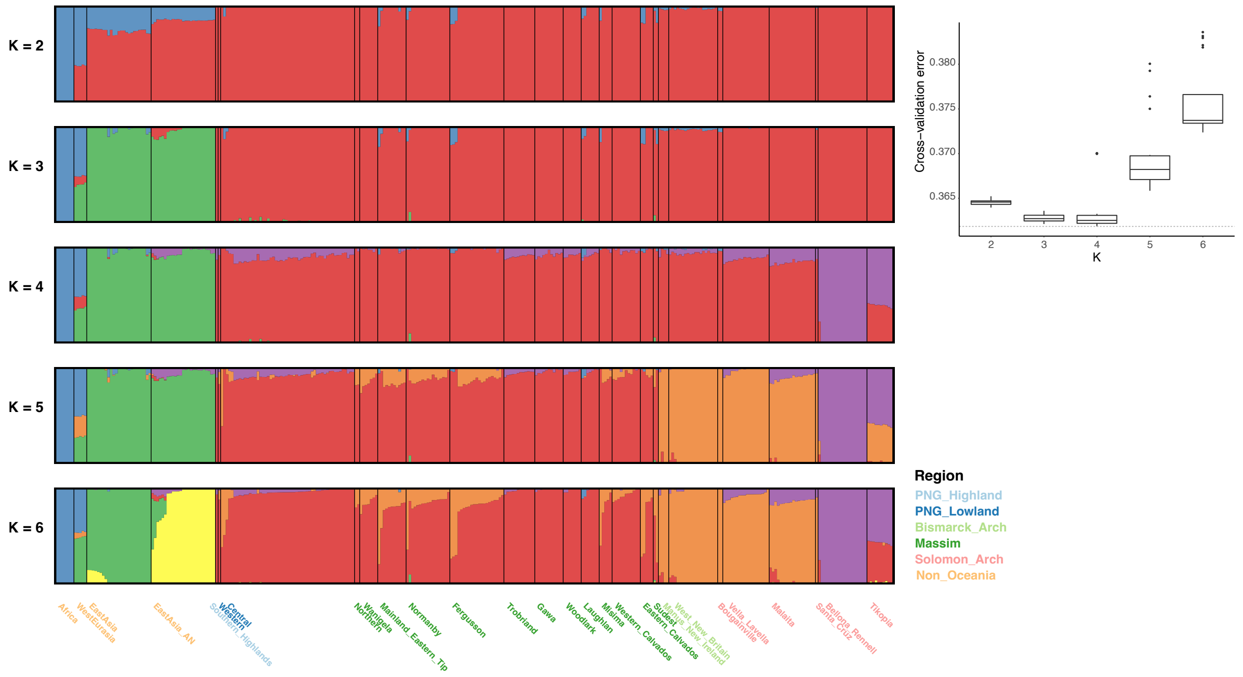

Fig. S17. Austronesian ancestry-specific ADMIXTURE analysis of Oceanian individuals (and comparative non-Oceanian individuals). On the left are the results for K = 2 to K = 6 while the plot on the top right shows the cross-validation errors for 20 runs for each K value. The group labels are colored according to region.

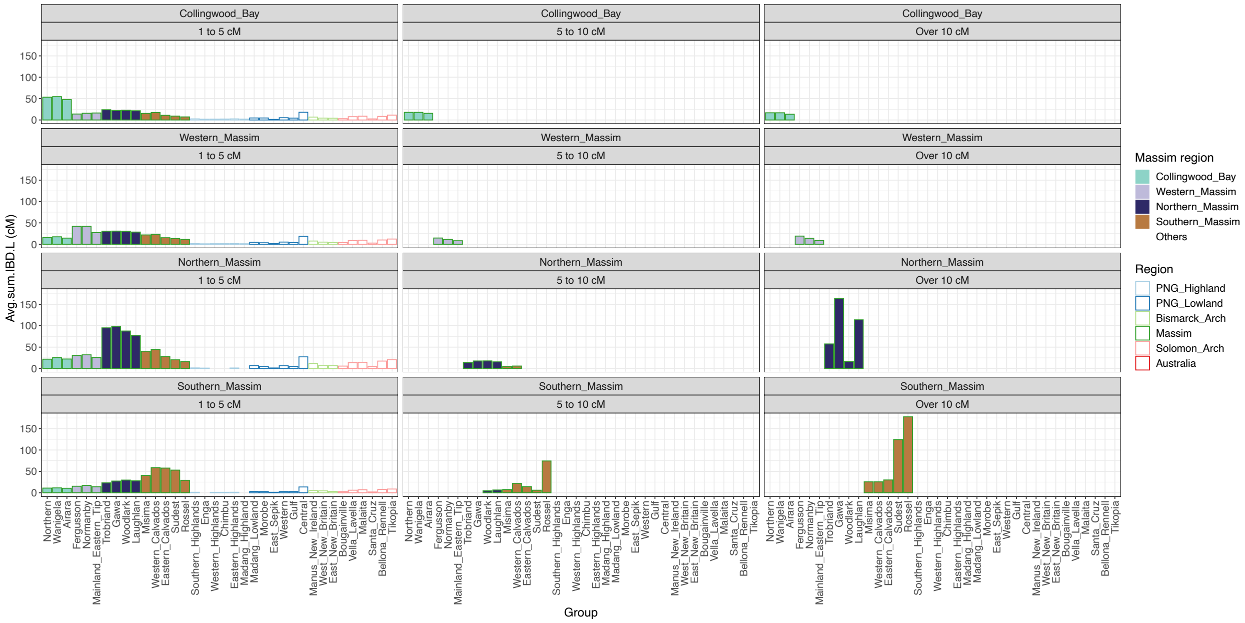

Fig. S18. Quantification of all IBD sharing between Massim region groups and Oceanian groups. The average summed IBD length between Massim regional groups (in rows) and all Oceanian groups, for different size ranges (in columns), is depicted in the bar plots; filled bars are colored according to Massim region while the outline color of the empty bars indicates the Oceania region.

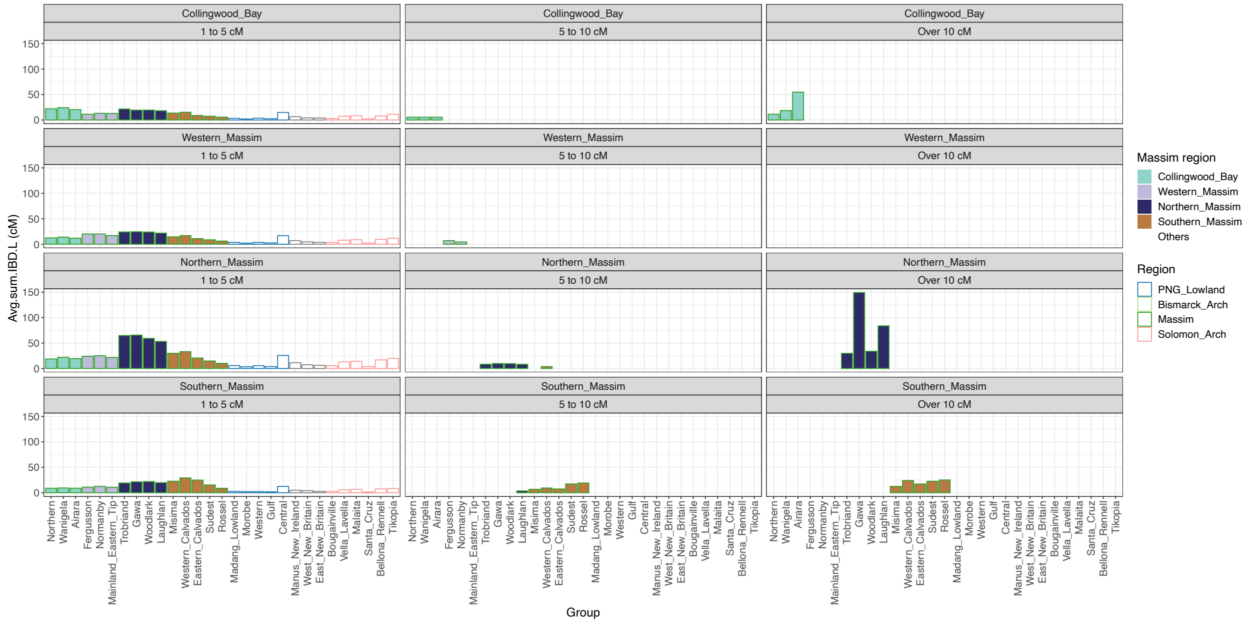

Fig. S19. Quantification of Austronesian ancestry-specific IBD sharing between Massim region groups and Oceanian groups. The average summed Austronesian ancestry-specific IBD length between Massim regional groups (in rows) and all Oceanian groups, for different size ranges (in columns), is depicted in the bar plots; filled bars are colored according to Massim region while the outline color of the empty bars indicates the Oceania region.

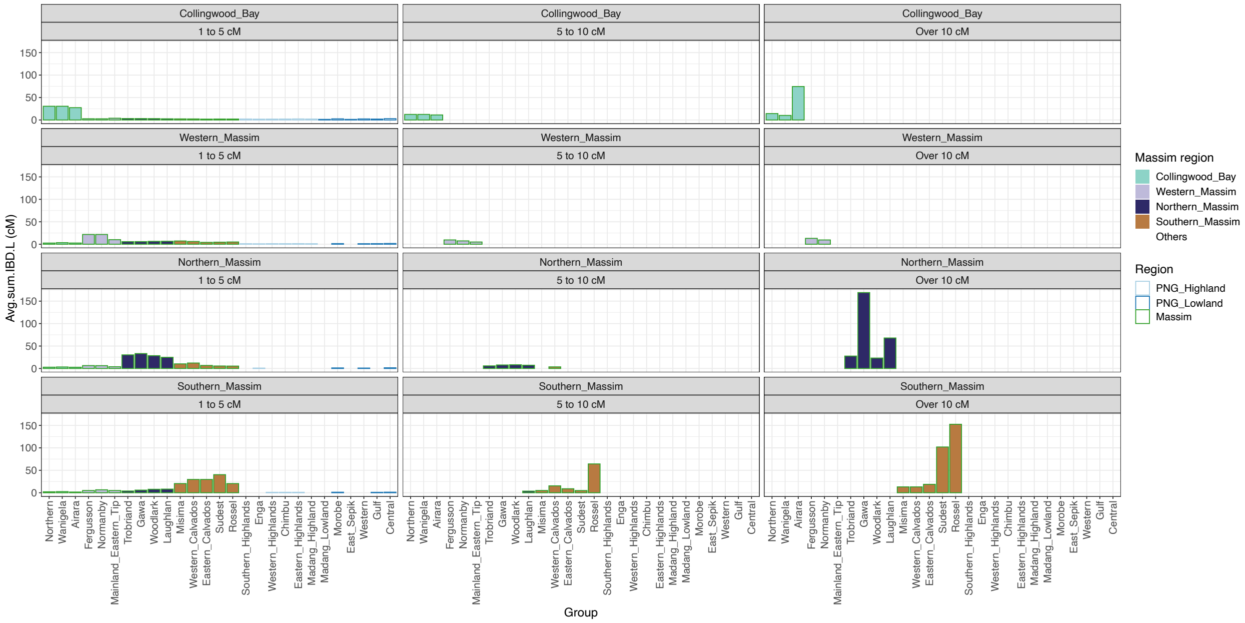

Fig. S20. Quantification of Papuan ancestry-specific IBD sharing between Massim region groups and Oceanian groups. The average summed Papuan ancestry-specific IBD length between Massim regional groups (in rows) and all Oceanian groups, for different size ranges (in columns), is depicted in the bar plots; filled bars are colored according to Massim region while the outline color of the empty bars indicates the Oceania region.

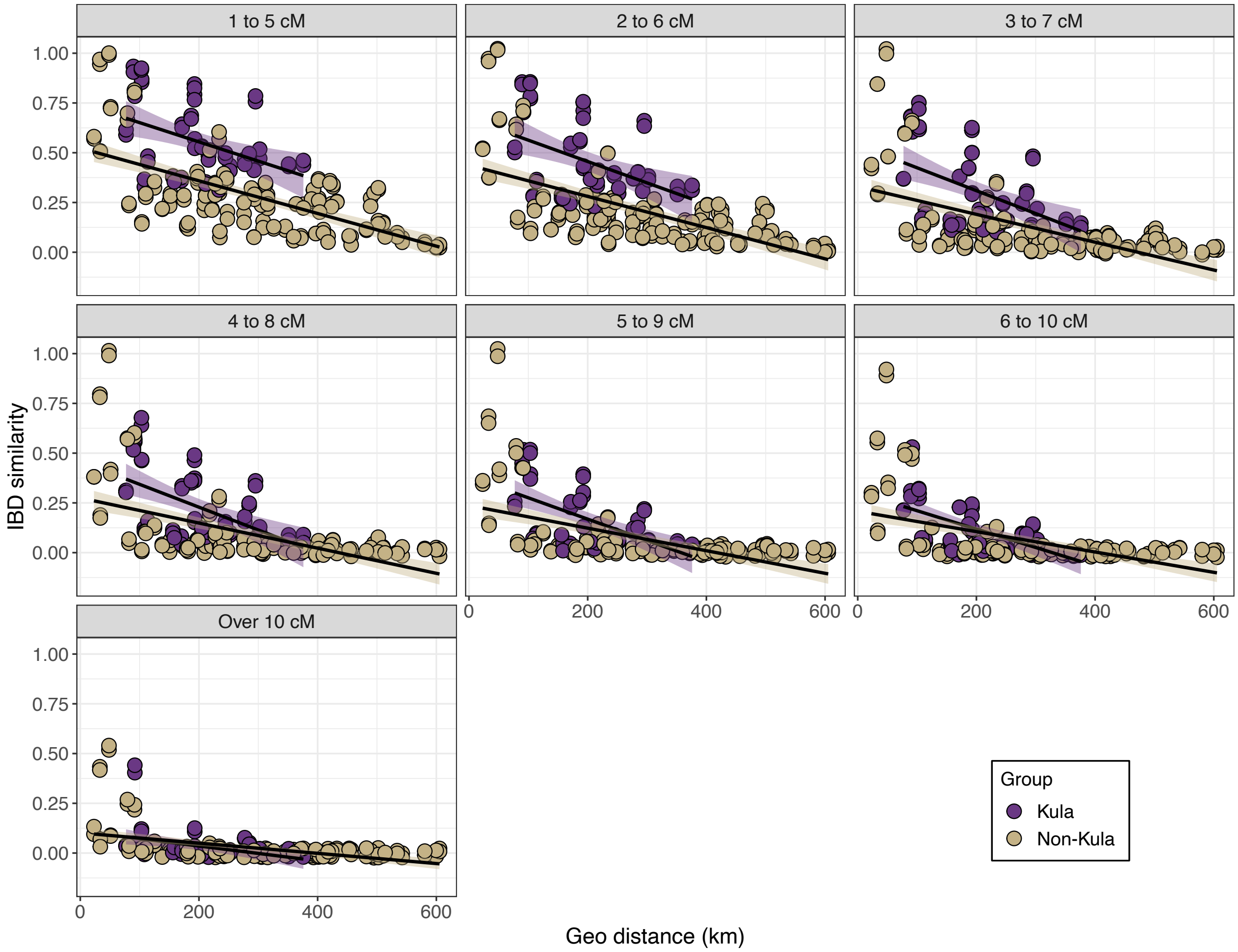

Fig. S21. IBD similarities of Kula- vs. non-Kula groups through space and time. Each point represents the IBD similarity calculated between a pair of Kula (purple) or non-Kula- (brown) groups. Points with the same value were slightly jittered for visibility. The geographic distance (km) between pairs of groups is shown on the x-axis. Two regression lines with 95% confidence interval were separately calculated for the Kula or non-Kula points. Results calculated from different IBD size intervals are shown in different panels. These intervals (from 1-5 to over 10 cM) approximately correspond to ~2.7, ~1.5, ~1.1, ~0.8, ~0.7, ~0.6, and ~0.2 thousand year ago (kya).

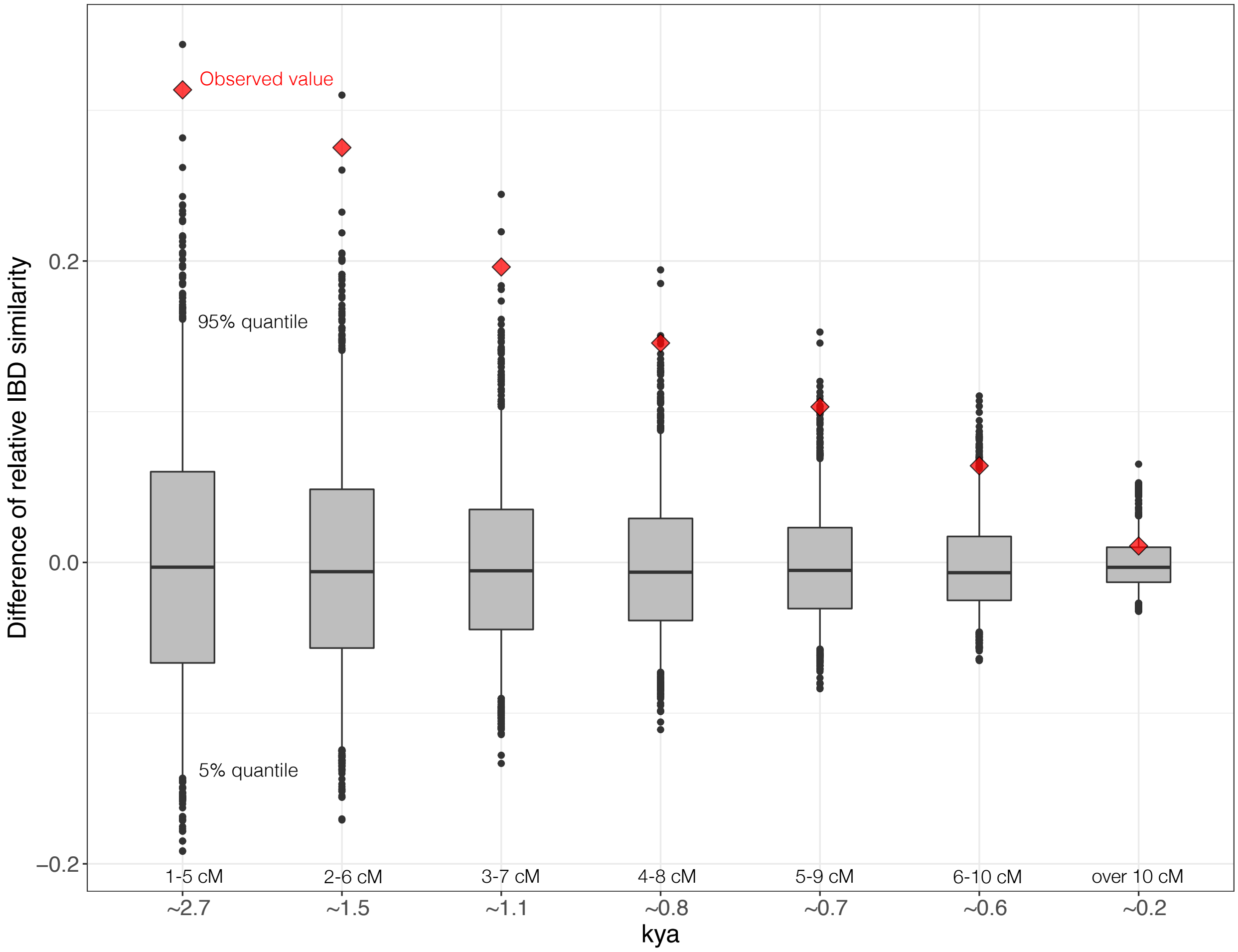

Fig. S22. Significance of the difference in relative IBD similarities between the Kula and non-Kula groups. The red diamond is the observed value, while the boxplot is the distribution of simulated values obtained by randomly assigning groups as Kula or non-Kula. The upper whisker denotes the 95% quantile while the lower whisker denotes the 5% quantile.

Supplementary Tables

Table S1. Metadata for the samples used in this study. In the QC (quality control) column, samples were filtered by the indicated criteria in abbreviations, Kin: kinship up to 1st degree, Imiss: Individual with >5% missing data, PC: PCA outliers, Mixed: individual with parents speaking different languages or coming from different locations.

Individual_ID	Population	Label_in_analysis	PNG_Province	District_Village_Island	Area	Region	Language_Family	Language	Longitude	Latitude	Reference	QC
MB_Ai01	Arifama-Miniafia	Airara	NORTHERN	Airara	Massim	Oceania	Austronesian	Arifama-Miniafia	149.3	-9.51	This_study	PASS
MB_Ai03	Arifama-Miniafia	Airara	NORTHERN	Airara	Massim	Oceania	Austronesian	Arifama-Miniafia	149.3	-9.51	This_study	PASS
MB_Ai05	Arifama-Miniafia	Airara	NORTHERN	Airara	Massim	Oceania	Austronesian	Arifama-Miniafia	149.3	-9.51	This_study	PASS
MB_Ai07	Arifama-Miniafia	Airara	NORTHERN	Airara	Massim	Oceania	Austronesian	Arifama-Miniafia	149.3	-9.51	This_study	PASS
MB_Ai09	Arifama-Miniafia	Airara	NORTHERN	Airara	Massim	Oceania	Austronesian	Arifama-Miniafia	149.3	-9.51	This_study	PASS
MB_Ai12	Arifama-Miniafia	Airara	NORTHERN	Airara	Massim	Oceania	Austronesian	Arifama-Miniafia	149.3	-9.51	This_study	PASS
MB_Ai14	Arifama-Miniafia	Airara	NORTHERN	Airara	Massim	Oceania	Austronesian	Arifama-Miniafia	149.3	-9.51	This_study	PASS
MB_Ai15	Arifama-Miniafia	Airara	NORTHERN	Airara	Massim	Oceania	Austronesian	Arifama-Miniafia	149.3	-9.51	This_study	PASS
MB_Ai17	Arifama-Miniafia	Airara	NORTHERN	Airara	Massim	Oceania	Austronesian	Arifama-Miniafia	149.3	-9.51	This_study	PASS
MB_Ai18	Arifama-Miniafia	Airara	NORTHERN	Airara	Massim	Oceania	Austronesian	Arifama-Miniafia	149.3	-9.51	This_study	PASS
MB_Ai24	Arifama-Miniafia	Airara	NORTHERN	Airara	Massim	Oceania	Austronesian	Arifama-Miniafia	149.3	-9.51	This_study	PASS
MB_Ai25	Arifama-Miniafia	Airara	NORTHERN	Airara	Massim	Oceania	Austronesian	Arifama-Miniafia	149.3	-9.51	This_study	PASS
MB_Ai29	Arifama-Miniafia	Airara	NORTHERN	Airara	Massim	Oceania	Austronesian	Arifama-Miniafia	149.3	-9.51	This_study	PASS
MB_Ai31	Arifama-Miniafia	Airara	NORTHERN	Airara	Massim	Oceania	Austronesian	Arifama-Miniafia	149.3	-9.51	This_study	PASS
MB_Ai32	Arifama-Miniafia	Airara	NORTHERN	Airara	Massim	Oceania	Austronesian	Arifama-Miniafia	149.3	-9.51	This_study	PASS
MB_Ai33	Arifama-Miniafia	Airara	NORTHERN	Airara	Massim	Oceania	Austronesian	Arifama-Miniafia	149.3	-9.51	This_study	PASS
MB_Ai36	Arifama-Miniafia	Airara	NORTHERN	Airara	Massim	Oceania	Austronesian	Arifama-Miniafia	149.3	-9.51	This_study	PASS
MB_Ai38	Arifama-Miniafia	Airara	NORTHERN	Airara	Massim	Oceania	Austronesian	Arifama-Miniafia	149.3	-9.51	This_study	PASS
MB_Ai39	Arifama-Miniafia	Airara	NORTHERN	Airara	Massim	Oceania	Austronesian	Arifama-Miniafia	149.3	-9.51	This_study	PASS
MB_Ha49	Nimoa	Eastern_Calvados	MILNE_BAY	Eastern_Calvados_Dadahai-Kuanak-Nimoa-Joannet-Panawina-Sabarl-Wanim	Massim	Oceania	Austronesian	Nimoa	153.06	-11.16	This_study	PASS
MB_Ni04	Nimoa	Eastern_Calvados	MILNE_BAY	Eastern_Calvados_Dadahai-Kuanak-Nimoa-Joannet-Panawina-Sabarl-Wanim	Massim	Oceania	Austronesian	Nimoa	153.25	-11.3	This_study	PASS
MB_Ni08	Nimoa	Eastern_Calvados	MILNE_BAY	Eastern_Calvados_Dadahai-Kuanak-Nimoa-Joannet-Panawina-Sabarl-Wanim	Massim	Oceania	Austronesian	Nimoa	153.25	-11.3	This_study	PASS
MB_Ni23	Nimoa	Eastern_Calvados	MILNE_BAY	Eastern_Calvados_Dadahai-Kuanak-Nimoa-Joannet-Panawina-Sabarl-Wanim	Massim	Oceania	Austronesian	Nimoa	153.25	-11.3	This_study	PASS
MB_Ni31	Nimoa	Eastern_Calvados	MILNE_BAY	Eastern_Calvados_Dadahai-Kuanak-Nimoa-Joannet-Panawina-Sabarl-Wanim	Massim	Oceania	Austronesian	Nimoa	153.25	-11.3	This_study	PASS
MB_Mo05	Misima-Paneati_WC	Western_Calvados	MILNE_BAY	Western_Calvados_Motorina-Bagaman-Utian-Panaumala	Massim	Oceania	Austronesian	Misima-Paneati	152.57	-11.08	This_study	PASS
MB_Mo08	Misima-Paneati_WC	Western_Calvados	MILNE_BAY	Western_Calvados_Motorina-Bagaman-Utian-Panaumala	Massim	Oceania	Austronesian	Misima-Paneati	152.57	-11.08	This_study	PASS
MB_Mo10	Misima-Paneati_WC	Western_Calvados	MILNE_BAY	Western_Calvados_Motorina-Bagaman-Utian-Panaumala	Massim	Oceania	Austronesian	Misima-Paneati	152.57	-11.08	This_study	PASS

MB_Mo13	Misima-Paneati_WC	Western_Calvados	MILNE_BAY	Western_Calvados_Motorina-Bagaman-Utian-Panaumala	Massim	Oceania	Austronesian	Misima-Paneati	152.57	-11.08	This_study	PASS
MB_Mo14	Misima-Paneati_WC	Western_Calvados	MILNE_BAY	Western_Calvados_Motorina-Bagaman-Utian-Panaumala	Massim	Oceania	Austronesian	Misima-Paneati	152.57	-11.08	This_study	PASS
MB_Mo15	Misima-Paneati_WC	Western_Calvados	MILNE_BAY	Western_Calvados_Motorina-Bagaman-Utian-Panaumala	Massim	Oceania	Austronesian	Misima-Paneati	152.57	-11.08	This_study	PASS
MB_Mo19	Misima-Paneati_WC	Western_Calvados	MILNE_BAY	Western_Calvados_Motorina-Bagaman-Utian-Panaumala	Massim	Oceania	Austronesian	Misima-Paneati	152.57	-11.08	This_study	PASS
MB_Mo20	Misima-Paneati_WC	Western_Calvados	MILNE_BAY	Western_Calvados_Motorina-Bagaman-Utian-Panaumala	Massim	Oceania	Austronesian	Misima-Paneati	152.57	-11.08	This_study	PASS
MB_Mo23	Misima-Paneati_WC	Western_Calvados	MILNE_BAY	Western_Calvados_Motorina-Bagaman-Utian-Panaumala	Massim	Oceania	Austronesian	Misima-Paneati	152.57	-11.08	This_study	PASS
MB_Mo25	Misima-Paneati_WC	Western_Calvados	MILNE_BAY	Western_Calvados_Motorina-Bagaman-Utian-Panaumala	Massim	Oceania	Austronesian	Misima-Paneati	152.57	-11.08	This_study	PASS
MB_Mo26	Misima-Paneati_WC	Western_Calvados	MILNE_BAY	Western_Calvados_Motorina-Bagaman-Utian-Panaumala	Massim	Oceania	Austronesian	Misima-Paneati	152.57	-11.08	This_study	PASS
MB_Fe10	Molima	Fergusson	MILNE_BAY	Fergusson-Dobu-Goodenough	Massim	Oceania	Austronesian	Molima	150.54	-9.46	This_study	PASS
MB_Fe11	Molima	Fergusson	MILNE_BAY	Fergusson-Dobu-Goodenough	Massim	Oceania	Austronesian	Molima	150.54	-9.46	This_study	PASS
MB_Fe12	Molima	Fergusson	MILNE_BAY	Fergusson-Dobu-Goodenough	Massim	Oceania	Austronesian	Molima	150.54	-9.46	This_study	PASS
MB_Fe15	Molima	Fergusson	MILNE_BAY	Fergusson-Dobu-Goodenough	Massim	Oceania	Austronesian	Molima	150.54	-9.46	This_study	PASS
MB_Fe16	Molima	Fergusson	MILNE_BAY	Fergusson-Dobu-Goodenough	Massim	Oceania	Austronesian	Molima	150.54	-9.46	This_study	PASS
MB_Fe18	Molima	Fergusson	MILNE_BAY	Fergusson-Dobu-Goodenough	Massim	Oceania	Austronesian	Molima	150.54	-9.46	This_study	PASS
MB_Fe19	Molima	Fergusson	MILNE_BAY	Fergusson-Dobu-Goodenough	Massim	Oceania	Austronesian	Molima	150.54	-9.46	This_study	PASS
MB_Fe20	Molima	Fergusson	MILNE_BAY	Fergusson-Dobu-Goodenough	Massim	Oceania	Austronesian	Molima	150.54	-9.46	This_study	PASS
MB_Fe21	Molima	Fergusson	MILNE_BAY	Fergusson-Dobu-Goodenough	Massim	Oceania	Austronesian	Molima	150.54	-9.46	This_study	PASS
MB_Fe24	Molima	Fergusson	MILNE_BAY	Fergusson-Dobu-Goodenough	Massim	Oceania	Austronesian	Molima	150.54	-9.46	This_study	PASS
MB_Fe26	Molima	Fergusson	MILNE_BAY	Fergusson-Dobu-Goodenough	Massim	Oceania	Austronesian	Molima	150.54	-9.46	This_study	PASS
MB_Ha12	Molima	Fergusson	MILNE_BAY	Fergusson-Dobu-Goodenough	Massim	Oceania	Austronesian	Molima	150.54	-9.46	This_study	PASS
MB_Ha14	Molima	Fergusson	MILNE_BAY	Fergusson-Dobu-Goodenough	Massim	Oceania	Austronesian	Molima	150.54	-9.46	This_study	PASS
MB_Ha18	Molima	Fergusson	MILNE_BAY	Fergusson-Dobu-Goodenough	Massim	Oceania	Austronesian	Molima	150.54	-9.46	This_study	PASS
MB_Ha21	Molima	Fergusson	MILNE_BAY	Fergusson-Dobu-Goodenough	Massim	Oceania	Austronesian	Molima	150.54	-9.46	This_study	PASS
MB_Ha30	Molima	Fergusson	MILNE_BAY	Fergusson-Dobu-Goodenough	Massim	Oceania	Austronesian	Molima	150.54	-9.46	This_study	PASS
MB_Ha45	Molima	Fergusson	MILNE_BAY	Fergusson-Dobu-Goodenough	Massim	Oceania	Austronesian	Molima	150.54	-9.46	This_study	PASS
MB_Ha53	Molima	Fergusson	MILNE_BAY	Fergusson-Dobu-Goodenough	Massim	Oceania	Austronesian	Molima	150.54	-9.46	This_study	PASS
MB_Ha54	Molima	Fergusson	MILNE_BAY	Fergusson-Dobu-Goodenough	Massim	Oceania	Austronesian	Molima	150.54	-9.46	This_study	PASS
MB_Ha62	Molima	Fergusson	MILNE_BAY	Fergusson-Dobu-Goodenough	Massim	Oceania	Austronesian	Molima	150.54	-9.46	This_study	PASS
MB_Ha67	Molima	Fergusson	MILNE_BAY	Fergusson-Dobu-Goodenough	Massim	Oceania	Austronesian	Molima	150.54	-9.46	This_study	Kin
MB_Ha68	Molima	Fergusson	MILNE_BAY	Fergusson-Dobu-Goodenough	Massim	Oceania	Austronesian	Molima	150.54	-9.46	This_study	PASS
MB_Ha80	Molima	Fergusson	MILNE_BAY	Fergusson-Dobu-Goodenough	Massim	Oceania	Austronesian	Molima	150.54	-9.46	This_study	PASS

MB_Ga03	Muyuw_Gawa	Gawa	MILNE_BAY	Gawa	Massim	Oceania	Austronesian	Muyuw	151.99	-8.97	This_study	PASS
MB_Ga04	Muyuw_Gawa	Gawa	MILNE_BAY	Gawa	Massim	Oceania	Austronesian	Muyuw	151.99	-8.97	This_study	PASS
MB_Ga06	Muyuw_Gawa	Gawa	MILNE_BAY	Gawa	Massim	Oceania	Austronesian	Muyuw	151.99	-8.97	This_study	PASS (2nd degree Kinship)
MB_Ga09	Muyuw_Gawa	Gawa	MILNE_BAY	Gawa	Massim	Oceania	Austronesian	Muyuw	151.99	-8.97	This_study	PASS
MB_Ga10	Muyuw_Gawa	Gawa	MILNE_BAY	Gawa	Massim	Oceania	Austronesian	Muyuw	151.99	-8.97	This_study	PASS
MB_Ga12	Muyuw_Gawa	Gawa	MILNE_BAY	Gawa	Massim	Oceania	Austronesian	Muyuw	151.99	-8.97	This_study	PASS
MB_Ga14	Muyuw_Gawa	Gawa	MILNE_BAY	Gawa	Massim	Oceania	Austronesian	Muyuw	151.99	-8.97	This_study	PASS
MB_Ga15	Muyuw_Gawa	Gawa	MILNE_BAY	Gawa	Massim	Oceania	Austronesian	Muyuw	151.99	-8.97	This_study	PASS
MB_Ga16	Muyuw_Gawa	Gawa	MILNE_BAY	Gawa	Massim	Oceania	Austronesian	Muyuw	151.99	-8.97	This_study	PASS
MB_Ga17	Muyuw_Gawa	Gawa	MILNE_BAY	Gawa	Massim	Oceania	Austronesian	Muyuw	151.99	-8.97	This_study	PASS
MB_Ga18	Muyuw_Gawa	Gawa	MILNE_BAY	Gawa	Massim	Oceania	Austronesian	Muyuw	151.99	-8.97	This_study	PASS
MB_Ha07	Tawala	Mainland_Eastern_Tip	MILNE_BAY	Mline_Bay_mainland_eastern_tip	Massim	Oceania	Austronesian	Tawala	150.53	-10.49	This_study	PASS
MB_Ha10	Tawala	Mainland_Eastern_Tip	MILNE_BAY	Mline_Bay_mainland_eastern_tip	Massim	Oceania	Austronesian	Tawala	150.53	-10.49	This_study	PASS
MB_Ha13	Tawala	Mainland_Eastern_Tip	MILNE_BAY	Mline_Bay_mainland_eastern_tip	Massim	Oceania	Austronesian	Tawala	150.53	-10.49	This_study	PASS
MB_Ha17	Tawala	Mainland_Eastern_Tip	MILNE_BAY	Mline_Bay_mainland_eastern_tip	Massim	Oceania	Austronesian	Tawala	150.53	-10.49	This_study	PASS
MB_Ha32	Tawala	Mainland_Eastern_Tip	MILNE_BAY	Mline_Bay_mainland_eastern_tip	Massim	Oceania	Austronesian	Tawala	150.53	-10.49	This_study	PASS
MB_Ha33	Tawala	Mainland_Eastern_Tip	MILNE_BAY	Mline_Bay_mainland_eastern_tip	Massim	Oceania	Austronesian	Tawala	150.53	-10.49	This_study	PASS
MB_Ha42	Tawala	Mainland_Eastern_Tip	MILNE_BAY	Mline_Bay_mainland_eastern_tip	Massim	Oceania	Austronesian	Tawala	150.53	-10.49	This_study	PASS
MB_Ha50	Tawala	Mainland_Eastern_Tip	MILNE_BAY	Mline_Bay_mainland_eastern_tip	Massim	Oceania	Austronesian	Tawala	150.53	-10.49	This_study	Imiss
MB_Ha51	Tawala	Mainland_Eastern_Tip	MILNE_BAY	Mline_Bay_mainland_eastern_tip	Massim	Oceania	Austronesian	Tawala	150.53	-10.49	This_study	PASS
MB_Ha64	Tawala	Mainland_Eastern_Tip	MILNE_BAY	Mline_Bay_mainland_eastern_tip	Massim	Oceania	Austronesian	Tawala	150.53	-10.49	This_study	PASS
MB_Ha70	Tawala	Mainland_Eastern_Tip	MILNE_BAY	Mline_Bay_mainland_eastern_tip	Massim	Oceania	Austronesian	Tawala	150.53	-10.49	This_study	PASS
MB_Ha74	Tawala	Mainland_Eastern_Tip	MILNE_BAY	Mline_Bay_mainland_eastern_tip	Massim	Oceania	Austronesian	Tawala	150.53	-10.49	This_study	PASS
MB_Ji014	Tawala	Mainland_Eastern_Tip	MILNE_BAY	Mline_Bay_mainland_eastern_tip	Massim	Oceania	Austronesian	Tawala	150.53	-10.49	This_study	PASS
MB_Mo02	Misima-Paneati	Misima	MILNE_BAY	Misima-Paneati-Panapompom-Kimuta	Massim	Oceania	Austronesian	Misima-Paneati	152.72	-10.67	This_study	PASS
MB_Mo16	Misima-Paneati	Misima	MILNE_BAY	Misima-Paneati-Panapompom-Kimuta	Massim	Oceania	Austronesian	Misima-Paneati	152.72	-10.67	This_study	PASS
MB_Mo18	Misima-Paneati	Misima	MILNE_BAY	Misima-Paneati-Panapompom-Kimuta	Massim	Oceania	Austronesian	Misima-Paneati	152.72	-10.67	This_study	PASS
MB_Ni38	Misima-Paneati	Misima	MILNE_BAY	Misima-Paneati-Panapompom-Kimuta	Massim	Oceania	Austronesian	Misima-Paneati	152.72	-10.67	This_study	PASS
MB_Fe02	Bunana	Normanby	MILNE_BAY	Normanby	Massim	Oceania	Austronesian	Bunana	151.09	-10.08	This_study	PASS
MB_Fe03	Bunana	Normanby	MILNE_BAY	Normanby	Massim	Oceania	Austronesian	Bunana	151.09	-10.08	This_study	PASS
MB_Fe04	Bunana	Normanby	MILNE_BAY	Normanby	Massim	Oceania	Austronesian	Bunana	151.09	-10.08	This_study	PASS
MB_Fe23	Bunana	Normanby	MILNE_BAY	Normanby	Massim	Oceania	Austronesian	Bunana	151.09	-10.08	This_study	PASS
MB_Ha05	Bunana	Normanby	MILNE_BAY	Normanby	Massim	Oceania	Austronesian	Bunana	151.09	-10.08	This_study	PASS
MB_Ha08	Bunana	Normanby	MILNE_BAY	Normanby	Massim	Oceania	Austronesian	Bunana	151.09	-10.08	This_study	PASS
MB_Ha09	Bunana	Normanby	MILNE_BAY	Normanby	Massim	Oceania	Austronesian	Bunana	151.09	-10.08	This_study	PASS
MB_Ha11	Bunana	Normanby	MILNE_BAY	Normanby	Massim	Oceania	Austronesian	Bunana	151.09	-10.08	This_study	PASS
MB_Ha20	Bunana	Normanby	MILNE_BAY	Normanby	Massim	Oceania	Austronesian	Bunana	151.09	-10.08	This_study	PASS
MB_Ha26	Bunana	Normanby	MILNE_BAY	Normanby	Massim	Oceania	Austronesian	Bunana	151.09	-10.08	This_study	PASS
MB_Ha28	Bunana	Normanby	MILNE_BAY	Normanby	Massim	Oceania	Austronesian	Bunana	151.09	-10.08	This_study	PASS
MB_Ha35	Bunana	Normanby	MILNE_BAY	Normanby	Massim	Oceania	Austronesian	Bunana	151.09	-10.08	This_study	PASS
MB_Ha56	Bunana	Normanby	MILNE_BAY	Normanby	Massim	Oceania	Austronesian	Bunana	151.09	-10.08	This_study	PASS
MB_Ha58	Bunana	Normanby	MILNE_BAY	Normanby	Massim	Oceania	Austronesian	Bunana	151.09	-10.08	This_study	PASS
MB_Ha76	Bunana	Normanby	MILNE_BAY	Normanby	Massim	Oceania	Austronesian	Bunana	151.09	-10.08	This_study	PASS

MB_Ha84	Bunana	Normanby	MILNE_BAY	Normanby	Massim	Oceania	Austronesian	Bunana	151.09	-10.08	This_study	PASS
MB_Ha85	Bunana	Normanby	MILNE_BAY	Normanby	Massim	Oceania	Austronesian	Bunana	151.09	-10.08	This_study	PASS
MB_Su02	Sudest	Sudest	MILNE_BAY	Sudest	Massim	Oceania	Austronesian	Sudest	153.46	-11.52	This_study	PASS
MB_Su03	Sudest	Sudest	MILNE_BAY	Sudest	Massim	Oceania	Austronesian	Sudest	153.46	-11.52	This_study	PASS
MB_Su04	Sudest	Sudest	MILNE_BAY	Sudest	Massim	Oceania	Austronesian	Sudest	153.46	-11.52	This_study	PASS
MB_Su06	Sudest	Sudest	MILNE_BAY	Sudest	Massim	Oceania	Austronesian	Sudest	153.46	-11.52	This_study	PASS
MB_Su07	Sudest	Sudest	MILNE_BAY	Sudest	Massim	Oceania	Austronesian	Sudest	153.46	-11.52	This_study	PASS
MB_Su08	Sudest	Sudest	MILNE_BAY	Sudest	Massim	Oceania	Austronesian	Sudest	153.46	-11.52	This_study	PASS
MB_Su10	Sudest	Sudest	MILNE_BAY	Sudest	Massim	Oceania	Austronesian	Sudest	153.46	-11.52	This_study	PASS
MB_Su12	Sudest	Sudest	MILNE_BAY	Sudest	Massim	Oceania	Austronesian	Sudest	153.46	-11.52	This_study	PASS
MB_Su14	Sudest	Sudest	MILNE_BAY	Sudest	Massim	Oceania	Austronesian	Sudest	153.46	-11.52	This_study	PASS
MB_Su20	Sudest	Sudest	MILNE_BAY	Sudest	Massim	Oceania	Austronesian	Sudest	153.46	-11.52	This_study	PASS
MB_Su22	Sudest	Sudest	MILNE_BAY	Sudest	Massim	Oceania	Austronesian	Sudest	153.46	-11.52	This_study	PASS
MB_Su25	Sudest	Sudest	MILNE_BAY	Sudest	Massim	Oceania	Austronesian	Sudest	153.46	-11.52	This_study	PASS
MB_Su26	Sudest	Sudest	MILNE_BAY	Sudest	Massim	Oceania	Austronesian	Sudest	153.46	-11.52	This_study	PASS
MB_Su28	Sudest	Sudest	MILNE_BAY	Sudest	Massim	Oceania	Austronesian	Sudest	153.46	-11.52	This_study	PASS
MB_Su29	Sudest	Sudest	MILNE_BAY	Sudest	Massim	Oceania	Austronesian	Sudest	153.46	-11.52	This_study	PASS
MB_Su30	Sudest	Sudest	MILNE_BAY	Sudest	Massim	Oceania	Austronesian	Sudest	153.46	-11.52	This_study	PASS
MB_Wa06	Maisin	Wanigela	NORTHERN	Wanigela	Massim	Oceania	Austronesian	Maisin	149.18	-9.34	This_study	PASS
MB_Wa07	Maisin	Wanigela	NORTHERN	Wanigela	Massim	Oceania	Austronesian	Maisin	149.18	-9.34	This_study	PASS
MB_Wa09	Maisin	Wanigela	NORTHERN	Wanigela	Massim	Oceania	Austronesian	Maisin	149.18	-9.34	This_study	PASS
MB_Wa11	Maisin	Wanigela	NORTHERN	Wanigela	Massim	Oceania	Austronesian	Maisin	149.18	-9.34	This_study	PASS
MB_Wa13	Maisin	Wanigela	NORTHERN	Wanigela	Massim	Oceania	Austronesian	Maisin	149.18	-9.34	This_study	PASS
MB_Wa14	Maisin	Wanigela	NORTHERN	Wanigela	Massim	Oceania	Austronesian	Maisin	149.18	-9.34	This_study	PASS
MB_Wa15	Maisin	Wanigela	NORTHERN	Wanigela	Massim	Oceania	Austronesian	Maisin	149.18	-9.34	This_study	PASS
MB_Wa16	Maisin	Wanigela	NORTHERN	Wanigela	Massim	Oceania	Austronesian	Maisin	149.18	-9.34	This_study	PASS
MB_Wa18	Maisin	Wanigela	NORTHERN	Wanigela	Massim	Oceania	Austronesian	Maisin	149.18	-9.34	This_study	PASS
MB_Wa19	Maisin	Wanigela	NORTHERN	Wanigela	Massim	Oceania	Austronesian	Maisin	149.18	-9.34	This_study	PASS
MB_Wa20	Maisin	Wanigela	NORTHERN	Wanigela	Massim	Oceania	Austronesian	Maisin	149.18	-9.34	This_study	PASS
MB_Wa21	Maisin	Wanigela	NORTHERN	Wanigela	Massim	Oceania	Austronesian	Maisin	149.18	-9.34	This_study	PASS
MB_Wa22	Maisin	Wanigela	NORTHERN	Wanigela	Massim	Oceania	Austronesian	Maisin	149.18	-9.34	This_study	PASS
MB_Wa23	Maisin	Wanigela	NORTHERN	Wanigela	Massim	Oceania	Austronesian	Maisin	149.18	-9.34	This_study	PASS
MB_Wa26	Maisin	Wanigela	NORTHERN	Wanigela	Massim	Oceania	Austronesian	Maisin	149.18	-9.34	This_study	PASS
MB_Bu01	Budibud	Laughlan	MILNE_BAY	Laughlan	Massim	Oceania	Austronesian	Budibud	153.69	-9.29	This_study	PASS
MB_Bu02	Budibud	Laughlan	MILNE_BAY	Laughlan	Massim	Oceania	Austronesian	Budibud	153.69	-9.29	This_study	PASS
MB_Bu05	Budibud	Laughlan	MILNE_BAY	Laughlan	Massim	Oceania	Austronesian	Budibud	153.69	-9.29	This_study	PASS
MB_Bu06	Budibud	Laughlan	MILNE_BAY	Laughlan	Massim	Oceania	Austronesian	Budibud	153.69	-9.29	This_study	PASS
MB_Bu07	Budibud	Laughlan	MILNE_BAY	Laughlan	Massim	Oceania	Austronesian	Budibud	153.69	-9.29	This_study	PASS
MB_Bu09	Budibud	Laughlan	MILNE_BAY	Laughlan	Massim	Oceania	Austronesian	Budibud	153.69	-9.29	This_study	PASS
MB_Ha59	Budibud	Laughlan	MILNE_BAY	Laughlan	Massim	Oceania	Austronesian	Budibud	153.69	-9.29	This_study	PASS
MB_Wo04	Muyuw_Woodlark	Woodlark	MILNE_BAY	Woodlark	Massim	Oceania	Austronesian	Muyuw	152.78	-9.12	This_study	PASS
MB_Wo06	Muyuw_Woodlark	Woodlark	MILNE_BAY	Woodlark	Massim	Oceania	Austronesian	Muyuw	152.78	-9.12	This_study	PASS
MB_Wo09	Muyuw_Woodlark	Woodlark	MILNE_BAY	Woodlark	Massim	Oceania	Austronesian	Muyuw	152.78	-9.12	This_study	PASS
MB_Wo10	Muyuw_Woodlark	Woodlark	MILNE_BAY	Woodlark	Massim	Oceania	Austronesian	Muyuw	152.78	-9.12	This_study	PASS
MB_Wo14	Muyuw_Woodlark	Woodlark	MILNE_BAY	Woodlark	Massim	Oceania	Austronesian	Muyuw	152.78	-9.12	This_study	PASS
MB_Wo18	Muyuw_Woodlark	Woodlark	MILNE_BAY	Woodlark	Massim	Oceania	Austronesian	Muyuw	152.78	-9.12	This_study	PASS
MB_Wo20	Muyuw_Woodlark	Woodlark	MILNE_BAY	Woodlark	Massim	Oceania	Austronesian	Muyuw	152.78	-9.12	This_study	PASS
MB_Ga01	Kilivila	Trobriand	MILNE_BAY	Trobriand	Massim	Oceania	Austronesian	Kilivila	151.11	-8.67	This_study	PASS
MB_Wo19	Kilivila	Trobriand	MILNE_BAY	Trobriand	Massim	Oceania	Austronesian	Kilivila	151.11	-8.67	This_study	PASS
MB_Wo21	Kilivila	Trobriand	MILNE_BAY	Trobriand	Massim	Oceania	Austronesian	Kilivila	151.11	-8.67	This_study	PASS
T02	Kilivila	Trobriand	MILNE_BAY	Trobriand	Massim	Oceania	Austronesian	Kilivila	151.11	-8.67	This_study	PASS
T09	Kilivila	Trobriand	MILNE_BAY	Trobriand	Massim	Oceania	Austronesian	Kilivila	151.11	-8.67	This_study	PASS
T16	Kilivila	Trobriand	MILNE_BAY	Trobriand	Massim	Oceania	Austronesian	Kilivila	151.11	-8.67	This_study	PASS
T36	Kilivila	Trobriand	MILNE_BAY	Trobriand	Massim	Oceania	Austronesian	Kilivila	151.11	-8.67	This_study	PASS

T40	Kilivila	Trobriand	MILNE_BAY	Trobriand	Massim	Oceania	Austronesian	Kilivila	151.11	-8.67	This_study	PASS
T47	Kilivila	Trobriand	MILNE_BAY	Trobriand	Massim	Oceania	Austronesian	Kilivila	151.11	-8.67	This_study	PASS
T63	Kilivila	Trobriand	MILNE_BAY	Trobriand	Massim	Oceania	Austronesian	Kilivila	151.11	-8.67	This_study	PASS
T66	Kilivila	Trobriand	MILNE_BAY	Trobriand	Massim	Oceania	Austronesian	Kilivila	151.11	-8.67	This_study	PASS
T68	Kilivila	Trobriand	MILNE_BAY	Trobriand	Massim	Oceania	Austronesian	Kilivila	151.11	-8.67	This_study	PASS
Ji_058	Yele	Rossel	MILNE_BAY	Rossel	Massim	Oceania	Yele-West_New_Britain	Yele	154.18	-11.36	This_study	PASS
Ji_059	Yele	Rossel	MILNE_BAY	Rossel	Massim	Oceania	Yele-West_New_Britain	Yele	154.18	-11.36	This_study	PASS
Ji_060	Yele	Rossel	MILNE_BAY	Rossel	Massim	Oceania	Yele-West_New_Britain	Yele	154.18	-11.36	This_study	PASS
Ji_061	Yele	Rossel	MILNE_BAY	Rossel	Massim	Oceania	Yele-West_New_Britain	Yele	154.18	-11.36	This_study	PASS
Ji_062	Yele	Rossel	MILNE_BAY	Rossel	Massim	Oceania	Yele-West_New_Britain	Yele	154.18	-11.36	This_study	PASS
Ji_063	Yele	Rossel	MILNE_BAY	Rossel	Massim	Oceania	Yele-West_New_Britain	Yele	154.18	-11.36	This_study	PASS
Ji_064	Yele	Rossel	MILNE_BAY	Rossel	Massim	Oceania	Yele-West_New_Britain	Yele	154.18	-11.36	This_study	PASS
Ji_065	Yele	Rossel	MILNE_BAY	Rossel	Massim	Oceania	Yele-West_New_Britain	Yele	154.18	-11.36	This_study	PASS
Ji_066	Yele	Rossel	MILNE_BAY	Rossel	Massim	Oceania	Yele-West_New_Britain	Yele	154.18	-11.36	This_study	PASS
Ji_067	Yele	Rossel	MILNE_BAY	Rossel	Massim	Oceania	Yele-West_New_Britain	Yele	154.18	-11.36	This_study	PASS
Ji_069	Yele	Rossel	MILNE_BAY	Rossel	Massim	Oceania	Yele-West_New_Britain	Yele	154.18	-11.36	This_study	PASS (2nd degree Kinship)
Ji_070	Yele	Rossel	MILNE_BAY	Rossel	Massim	Oceania	Yele-West_New_Britain	Yele	154.18	-11.36	This_study	PASS
Ji_072	Yele	Rossel	MILNE_BAY	Rossel	Massim	Oceania	Yele-West_New_Britain	Yele	154.18	-11.36	This_study	PASS
Ji_073	Yele	Rossel	MILNE_BAY	Rossel	Massim	Oceania	Yele-West_New_Britain	Yele	154.18	-11.36	This_study	PASS
Ji_074	Yele	Rossel	MILNE_BAY	Rossel	Massim	Oceania	Yele-West_New_Britain	Yele	154.18	-11.36	This_study	PASS
Ji_076	Yele	Rossel	MILNE_BAY	Rossel	Massim	Oceania	Yele-West_New_Britain	Yele	154.18	-11.36	This_study	PASS
Ji_077	Yele	Rossel	MILNE_BAY	Rossel	Massim	Oceania	Yele-West_New_Britain	Yele	154.18	-11.36	This_study	PASS
Ji_078	Yele	Rossel	MILNE_BAY	Rossel	Massim	Oceania	Yele-West_New_Britain	Yele	154.18	-11.36	This_study	PASS
Ji_079	Yele	Rossel	MILNE_BAY	Rossel	Massim	Oceania	Yele-West_New_Britain	Yele	154.18	-11.36	This_study	PASS
Ji_081	Yele	Rossel	MILNE_BAY	Rossel	Massim	Oceania	Yele-West_New_Britain	Yele	154.18	-11.36	This_study	PASS
Ji_083	Yele	Rossel	MILNE_BAY	Rossel	Massim	Oceania	Yele-West_New_Britain	Yele	154.18	-11.36	This_study	PASS
Ji_085	Yele	Rossel	MILNE_BAY	Rossel	Massim	Oceania	Yele-West_New_Britain	Yele	154.18	-11.36	This_study	PASS
Ji_086	Yele	Rossel	MILNE_BAY	Rossel	Massim	Oceania	Yele-West_New_Britain	Yele	154.18	-11.36	This_study	Kin
Ji_087	Yele	Rossel	MILNE_BAY	Rossel	Massim	Oceania	Yele-West_New_Britain	Yele	154.18	-11.36	This_study	PASS
Ji_089	Yele	Rossel	MILNE_BAY	Rossel	Massim	Oceania	Yele-West_New_Britain	Yele	154.18	-11.36	This_study	PASS
Ji_090	Yele	Rossel	MILNE_BAY	Rossel	Massim	Oceania	Yele-West_New_Britain	Yele	154.18	-11.36	This_study	PASS
Ji_092	Yele	Rossel	MILNE_BAY	Rossel	Massim	Oceania	Yele-West_New_Britain	Yele	154.18	-11.36	This_study	PASS
Ji_093	Yele	Rossel	MILNE_BAY	Rossel	Massim	Oceania	Yele-West_New_Britain	Yele	154.18	-11.36	This_study	PASS
Ji_094	Yele	Rossel	MILNE_BAY	Rossel	Massim	Oceania	Yele-West_New_Britain	Yele	154.18	-11.36	This_study	PASS
Ji_095	Yele	Rossel	MILNE_BAY	Rossel	Massim	Oceania	Yele-West_New_Britain	Yele	154.18	-11.36	This_study	PASS
Ji_096	Yele	Rossel	MILNE_BAY	Rossel	Massim	Oceania	Yele-West_New_Britain	Yele	154.18	-11.36	This_study	PASS
Ji_097	Yele	Rossel	MILNE_BAY	Rossel	Massim	Oceania	Yele-West_New_Britain	Yele	154.18	-11.36	This_study	PASS (2nd degree Kinship)

Ka01	Purari_Kapuna	Gulf	GULF	Kapuna	PNG_Lowland	Oceania	Trans-New_Guinea	Purari	144.97	-7.58	This_study	PASS
Ka02	Purari_Kapuna	Gulf	GULF	Kapuna	PNG_Lowland	Oceania	Trans-New_Guinea	Purari	144.97	-7.58	This_study	PASS
Ka05	Purari_Kapuna	Gulf	GULF	Kapuna	PNG_Lowland	Oceania	Trans-New_Guinea	Purari	144.97	-7.58	This_study	PASS
Ka10	Purari_Kapuna	Gulf	GULF	Kapuna	PNG_Lowland	Oceania	Trans-New_Guinea	Purari	144.97	-7.58	This_study	PASS
Ka11	Purari_Kapuna	Gulf	GULF	Kapuna	PNG_Lowland	Oceania	Trans-New_Guinea	Purari	144.97	-7.58	This_study	PASS
Ka12	Purari_Kapuna	Gulf	GULF	Kapuna	PNG_Lowland	Oceania	Trans-New_Guinea	Purari	144.97	-7.58	This_study	PASS
Ka13	Purari_Kapuna	Gulf	GULF	Kapuna	PNG_Lowland	Oceania	Trans-New_Guinea	Purari	144.97	-7.58	This_study	PASS
Ka14	Purari_Kapuna	Gulf	GULF	Kapuna	PNG_Lowland	Oceania	Trans-New_Guinea	Purari	144.97	-7.58	This_study	PASS
Ka15	Purari_Kapuna	Gulf	GULF	Kapuna	PNG_Lowland	Oceania	Trans-New_Guinea	Purari	144.97	-7.58	This_study	PASS
Ka16	Purari_Kapuna	Gulf	GULF	Kapuna	PNG_Lowland	Oceania	Trans-New_Guinea	Purari	144.97	-7.58	This_study	PASS
Ka17	Purari_Kapuna	Gulf	GULF	Kapuna	PNG_Lowland	Oceania	Trans-New_Guinea	Purari	144.97	-7.58	This_study	PASS
Ka19	Purari_Kapuna	Gulf	GULF	Kapuna	PNG_Lowland	Oceania	Trans-New_Guinea	Purari	144.97	-7.58	This_study	PASS
Ka20	Purari_Kapuna	Gulf	GULF	Kapuna	PNG_Lowland	Oceania	Trans-New_Guinea	Purari	144.97	-7.58	This_study	PASS
Ka21	Purari_Kapuna	Gulf	GULF	Kapuna	PNG_Lowland	Oceania	Trans-New_Guinea	Purari	144.97	-7.58	This_study	PASS
Ka22	Purari_Kapuna	Gulf	GULF	Kapuna	PNG_Lowland	Oceania	Trans-New_Guinea	Purari	144.97	-7.58	This_study	PASS
Ka23	Purari_Kapuna	Gulf	GULF	Kapuna	PNG_Lowland	Oceania	Trans-New_Guinea	Purari	144.97	-7.58	This_study	PASS
Ka24	Purari_Kapuna	Gulf	GULF	Kapuna	PNG_Lowland	Oceania	Trans-New_Guinea	Purari	144.97	-7.58	This_study	PASS
Ka25	Purari_Kapuna	Gulf	GULF	Kapuna	PNG_Lowland	Oceania	Trans-New_Guinea	Purari	144.97	-7.58	This_study	PASS
Ka27	Purari_Kapuna	Gulf	GULF	Kapuna	PNG_Lowland	Oceania	Trans-New_Guinea	Purari	144.97	-7.58	This_study	PASS
Ka28	Purari_Kapuna	Gulf	GULF	Kapuna	PNG_Lowland	Oceania	Trans-New_Guinea	Purari	144.97	-7.58	This_study	PASS
Ka31	Purari_Kapuna	Gulf	GULF	Kapuna	PNG_Lowland	Oceania	Trans-New_Guinea	Purari	144.97	-7.58	This_study	PASS
Ka32	Purari_Kapuna	Gulf	GULF	Kapuna	PNG_Lowland	Oceania	Trans-New_Guinea	Purari	144.97	-7.58	This_study	PASS
Ka33	Purari_Kapuna	Gulf	GULF	Kapuna	PNG_Lowland	Oceania	Trans-New_Guinea	Purari	144.97	-7.58	This_study	Kin
Ka34	Purari_Kapuna	Gulf	GULF	Kapuna	PNG_Lowland	Oceania	Trans-New_Guinea	Purari	144.97	-7.58	This_study	PASS
Ka35	Purari_Kapuna	Gulf	GULF	Kapuna	PNG_Lowland	Oceania	Trans-New_Guinea	Purari	144.97	-7.58	This_study	PASS
Ka37	Purari_Kapuna	Gulf	GULF	Kapuna	PNG_Lowland	Oceania	Trans-New_Guinea	Purari	144.97	-7.58	This_study	PASS
Ka38	Purari_Kapuna	Gulf	GULF	Kapuna	PNG_Lowland	Oceania	Trans-New_Guinea	Purari	144.97	-7.58	This_study	PASS
Ka39	Purari_Kapuna	Gulf	GULF	Kapuna	PNG_Lowland	Oceania	Trans-New_Guinea	Purari	144.97	-7.58	This_study	PASS
Ka40	Purari_Kapuna	Gulf	GULF	Kapuna	PNG_Lowland	Oceania	Trans-New_Guinea	Purari	144.97	-7.58	This_study	PASS
Ka42	Purari_Kapuna	Gulf	GULF	Kapuna	PNG_Lowland	Oceania	Trans-New_Guinea	Purari	144.97	-7.58	This_study	PASS
Ka43	Purari_Kapuna	Gulf	GULF	Kapuna	PNG_Lowland	Oceania	Trans-New_Guinea	Purari	144.97	-7.58	This_study	PASS
Ka44	Purari_Kapuna	Gulf	GULF	Kapuna	PNG_Lowland	Oceania	Trans-New_Guinea	Purari	144.97	-7.58	This_study	PASS
Ka45	Purari_Kapuna	Gulf	GULF	Kapuna	PNG_Lowland	Oceania	Trans-New_Guinea	Purari	144.97	-7.58	This_study	PASS
Be01	Waima_Bereina	Central	CENTRAL	Bereina	PNG_Lowland	Oceania	Austronesian	Waima	146.51	-8.63	This_study	PASS
Be03	Waima_Bereina	Central	CENTRAL	Bereina	PNG_Lowland	Oceania	Austronesian	Waima	146.51	-8.63	This_study	PASS
Be06	Waima_Bereina	Central	CENTRAL	Bereina	PNG_Lowland	Oceania	Austronesian	Waima	146.51	-8.63	This_study	PASS
Be07	Waima_Bereina	Central	CENTRAL	Bereina	PNG_Lowland	Oceania	Austronesian	Waima	146.51	-8.63	This_study	PASS
Be09	Waima_Bereina	Central	CENTRAL	Bereina	PNG_Lowland	Oceania	Austronesian	Waima	146.51	-8.63	This_study	PASS
Be10	Waima_Bereina	Central	CENTRAL	Bereina	PNG_Lowland	Oceania	Austronesian	Waima	146.51	-8.63	This_study	PASS
Be11	Waima_Bereina	Central	CENTRAL	Bereina	PNG_Lowland	Oceania	Austronesian	Waima	146.51	-8.63	This_study	PASS
Be12	Waima_Bereina	Central	CENTRAL	Bereina	PNG_Lowland	Oceania	Austronesian	Waima	146.51	-8.63	This_study	PASS
Be13	Waima_Bereina	Central	CENTRAL	Bereina	PNG_Lowland	Oceania	Austronesian	Waima	146.51	-8.63	This_study	PASS
Be14	Waima_Bereina	Central	CENTRAL	Bereina	PNG_Lowland	Oceania	Austronesian	Waima	146.51	-8.63	This_study	PASS
Be15	Waima_Bereina	Central	CENTRAL	Bereina	PNG_Lowland	Oceania	Austronesian	Waima	146.51	-8.63	This_study	PASS
Be16	Waima_Bereina	Central	CENTRAL	Bereina	PNG_Lowland	Oceania	Austronesian	Waima	146.51	-8.63	This_study	PASS
Be17	Waima_Bereina	Central	CENTRAL	Bereina	PNG_Lowland	Oceania	Austronesian	Waima	146.51	-8.63	This_study	PASS
Be18	Waima_Bereina	Central	CENTRAL	Bereina	PNG_Lowland	Oceania	Austronesian	Waima	146.51	-8.63	This_study	PASS
Be19	Waima_Bereina	Central	CENTRAL	Bereina	PNG_Lowland	Oceania	Austronesian	Waima	146.51	-8.63	This_study	PASS
Be20	Waima_Bereina	Central	CENTRAL	Bereina	PNG_Lowland	Oceania	Austronesian	Waima	146.51	-8.63	This_study	PASS
Be21	Waima_Bereina	Central	CENTRAL	Bereina	PNG_Lowland	Oceania	Austronesian	Waima	146.51	-8.63	This_study	PASS
Be22	Waima_Bereina	Central	CENTRAL	Bereina	PNG_Lowland	Oceania	Austronesian	Waima	146.51	-8.63	This_study	PASS
Be23	Waima_Bereina	Central	CENTRAL	Bereina	PNG_Lowland	Oceania	Austronesian	Waima	146.51	-8.63	This_study	PASS
Be24	Waima_Bereina	Central	CENTRAL	Bereina	PNG_Lowland	Oceania	Austronesian	Waima	146.51	-8.63	This_study	PASS

Be25	Waima_Bereina	Central	CENTRAL	Bereina	PNG_Lowland	Oceania	Austronesian	Waima	146.51	-8.63	This_study	PASS
Be26	Waima_Bereina	Central	CENTRAL	Bereina	PNG_Lowland	Oceania	Austronesian	Waima	146.51	-8.63	This_study	PASS
Be27	Waima_Bereina	Central	CENTRAL	Bereina	PNG_Lowland	Oceania	Austronesian	Waima	146.51	-8.63	This_study	PASS
Be28	Waima_Bereina	Central	CENTRAL	Bereina	PNG_Lowland	Oceania	Austronesian	Waima	146.51	-8.63	This_study	PASS
Be29	Waima_Bereina	Central	CENTRAL	Bereina	PNG_Lowland	Oceania	Austronesian	Waima	146.51	-8.63	This_study	PASS
Be30	Waima_Bereina	Central	CENTRAL	Bereina	PNG_Lowland	Oceania	Austronesian	Waima	146.51	-8.63	This_study	PASS
Be31	Waima_Bereina	Central	CENTRAL	Bereina	PNG_Lowland	Oceania	Austronesian	Waima	146.51	-8.63	This_study	PASS
Be32	Waima_Bereina	Central	CENTRAL	Bereina	PNG_Lowland	Oceania	Austronesian	Waima	146.51	-8.63	This_study	PASS
Be33	Waima_Bereina	Central	CENTRAL	Bereina	PNG_Lowland	Oceania	Austronesian	Waima	146.51	-8.63	This_study	PASS
Be34	Waima_Bereina	Central	CENTRAL	Bereina	PNG_Lowland	Oceania	Austronesian	Waima	146.51	-8.63	This_study	PASS
papuan6278203	Hula	Central	CENTRAL	Hood_Peninsula	PNG_Lowland	Oceania	Austronesian	Hula	147.72	-10.05	Bergstrom_et_al	PASS
papuan6278314	Hula	Central	CENTRAL	Hood_Peninsula	PNG_Lowland	Oceania	Austronesian	Hula	147.72	-10.05	Bergstrom_et_al	PASS
papuan6278328	Hula	Central	CENTRAL	Hood_Peninsula	PNG_Lowland	Oceania	Austronesian	Hula	147.72	-10.05	Bergstrom_et_al	PC
papuan6278245	Hula	Central	CENTRAL	Hood_Peninsula	PNG_Lowland	Oceania	Austronesian	Hula	147.72	-10.05	Bergstrom_et_al	PASS
papuan6278379	Hula	Central	CENTRAL	Hood_Peninsula	PNG_Lowland	Oceania	Austronesian	Hula	147.72	-10.05	Bergstrom_et_al	PASS
papuan6278322	Hula	Central	CENTRAL	Hood_Peninsula	PNG_Lowland	Oceania	Austronesian	Hula	147.72	-10.05	Bergstrom_et_al	PASS
papuan6278315	Keapara	Central	CENTRAL	Magarida_Palathaona	PNG_Lowland	Oceania	Austronesian	Keapara	148.05	-10.1	Bergstrom_et_al	PASS
papuan6278242	Keapara	Central	CENTRAL	Magarida_Palathaona	PNG_Lowland	Oceania	Austronesian	Keapara	148.05	-10.1	Bergstrom_et_al	PASS
papuan6278227	Keapara	Central	CENTRAL	Magarida_Palathaona	PNG_Lowland	Oceania	Austronesian	Keapara	148.05	-10.1	Bergstrom_et_al	PASS
papuan6278312	Keapara	Central	CENTRAL	Magarida_Palathaona	PNG_Lowland	Oceania	Austronesian	Keapara	148.05	-10.1	Bergstrom_et_al	PASS
papuan6278334	Motu	Central	CENTRAL	Magarida_Lea-lea	PNG_Lowland	Oceania	Austronesian	Motu	147.05	-9.4	Bergstrom_et_al	PASS
papuan6278259	Motu	Central	CENTRAL	Magarida_Lea-lea	PNG_Lowland	Oceania	Austronesian	Motu	147.05	-9.4	Bergstrom_et_al	Imiss
papuan6278212	Motu	Central	CENTRAL	Magarida_Lea-lea	PNG_Lowland	Oceania	Austronesian	Motu	147.05	-9.4	Bergstrom_et_al	PASS
papuan6278324	Motu	Central	CENTRAL	Magarida_Lea-lea	PNG_Lowland	Oceania	Austronesian	Motu	147.05	-9.4	Bergstrom_et_al	PASS
papuan6278213	Motu	Central	CENTRAL	Magarida_Lea-lea	PNG_Lowland	Oceania	Austronesian	Motu	147.05	-9.4	Bergstrom_et_al	PASS
papuan6278211	Motu	Central	CENTRAL	Magarida_Lea-lea	PNG_Lowland	Oceania	Austronesian	Motu	147.05	-9.4	Bergstrom_et_al	PASS
papuan6278360	Motu	Central	CENTRAL	Magarida_Lea-lea	PNG_Lowland	Oceania	Austronesian	Motu	147.05	-9.4	Bergstrom_et_al	PC
papuan6278376	Motu	Central	CENTRAL	Magarida_Lea-lea	PNG_Lowland	Oceania	Austronesian	Motu	147.05	-9.4	Bergstrom_et_al	PASS
papuan6278297	Motu	Central	CENTRAL	Magarida_Lea-lea	PNG_Lowland	Oceania	Austronesian	Motu	147.05	-9.4	Bergstrom_et_al	PASS
papuan6278235	Motu	Central	CENTRAL	Magarida_Lea-lea	PNG_Lowland	Oceania	Austronesian	Motu	147.05	-9.4	Bergstrom_et_al	PASS
papuan6278205	Motu	Central	CENTRAL	Magarida_Lea-lea	PNG_Lowland	Oceania	Austronesian	Motu	147.05	-9.4	Bergstrom_et_al	PASS
papuan6278221	Motu	Central	CENTRAL	Magarida_Lea-lea	PNG_Lowland	Oceania	Austronesian	Motu	147.05	-9.4	Bergstrom_et_al	PASS
papuan6278244	Motu	Central	CENTRAL	Magarida_Lea-lea	PNG_Lowland	Oceania	Austronesian	Motu	147.05	-9.4	Bergstrom_et_al	PASS
papuan6278354	Motu	Central	CENTRAL	Magarida_Lea-lea	PNG_Lowland	Oceania	Austronesian	Motu	147.05	-9.4	Bergstrom_et_al	PASS
papuan6278329	Motu	Central	CENTRAL	Magarida_Lea-lea	PNG_Lowland	Oceania	Austronesian	Motu	147.05	-9.4	Bergstrom_et_al	PASS
papuan6278341	Sinaugoro	Central	CENTRAL	Rigo	PNG_Lowland	Oceania	Austronesian	Sinaugoro	147.9	-9.9	Bergstrom_et_al	PASS
papuan6278302	Sinaugoro	Central	CENTRAL	Rigo	PNG_Lowland	Oceania	Austronesian	Sinaugoro	147.9	-9.9	Bergstrom_et_al	PASS
papuan6278311	Sinaugoro	Central	CENTRAL	Rigo	PNG_Lowland	Oceania	Austronesian	Sinaugoro	147.9	-9.9	Bergstrom_et_al	PASS
papuan6278228	Waima_Magarida	Central	CENTRAL	Magarida	PNG_Lowland	Oceania	Austronesian	Waima	146.5	-8.65	Bergstrom_et_al	PASS
papuan6278331	Waima_Magarida	Central	CENTRAL	Magarida	PNG_Lowland	Oceania	Austronesian	Waima	146.5	-8.65	Bergstrom_et_al	PASS
papuan6278258	Waima_Magarida	Central	CENTRAL	Magarida	PNG_Lowland	Oceania	Austronesian	Waima	146.5	-8.65	Bergstrom_et_al	PASS
papuan6278304	Waima_Magarida	Central	CENTRAL	Magarida	PNG_Lowland	Oceania	Austronesian	Waima	146.5	-8.65	Bergstrom_et_al	PASS
papuan6278069	Waima_Magarida	Central	CENTRAL	Magarida	PNG_Lowland	Oceania	Austronesian	Waima	146.5	-8.65	Bergstrom_et_al	PASS
papuan6278234	Humene	Central	CENTRAL	Magarida_Tubusereia	PNG_Lowland	Oceania	Trans-New_Guinea	Humene	147.5	-9.7	Bergstrom_et_al	PASS
papuan6278446	Mountain_Koiali	Central	CENTRAL	Port_Moresby	PNG_Lowland	Oceania	Trans-New_Guinea	Mountain_Koiali	147.45	-9.05	Bergstrom_et_al	PASS
papuan6278299	Grass_Koiari	Central	CENTRAL	Magarida	PNG_Lowland	Oceania	Trans-New_Guinea	Grass_Koiari	147.45	-9.5	Bergstrom_et_al	PASS
papuan6278349	Grass_Koiari	Central	CENTRAL	Magarida	PNG_Lowland	Oceania	Trans-New_Guinea	Grass_Koiari	147.45	-9.5	Bergstrom_et_al	PASS
papuan6278321	Grass_Koiari	Central	CENTRAL	Magarida	PNG_Lowland	Oceania	Trans-New_Guinea	Grass_Koiari	147.45	-9.5	Bergstrom_et_al	PASS
papuan6278367	Fuyug	Central	CENTRAL	Goilala	PNG_Lowland	Oceania	Trans-New_Guinea	Fuyug	147.25	-8.6	Bergstrom_et_al	PASS
papuan6278342	Fuyug	Central	CENTRAL	Goilala	PNG_Lowland	Oceania	Trans-New_Guinea	Fuyug	147.25	-8.6	Bergstrom_et_al	PASS
papuan6278204	Tauade	Central	CENTRAL	Goilala	PNG_Lowland	Oceania	Trans-New_Guinea	Tauade	147.1	-8.35	Bergstrom_et_al	PASS
papuan6277937	Chuave	Chimbu	CHIMBU	Chuave	PNG_Highland	Oceania	Trans-New_Guinea	Chuave	145.1	-6.2	Bergstrom_et_al	PASS
papuan6277945	Chuave	Chimbu	CHIMBU	Chuave	PNG_Highland	Oceania	Trans-New_Guinea	Chuave	145.1	-6.2	Bergstrom_et_al	PASS

papuan6277969	Chuave	Chimbu	CHIMBU	Chuave	PNG_Highland	Oceania	Trans-New_Guinea	Chuave	145.1	-6.2	Bergstrom_et_al	PASS
papuan6277939	Dadibi	Chimbu	CHIMBU	Karimui	PNG_Highland	Oceania	Trans-New_Guinea	Dadibi	144.6	-6.5	Bergstrom_et_al	PASS
papuan6277953	Golin	Chimbu	CHIMBU	Kundiawa_Minono	PNG_Highland	Oceania	Trans-New_Guinea	Golin	144.85	-6.15	Bergstrom_et_al	PASS
papuan6277915	Golin	Chimbu	CHIMBU	Kundiawa_Minono	PNG_Highland	Oceania	Trans-New_Guinea	Golin	144.85	-6.15	Bergstrom_et_al	PASS
papuan6278046	Golin	Chimbu	CHIMBU	Kundiawa_Minono	PNG_Highland	Oceania	Trans-New_Guinea	Golin	144.85	-6.15	Bergstrom_et_al	PASS
papuan6277989	Golin	Chimbu	CHIMBU	Kundiawa_Minono	PNG_Highland	Oceania	Trans-New_Guinea	Golin	144.85	-6.15	Bergstrom_et_al	PASS
papuan6278006	Golin	Chimbu	CHIMBU	Kundiawa_Minono	PNG_Highland	Oceania	Trans-New_Guinea	Golin	144.85	-6.15	Bergstrom_et_al	PASS
papuan6277931	Golin	Chimbu	CHIMBU	Kundiawa_Minono	PNG_Highland	Oceania	Trans-New_Guinea	Golin	144.85	-6.15	Bergstrom_et_al	PASS
papuan6277888	Kuman	Chimbu	CHIMBU	Kundiawa	PNG_Highland	Oceania	Trans-New_Guinea	Kuman	145	-5.9	Bergstrom_et_al	PASS
papuan6277818	Kuman	Chimbu	CHIMBU	Kundiawa	PNG_Highland	Oceania	Trans-New_Guinea	Kuman	145	-5.9	Bergstrom_et_al	PASS
papuan6277959	Kuman	Chimbu	CHIMBU	Kundiawa	PNG_Highland	Oceania	Trans-New_Guinea	Kuman	145	-5.9	Bergstrom_et_al	PASS
papuan6277943	Kuman	Chimbu	CHIMBU	Kundiawa	PNG_Highland	Oceania	Trans-New_Guinea	Kuman	145	-5.9	Bergstrom_et_al	PASS
papuan6277935	Kuman	Chimbu	CHIMBU	Kundiawa	PNG_Highland	Oceania	Trans-New_Guinea	Kuman	145	-5.9	Bergstrom_et_al	PASS
papuan6277928	Kuman	Chimbu	CHIMBU	Kundiawa	PNG_Highland	Oceania	Trans-New_Guinea	Kuman	145	-5.9	Bergstrom_et_al	PASS
papuan6277956	Kuman	Chimbu	CHIMBU	Kundiawa	PNG_Highland	Oceania	Trans-New_Guinea	Kuman	145	-5.9	Bergstrom_et_al	PASS
papuan6277933	Kuman	Chimbu	CHIMBU	Kundiawa	PNG_Highland	Oceania	Trans-New_Guinea	Kuman	145	-5.9	Bergstrom_et_al	PASS
papuan6277992	Kuman	Chimbu	CHIMBU	Kundiawa	PNG_Highland	Oceania	Trans-New_Guinea	Kuman	145	-5.9	Bergstrom_et_al	PASS
papuan6277930	Kuman	Chimbu	CHIMBU	Kundiawa	PNG_Highland	Oceania	Trans-New_Guinea	Kuman	145	-5.9	Bergstrom_et_al	PASS
papuan6277970	Kuman	Chimbu	CHIMBU	Kundiawa	PNG_Highland	Oceania	Trans-New_Guinea	Kuman	145	-5.9	Bergstrom_et_al	PASS
papuan6277960	Kuman	Chimbu	CHIMBU	Kundiawa	PNG_Highland	Oceania	Trans-New_Guinea	Kuman	145	-5.9	Bergstrom_et_al	PASS
papuan6278019	Kuman	Chimbu	CHIMBU	Kundiawa	PNG_Highland	Oceania	Trans-New_Guinea	Kuman	145	-5.9	Bergstrom_et_al	PASS
papuan6277984	Sinasina	Chimbu	CHIMBU	Kundiawa	PNG_Highland	Oceania	Trans-New_Guinea	Sinasina	145.1	-6.05	Bergstrom_et_al	PASS
papuan6277968	Sinasina	Chimbu	CHIMBU	Kundiawa	PNG_Highland	Oceania	Trans-New_Guinea	Sinasina	145.1	-6.05	Bergstrom_et_al	PASS
papuan6277921	Sinasina	Chimbu	CHIMBU	Kundiawa	PNG_Highland	Oceania	Trans-New_Guinea	Sinasina	145.1	-6.05	Bergstrom_et_al	PASS
papuan6277977	Sinasina	Chimbu	CHIMBU	Kundiawa	PNG_Highland	Oceania	Trans-New_Guinea	Sinasina	145.1	-6.05	Bergstrom_et_al	PASS
papuan6277938	Sinasina	Chimbu	CHIMBU	Kundiawa	PNG_Highland	Oceania	Trans-New_Guinea	Sinasina	145.1	-6.05	Bergstrom_et_al	PASS
papuan6277996	Sinasina	Chimbu	CHIMBU	Kundiawa	PNG_Highland	Oceania	Trans-New_Guinea	Sinasina	145.1	-6.05	Bergstrom_et_al	PASS
papuan6278004	Sinasina	Chimbu	CHIMBU	Kundiawa	PNG_Highland	Oceania	Trans-New_Guinea	Sinasina	145.1	-6.05	Bergstrom_et_al	PASS
papuan6278062	Sinasina	Chimbu	CHIMBU	Kundiawa	PNG_Highland	Oceania	Trans-New_Guinea	Sinasina	145.1	-6.05	Bergstrom_et_al	PASS
papuan6278014	Sinasina	Chimbu	CHIMBU	Kundiawa	PNG_Highland	Oceania	Trans-New_Guinea	Sinasina	145.1	-6.05	Bergstrom_et_al	PASS
papuan6278524	Sinasina	Chimbu	CHIMBU	Kundiawa	PNG_Highland	Oceania	Trans-New_Guinea	Sinasina	145.1	-6.05	Bergstrom_et_al	PASS
papuan6278332	Kuanua	East_New_Britain	EAST_NEW_BRITAIN	Rabaul	Bismarck_Arch	Oceania	Austronesian	Kuanua	152.2	-4.35	Bergstrom_et_al	PASS
papuan6278210	Kuanua	East_New_Britain	EAST_NEW_BRITAIN	Rabaul	Bismarck_Arch	Oceania	Austronesian	Kuanua	152.2	-4.35	Bergstrom_et_al	PASS
papuan6278269	Kuanua	East_New_Britain	EAST_NEW_BRITAIN	Rabaul	Bismarck_Arch	Oceania	Austronesian	Kuanua	152.2	-4.35	Bergstrom_et_al	PASS
papuan6278306	MIXED	MIXED	EAST_NEW_BRITAIN	MIXED	Bismarck_Arch	Oceania	MIXED	MIXED	NA	NA	Bergstrom_et_al	Mixed
papuan6278343	Kairiru	East_Sepik	EAST_SEPIK	Wewak	PNG_Lowland	Oceania	Austronesian	Kairiru	143.65	-3.6	Bergstrom_et_al	PASS
papuan6278035	Ambulas	East_Sepik	EAST_SEPIK	Wewak	PNG_Lowland	Oceania	Lower_Sepik-Ramu	Ambulas	143.1	-3.75	Bergstrom_et_al	PASS
papuan6278219	Ambulas	East_Sepik	EAST_SEPIK	Wewak	PNG_Lowland	Oceania	Lower_Sepik-Ramu	Ambulas	143.1	-3.75	Bergstrom_et_al	PASS
papuan6278587	Angoram	East_Sepik	EAST_SEPIK	Wewak_Yamen	PNG_Lowland	Oceania	Lower_Sepik-Ramu	Angoram	144.05	-4.1	Bergstrom_et_al	PASS
papuan6278294	Angoram	East_Sepik	EAST_SEPIK	Wewak_Yamen	PNG_Lowland	Oceania	Lower_Sepik-Ramu	Angoram	144.05	-4.1	Bergstrom_et_al	PASS
papuan6278012	Boikin	East_Sepik	EAST_SEPIK	Yangoru	PNG_Lowland	Oceania	Lower_Sepik-Ramu	Boikin	143.3	-3.23	Bergstrom_et_al	PASS
papuan6278021	Boikin	East_Sepik	EAST_SEPIK	Yangoru	PNG_Lowland	Oceania	Lower_Sepik-Ramu	Boikin	143.5	-3.65	Bergstrom_et_al	PASS
papuan6278289	Boikin	East_Sepik	EAST_SEPIK	Yangoru	PNG_Lowland	Oceania	Lower_Sepik-Ramu	Boikin	143.5	-3.65	Bergstrom_et_al	PASS
papuan6278083	Boikin	East_Sepik	EAST_SEPIK	Yangoru	PNG_Lowland	Oceania	Lower_Sepik-Ramu	Boikin	143.5	-3.65	Bergstrom_et_al	PASS
papuan6278491	MIXED	MIXED	EAST_SEPIK	MIXED	PNG_Lowland	Oceania	MIXED	MIXED	NA	NA	Bergstrom_et_al	Mixed
papuan6278470	MIXED	MIXED	EAST_SEPIK	MIXED	PNG_Lowland	Oceania	MIXED	MIXED	NA	NA	Bergstrom_et_al	Mixed
papuan6278555	Alekano	Eastern_Highlands	EASTERN_HIGHLANDS	Goroka_Kifamu_Mission	PNG_Highland	Oceania	Trans-New_Guinea	Alekano	145.35	-6.05	Bergstrom_et_al	PASS
papuan6278523	Alekano	Eastern_Highlands	EASTERN_HIGHLANDS	Goroka_Kifamu_Mission	PNG_Highland	Oceania	Trans-New_Guinea	Alekano	145.35	-6.05	Bergstrom_et_al	PASS
papuan6278540	Alekano	Eastern_Highlands	EASTERN_HIGHLANDS	Goroka_Kifamu_Mission	PNG_Highland	Oceania	Trans-New_Guinea	Alekano	145.35	-6.05	Bergstrom_et_al	PASS
papuan6278288	Alekano	Eastern_Highlands	EASTERN_HIGHLANDS	Goroka_Kifamu_Mission	PNG_Highland	Oceania	Trans-New_Guinea	Alekano	145.35	-6.05	Bergstrom_et_al	PASS
papuan6278295	Awiyaana	Eastern_Highlands	EASTERN_HIGHLANDS	Okapa	PNG_Highland	Oceania	Trans-New_Guinea	Awiyaana	145.8	-6.6	Bergstrom_et_al	PASS
papuan6278253	Awiyaana	Eastern_Highlands	EASTERN_HIGHLANDS	Okapa	PNG_Highland	Oceania	Trans-New_Guinea	Awiyaana	145.8	-6.6	Bergstrom_et_al	PASS
papuan6278363	Awiyaana	Eastern_Highlands	EASTERN_HIGHLANDS	Okapa	PNG_Highland	Oceania	Trans-New_Guinea	Awiyaana	145.8	-6.6	Bergstrom_et_al	PASS

papuan6278386	Awiyaana	Eastern_Highlands	EASTERN_HIGHLANDS	Okapa	PNG_Highland	Oceania	Trans-New_Guinea	Awiyaana	145.8	-6.6	Bergstrom_et_al	PASS
papuan6278283	Awiyaana	Eastern_Highlands	EASTERN_HIGHLANDS	Okapa	PNG_Highland	Oceania	Trans-New_Guinea	Awiyaana	145.8	-6.6	Bergstrom_et_al	PASS
papuan6278532	Benabena	Eastern_Highlands	EASTERN_HIGHLANDS	Goroka	PNG_Highland	Oceania	Trans-New_Guinea	Benabena	145.5	-6.1	Bergstrom_et_al	PASS
papuan6278601	Kamano	Eastern_Highlands	EASTERN_HIGHLANDS	Okapa	PNG_Highland	Oceania	Trans-New_Guinea	Kamano	145.7	-6.25	Bergstrom_et_al	PASS
papuan6278586	Kamano	Eastern_Highlands	EASTERN_HIGHLANDS	Okapa	PNG_Highland	Oceania	Trans-New_Guinea	Kamano	145.7	-6.25	Bergstrom_et_al	PASS
papuan6278563	Siane	Eastern_Highlands	EASTERN_HIGHLANDS	Goroka_Watabung	PNG_Highland	Oceania	Trans-New_Guinea	Siane	145.2	-6.1	Bergstrom_et_al	PASS
papuan6278539	Siane	Eastern_Highlands	EASTERN_HIGHLANDS	Goroka_Watabung	PNG_Highland	Oceania	Trans-New_Guinea	Siane	145.2	-6.1	Bergstrom_et_al	PASS
papuan6278492	Siane	Eastern_Highlands	EASTERN_HIGHLANDS	Goroka_Watabung	PNG_Highland	Oceania	Trans-New_Guinea	Siane	145.2	-6.1	Bergstrom_et_al	PASS
papuan6278556	Siane	Eastern_Highlands	EASTERN_HIGHLANDS	Goroka_Watabung	PNG_Highland	Oceania	Trans-New_Guinea	Siane	145.2	-6.1	Bergstrom_et_al	PASS
papuan6278338	Simbari	Eastern_Highlands	EASTERN_HIGHLANDS	Marawaka	PNG_Highland	Oceania	Trans-New_Guinea	Simbari	145.6	-7.05	Bergstrom_et_al	PASS
papuan6277940	Tokano	Eastern_Highlands	EASTERN_HIGHLANDS	Goroka_Watabung	PNG_Highland	Oceania	Trans-New_Guinea	Tokano	145.25	-6.05	Bergstrom_et_al	PASS
papuan6278507	Yagaria	Eastern_Highlands	EASTERN_HIGHLANDS	Goroka	PNG_Highland	Oceania	Trans-New_Guinea	Yagaria	145.3	-6.25	Bergstrom_et_al	PASS
papuan6278516	Yagaria	Eastern_Highlands	EASTERN_HIGHLANDS	Goroka	PNG_Highland	Oceania	Trans-New_Guinea	Yagaria	145.3	-6.25	Bergstrom_et_al	PASS
papuan6278515	Yaweyuha	Eastern_Highlands	EASTERN_HIGHLANDS	Goroka	PNG_Highland	Oceania	Trans-New_Guinea	Yaweyuha	145.3	-6.15	Bergstrom_et_al	PASS
papuan6278224	Yaweyuha	Eastern_Highlands	EASTERN_HIGHLANDS	Goroka	PNG_Highland	Oceania	Trans-New_Guinea	Yaweyuha	145.3	-6.15	Bergstrom_et_al	PASS
papuan6278166	Yipma	Eastern_Highlands	EASTERN_HIGHLANDS	Marawaka	PNG_Highland	Oceania	Trans-New_Guinea	Yipma	145.8	-6.9	Bergstrom_et_al	PASS
papuan6278174	Yipma	Eastern_Highlands	EASTERN_HIGHLANDS	Marawaka	PNG_Highland	Oceania	Trans-New_Guinea	Yipma	145.8	-6.9	Bergstrom_et_al	PASS
papuan6278134	Yipma	Eastern_Highlands	EASTERN_HIGHLANDS	Marawaka	PNG_Highland	Oceania	Trans-New_Guinea	Yipma	145.8	-6.9	Bergstrom_et_al	PASS
papuan6278126	Yipma	Eastern_Highlands	EASTERN_HIGHLANDS	Marawaka	PNG_Highland	Oceania	Trans-New_Guinea	Yipma	145.8	-6.9	Bergstrom_et_al	PASS
papuan6278020	Enga	Enga	ENGA	Wabag	PNG_Highland	Oceania	Trans-New_Guinea	Enga	143.6	-5.5	Bergstrom_et_al	PASS
papuan6278028	Enga	Enga	ENGA	Wabag	PNG_Highland	Oceania	Trans-New_Guinea	Enga	143.6	-5.5	Bergstrom_et_al	PASS
papuan6278042	Enga	Enga	ENGA	Wabag	PNG_Highland	Oceania	Trans-New_Guinea	Enga	143.6	-5.5	Bergstrom_et_al	PASS
papuan6278111	Enga	Enga	ENGA	Wabag	PNG_Highland	Oceania	Trans-New_Guinea	Enga	143.6	-5.5	Bergstrom_et_al	PASS
papuan6278128	Enga	Enga	ENGA	Wabag	PNG_Highland	Oceania	Trans-New_Guinea	Enga	143.6	-5.5	Bergstrom_et_al	PASS
papuan6277902	Enga	Enga	ENGA	Wabag	PNG_Highland	Oceania	Trans-New_Guinea	Enga	143.6	-5.5	Bergstrom_et_al	PASS
papuan6278027	Enga	Enga	ENGA	Wabag	PNG_Highland	Oceania	Trans-New_Guinea	Enga	143.6	-5.5	Bergstrom_et_al	PASS
papuan6278011	Enga	Enga	ENGA	Wabag	PNG_Highland	Oceania	Trans-New_Guinea	Enga	143.6	-5.5	Bergstrom_et_al	PASS
papuan6278382	Enga	Enga	ENGA	Wabag	PNG_Highland	Oceania	Trans-New_Guinea	Enga	143.6	-5.5	Bergstrom_et_al	PASS
papuan6278430	Enga	Enga	ENGA	Wabag	PNG_Highland	Oceania	Trans-New_Guinea	Enga	143.6	-5.5	Bergstrom_et_al	PASS
papuan6278158	Akoye	Gulf	GULF	Kaberofe	PNG_Lowland	Oceania	Trans-New_Guinea	Akoye	145.7	-7.65	Bergstrom_et_al	PASS
papuan6278150	Akoye	Gulf	GULF	Kaberofe	PNG_Lowland	Oceania	Trans-New_Guinea	Akoye	145.7	-7.65	Bergstrom_et_al	PASS
papuan6278142	Akoye	Gulf	GULF	Kaberofe	PNG_Lowland	Oceania	Trans-New_Guinea	Akoye	145.7	-7.65	Bergstrom_et_al	PASS
papuan6278220	Ikobi	Gulf	GULF	Kerema	PNG_Lowland	Oceania	Trans-New_Guinea	Ikobi	143.5	-7.1	Bergstrom_et_al	PASS
papuan6278414	MIXED	MIXED	GULF	MIXED	PNG_Lowland	Oceania	MIXED	MIXED	NA	NA	Bergstrom_et_al	Mixed
papuan6278390	MIXED	MIXED	GULF	MIXED	PNG_Lowland	Oceania	MIXED	MIXED	NA	NA	Bergstrom_et_al	Mixed
papuan6278317	MIXED	MIXED	GULF	MIXED	PNG_Lowland	Oceania	MIXED	MIXED	NA	NA	Bergstrom_et_al	Mixed
papuan6278292	MIXED	MIXED	GULF	MIXED	PNG_Lowland	Oceania	MIXED	MIXED	NA	NA	Bergstrom_et_al	Mixed
papuan6278366	Orokolo	Gulf	GULF	Kerema	PNG_Lowland	Oceania	Trans-New_Guinea	Orokolo	145.3	-7.6	Bergstrom_et_al	PASS
papuan6278236	Orokolo	Gulf	GULF	Kerema	PNG_Lowland	Oceania	Trans-New_Guinea	Orokolo	145.3	-7.6	Bergstrom_et_al	PASS
papuan6278387	Orokolo	Gulf	GULF	Kerema	PNG_Lowland	Oceania	Trans-New_Guinea	Orokolo	145.3	-7.6	Bergstrom_et_al	PASS
papuan6278347	Orokolo	Gulf	GULF	Kerema	PNG_Lowland	Oceania	Trans-New_Guinea	Orokolo	145.3	-7.6	Bergstrom_et_al	PASS
papuan6278381	Orokolo	Gulf	GULF	Kerema	PNG_Lowland	Oceania	Trans-New_Guinea	Orokolo	145.3	-7.6	Bergstrom_et_al	PASS
papuan6278274	Purari_Purari	Gulf	GULF	Puari	PNG_Lowland	Oceania	Trans-New_Guinea	Purari	145	-7.6	Bergstrom_et_al	PASS
papuan6278251	Tairuma	Gulf	GULF	Kerema	PNG_Lowland	Oceania	Trans-New_Guinea	Tairuma	145.8	-7.95	Bergstrom_et_al	PASS
papuan6278327	Tairuma	Gulf	GULF	Kerema	PNG_Lowland	Oceania	Trans-New_Guinea	Tairuma	145.8	-7.95	Bergstrom_et_al	PASS
papuan6278243	Tairuma	Gulf	GULF	Kerema	PNG_Lowland	Oceania	Trans-New_Guinea	Tairuma	145.8	-7.95	Bergstrom_et_al	PASS
papuan6278319	Tairuma	Gulf	GULF	Kerema	PNG_Lowland	Oceania	Trans-New_Guinea	Tairuma	145.8	-7.95	Bergstrom_et_al	PASS
papuan6278303	Tairuma	Gulf	GULF	Kerema	PNG_Lowland	Oceania	Trans-New_Guinea	Tairuma	145.8	-7.95	Bergstrom_et_al	PASS
papuan6278316	Tairuma	Gulf	GULF	Kerema	PNG_Lowland	Oceania	Trans-New_Guinea	Tairuma	145.8	-7.95	Bergstrom_et_al	PASS
papuan6278300	Toaripi	Gulf	GULF	Kerema	PNG_Lowland	Oceania	Trans-New_Guinea	Toaripi	146.2	-8.1	Bergstrom_et_al	PASS
papuan6278260	Toaripi	Gulf	GULF	Kerema	PNG_Lowland	Oceania	Trans-New_Guinea	Toaripi	146.2	-8.1	Bergstrom_et_al	PASS
papuan6278398	Toaripi	Gulf	GULF	Kerema	PNG_Lowland	Oceania	Trans-New_Guinea	Toaripi	146.2	-8.1	Bergstrom_et_al	PASS
papuan6278229	Toaripi	Gulf	GULF	Kerema	PNG_Lowland	Oceania	Trans-New_Guinea	Toaripi	146.2	-8.1	Bergstrom_et_al	PASS

papuan6278368	Toaripi	Gulf	GULF	Kerema	PNG_Lowland	Oceania	Trans-New_Guinea	Toaripi	146.2	-8.1	Bergstrom_et_al	PASS
papuan6278369	Toaripi	Gulf	GULF	Kerema	PNG_Lowland	Oceania	Trans-New_Guinea	Toaripi	146.2	-8.1	Bergstrom_et_al	PASS
papuan6278374	Toaripi	Gulf	GULF	Kerema	PNG_Lowland	Oceania	Trans-New_Guinea	Toaripi	146.2	-8.1	Bergstrom_et_al	PASS
papuan6278546	Takia	Madang_Lowland	MADANG	Badilu	PNG_Lowland	Oceania	Austronesian	Takia	146	-4.7	Bergstrom_et_al	PASS
papuan6277971	Aruamu	Madang_Lowland	MADANG	Bogia	PNG_Lowland	Oceania	Lower_Sepik-Ramu	Aruamu	144.85	-4.3	Bergstrom_et_al	PASS
papuan6278603	Giri	Madang_Lowland	MADANG	Garati	PNG_Lowland	Oceania	Lower_Sepik-Ramu	Giri	144.7	-4.25	Bergstrom_et_al	PASS
papuan6278596	Kominimung	Madang_Lowland	MADANG	Madang	PNG_Lowland	Oceania	Lower_Sepik-Ramu	Kominimung	144.75	-4.7	Bergstrom_et_al	PASS
papuan6278525	Rao	Madang_Lowland	MADANG	Madang	PNG_Lowland	Oceania	Lower_Sepik-Ramu	Rao	144.5	-4.8	Bergstrom_et_al	PASS
papuan6278569	Rao	Madang_Lowland	MADANG	Madang	PNG_Lowland	Oceania	Lower_Sepik-Ramu	Rao	144.5	-4.8	Bergstrom_et_al	PASS
papuan6278593	MIXED	MIXED	MADANG	MIXED	PNG_Lowland	Oceania	MIXED	MIXED	NA	NA	Bergstrom_et_al	Mixed
papuan6278529	MIXED	MIXED	MADANG	MIXED	PNG_Lowland	Oceania	MIXED	MIXED	NA	NA	Bergstrom_et_al	Mixed
papuan6278565	MIXED	MIXED	MADANG	MIXED	PNG_Lowland	Oceania	MIXED	MIXED	NA	NA	Bergstrom_et_al	Mixed
papuan6278583	MIXED	MIXED	MADANG	MIXED	PNG_Lowland	Oceania	MIXED	MIXED	NA	NA	Bergstrom_et_al	Mixed
papuan6278561	MIXED	MIXED	MADANG	MIXED	PNG_Lowland	Oceania	MIXED	MIXED	NA	NA	Bergstrom_et_al	Mixed
papuan6278584	MIXED	MIXED	MADANG	MIXED	PNG_Lowland	Oceania	MIXED	MIXED	NA	NA	Bergstrom_et_al	Mixed
papuan6278557	Amaimon	Madang_Lowland	MADANG	Madang	PNG_Lowland	Oceania	Trans-New_Guinea	Amaimon	145.37	-5.2	Bergstrom_et_al	PASS
papuan6277941	Amele	Madang_Lowland	MADANG	Utu_Mission	PNG_Lowland	Oceania	Trans-New_Guinea	Amele	145.7	-5.25	Bergstrom_et_al	PASS
papuan6278592	Amele	Madang_Lowland	MADANG	Utu_Mission	PNG_Lowland	Oceania	Trans-New_Guinea	Amele	145.7	-5.25	Bergstrom_et_al	PASS
papuan6278553	Bemal	Madang_Lowland	MADANG	Trans-Gogol	PNG_Lowland	Oceania	Trans-New_Guinea	Bemal	145.45	-5.3	Bergstrom_et_al	PASS
papuan6277872	Biyom	Madang_Highland	MADANG	Brahmin	PNG_Highland	Oceania	Trans-New_Guinea	Biyom	145.45	-5.85	Bergstrom_et_al	PASS
papuan6277918	Gende	Madang_Highland	MADANG	Bundi	PNG_Highland	Oceania	Trans-New_Guinea	Gende	145.15	-5.7	Bergstrom_et_al	PASS
papuan6277910	Gende	Madang_Highland	MADANG	Bundi	PNG_Highland	Oceania	Trans-New_Guinea	Gende	145.15	-5.7	Bergstrom_et_al	PASS
papuan6277885	Gende	Madang_Highland	MADANG	Bundi	PNG_Highland	Oceania	Trans-New_Guinea	Gende	145.15	-5.7	Bergstrom_et_al	PASS
papuan6277906	Gende	Madang_Highland	MADANG	Bundi	PNG_Highland	Oceania	Trans-New_Guinea	Gende	145.15	-5.7	Bergstrom_et_al	PASS
papuan6277897	Gende	Madang_Highland	MADANG	Bundi	PNG_Highland	Oceania	Trans-New_Guinea	Gende	145.15	-5.7	Bergstrom_et_al	PASS
papuan6277881	Gende	Madang_Highland	MADANG	Bundi	PNG_Highland	Oceania	Trans-New_Guinea	Gende	145.15	-5.7	Bergstrom_et_al	PASS
papuan6277873	Gende	Madang_Highland	MADANG	Bundi	PNG_Highland	Oceania	Trans-New_Guinea	Gende	145.15	-5.7	Bergstrom_et_al	PASS
papuan6277967	Gende	Madang_Highland	MADANG	Bundi	PNG_Highland	Oceania	Trans-New_Guinea	Gende	145.15	-5.7	Bergstrom_et_al	PASS
papuan6277868	Gende	Madang_Highland	MADANG	Bundi	PNG_Highland	Oceania	Trans-New_Guinea	Gende	145.15	-5.7	Bergstrom_et_al	PASS
papuan6277883	Gende	Madang_Highland	MADANG	Bundi	PNG_Highland	Oceania	Trans-New_Guinea	Gende	145.15	-5.7	Bergstrom_et_al	PASS
papuan6277934	Gende	Madang_Highland	MADANG	Bundi	PNG_Highland	Oceania	Trans-New_Guinea	Gende	145.15	-5.7	Bergstrom_et_al	PASS
papuan6277907	Gende	Madang_Highland	MADANG	Bundi	PNG_Highland	Oceania	Trans-New_Guinea	Gende	145.15	-5.7	Bergstrom_et_al	PASS
papuan6277849	Gende	Madang_Highland	MADANG	Bundi	PNG_Highland	Oceania	Trans-New_Guinea	Gende	145.15	-5.7	Bergstrom_et_al	PASS
papuan6277911	Gende	Madang_Highland	MADANG	Bundi	PNG_Highland	Oceania	Trans-New_Guinea	Gende	145.15	-5.7	Bergstrom_et_al	PASS
papuan6277889	Gende	Madang_Highland	MADANG	Bundi	PNG_Highland	Oceania	Trans-New_Guinea	Gende	145.15	-5.7	Bergstrom_et_al	PASS (2nd degree Kinship)
papuan6277899	Gende	Madang_Highland	MADANG	Bundi	PNG_Highland	Oceania	Trans-New_Guinea	Gende	145.15	-5.7	Bergstrom_et_al	PASS
papuan6277866	Gende	Madang_Highland	MADANG	Bundi	PNG_Highland	Oceania	Trans-New_Guinea	Gende	145.15	-5.7	Bergstrom_et_al	PASS
papuan6277890	Gende	Madang_Highland	MADANG	Bundi	PNG_Highland	Oceania	Trans-New_Guinea	Gende	145.15	-5.7	Bergstrom_et_al	PASS
papuan6277898	Gende	Madang_Highland	MADANG	Bundi	PNG_Highland	Oceania	Trans-New_Guinea	Gende	145.15	-5.7	Bergstrom_et_al	PASS
papuan6277859	Gende	Madang_Highland	MADANG	Bundi	PNG_Highland	Oceania	Trans-New_Guinea	Gende	145.15	-5.7	Bergstrom_et_al	PASS
papuan6277827	Gende	Madang_Highland	MADANG	Bundi	PNG_Highland	Oceania	Trans-New_Guinea	Gende	145.15	-5.7	Bergstrom_et_al	PASS
papuan6277820	Gende	Madang_Highland	MADANG	Bundi	PNG_Highland	Oceania	Trans-New_Guinea	Gende	145.15	-5.7	Bergstrom_et_al	Kin
papuan6277836	Gende	Madang_Highland	MADANG	Bundi	PNG_Highland	Oceania	Trans-New_Guinea	Gende	145.15	-5.7	Bergstrom_et_al	PASS
papuan6277844	Gende	Madang_Highland	MADANG	Bundi	PNG_Highland	Oceania	Trans-New_Guinea	Gende	145.15	-5.7	Bergstrom_et_al	PASS
papuan6277852	Gende	Madang_Highland	MADANG	Bundi	PNG_Highland	Oceania	Trans-New_Guinea	Gende	145.15	-5.7	Bergstrom_et_al	PASS
papuan6277884	Gende	Madang_Highland	MADANG	Bundi	PNG_Highland	Oceania	Trans-New_Guinea	Gende	145.15	-5.7	Bergstrom_et_al	PASS
papuan6277876	Gende	Madang_Highland	MADANG	Bundi	PNG_Highland	Oceania	Trans-New_Guinea	Gende	145.15	-5.7	Bergstrom_et_al	PASS
papuan6277864	Gende	Madang_Highland	MADANG	Bundi	PNG_Highland	Oceania	Trans-New_Guinea	Gende	145.15	-5.7	Bergstrom_et_al	PASS
papuan6277905	Gende	Madang_Highland	MADANG	Bundi	PNG_Highland	Oceania	Trans-New_Guinea	Gende	145.15	-5.7	Bergstrom_et_al	PASS
papuan6277817	Gende	Madang_Highland	MADANG	Bundi	PNG_Highland	Oceania	Trans-New_Guinea	Gende	145.15	-5.7	Bergstrom_et_al	PASS
papuan6277874	Gende	Madang_Highland	MADANG	Bundi	PNG_Highland	Oceania	Trans-New_Guinea	Gende	145.15	-5.7	Bergstrom_et_al	PASS
papuan6277882	Gende	Madang_Highland	MADANG	Bundi	PNG_Highland	Oceania	Trans-New_Guinea	Gende	145.15	-5.7	Bergstrom_et_al	PASS
papuan6277919	Gende	Madang_Highland	MADANG	Bundi	PNG_Highland	Oceania	Trans-New_Guinea	Gende	145.15	-5.7	Bergstrom_et_al	Kin

papuan6277974	Gende	Madang_Highland	MADANG	Bundi	PNG_Highland	Oceania	Trans-New_Guinea	Gende	145.15	-5.7	Bergstrom_et_al	PASS
papuan6277966	Gende	Madang_Highland	MADANG	Bundi	PNG_Highland	Oceania	Trans-New_Guinea	Gende	145.15	-5.7	Bergstrom_et_al	Kin
papuan6277950	Gende	Madang_Highland	MADANG	Bundi	PNG_Highland	Oceania	Trans-New_Guinea	Gende	145.15	-5.7	Bergstrom_et_al	PASS
papuan6277999	Gende	Madang_Highland	MADANG	Bundi	PNG_Highland	Oceania	Trans-New_Guinea	Gende	145.15	-5.7	Bergstrom_et_al	PASS
papuan6277991	Gende	Madang_Highland	MADANG	Bundi	PNG_Highland	Oceania	Trans-New_Guinea	Gende	145.15	-5.7	Bergstrom_et_al	PASS
papuan6277826	Gende	Madang_Highland	MADANG	Bundi	PNG_Highland	Oceania	Trans-New_Guinea	Gende	145.15	-5.7	Bergstrom_et_al	PASS
papuan6277828	Gende	Madang_Highland	MADANG	Bundi	PNG_Highland	Oceania	Trans-New_Guinea	Gende	145.15	-5.7	Bergstrom_et_al	PASS
papuan6277819	Gende	Madang_Highland	MADANG	Bundi	PNG_Highland	Oceania	Trans-New_Guinea	Gende	145.15	-5.7	Bergstrom_et_al	PASS
papuan6277891	Gende	Madang_Highland	MADANG	Bundi	PNG_Highland	Oceania	Trans-New_Guinea	Gende	145.15	-5.7	Bergstrom_et_al	PASS
papuan6277829	Gende	Madang_Highland	MADANG	Bundi	PNG_Highland	Oceania	Trans-New_Guinea	Gende	145.15	-5.7	Bergstrom_et_al	PASS
papuan6278607	Gende	Madang_Highland	MADANG	Bundi	PNG_Highland	Oceania	Trans-New_Guinea	Gende	145.15	-5.7	Bergstrom_et_al	PASS (2nd degree Kinship)
papuan6278541	MIXED	Madang_Lowland	MADANG	MIXED	PNG_Lowland	Oceania	Trans-New_Guinea	MIXED	145.65	-5.2	Bergstrom_et_al	PASS
papuan6278577	Korak	Madang_Lowland	MADANG	Rempi_Mission	PNG_Lowland	Oceania	Trans-New_Guinea	Korak	145.45	-4.55	Bergstrom_et_al	PASS
papuan6278533	Nobonob	Madang_Lowland	MADANG	Rempi_Mission	PNG_Lowland	Oceania	Trans-New_Guinea	Nobonob	145.7	-5.1	Bergstrom_et_al	PASS
papuan6278509	Nobonob	Madang_Lowland	MADANG	Rempi_Mission	PNG_Lowland	Oceania	Trans-New_Guinea	Nobonob	145.7	-5.1	Bergstrom_et_al	PASS
papuan6278597	MIXED	Madang_Lowland	MADANG	MIXED	PNG_Lowland	Oceania	Trans-New_Guinea	MIXED	NA	NA	Bergstrom_et_al	PASS
papuan6278582	Pondoma	Madang_Lowland	MADANG	Josephstaal	PNG_Lowland	Oceania	Trans-New_Guinea	Pondoma	145	-4.75	Bergstrom_et_al	PASS
papuan6278506	MIXED	Madang_Lowland	MADANG	MIXED	PNG_Lowland	Oceania	Trans-New_Guinea	MIXED	NA	NA	Bergstrom_et_al	PASS
papuan6278606	Sop	Madang_Lowland	MADANG	Madang	PNG_Lowland	Oceania	Trans-New_Guinea	Sop	145.45	-5.5	Bergstrom_et_al	PASS
papuan6278564	Wagi	Madang_Lowland	MADANG	Rempi_Mission	PNG_Lowland	Oceania	Trans-New_Guinea	Wagi	145.75	-5.2	Bergstrom_et_al	PASS
papuan6278572	Wagi	Madang_Lowland	MADANG	Rempi_Mission	PNG_Lowland	Oceania	Trans-New_Guinea	Wagi	145.75	-5.2	Bergstrom_et_al	PASS
papuan6278580	Wagi	Madang_Lowland	MADANG	Rempi_Mission	PNG_Lowland	Oceania	Trans-New_Guinea	Wagi	145.75	-5.2	Bergstrom_et_al	PASS
papuan6278493	Wagi	Madang_Lowland	MADANG	Rempi_Mission	PNG_Lowland	Oceania	Trans-New_Guinea	Wagi	145.75	-5.2	Bergstrom_et_al	PASS
papuan6278501	Wagi	Madang_Lowland	MADANG	Rempi_Mission	PNG_Lowland	Oceania	Trans-New_Guinea	Wagi	145.75	-5.2	Bergstrom_et_al	PASS
papuan6278589	Wagi	Madang_Lowland	MADANG	Rempi_Mission	PNG_Lowland	Oceania	Trans-New_Guinea	Wagi	145.75	-5.2	Bergstrom_et_al	PASS (2nd degree Kinship)
papuan6278273	Tulu-Bohuai	Manus_New_Ireland	MANUS	Lorengau	Bismarck_Arch	Oceania	Austronesian	Tulu-Bohuai	146.85	-2.1	Bergstrom_et_al	PASS
papuan6278301	MIXED	MIXED	MANUS	MIXED	Bismarck_Arch	Oceania	MIXED	MIXED	NA	NA	Bergstrom_et_al	Mixed
papuan6278320	MIXED	MIXED	MANUS	MIXED	Bismarck_Arch	Oceania	MIXED	MIXED	NA	NA	Bergstrom_et_al	Mixed
papuan6278513	MIXED	Misima	MILNE_BAY	MIXED	Massim	Oceania	Austronesian	MIXED	NA	NA	Bergstrom_et_al	PASS
papuan6278277	Dobu	Fergusson	MILNE_BAY	Esaala	Massim	Oceania	Austronesian	Dobu	151.26	-9.95	Bergstrom_et_al	PASS
papuan6278358	MIXED	MIXED	MILNE_BAY	MIXED	Massim	Oceania	MIXED	MIXED	NA	NA	Bergstrom_et_al	Mixed
papuan6278252	MIXED	MIXED	MILNE_BAY	MIXED	Massim	Oceania	MIXED	MIXED	NA	NA	Bergstrom_et_al	Mixed
papuan6278378	MIXED	MIXED	MILNE_BAY	MIXED	Massim	Oceania	MIXED	MIXED	NA	NA	Bergstrom_et_al	Mixed
papuan6278052	Umanakaina	Mainland_Eastern_Tip	MILNE_BAY	Rabaraba	Massim	Oceania	Trans-New_Guinea	Umanakaina	149.6	-9.9	Bergstrom_et_al	PASS
papuan6278508	MIXED	MIXED	MIXED	MIXED	MIXED	Oceania	Trans-New_Guinea	MIXED	NA	NA	Bergstrom_et_al	Mixed
papuan6277870	MIXED	MIXED	MIXED	MIXED	MIXED	Oceania	Trans-New_Guinea	MIXED	NA	NA	Bergstrom_et_al	Mixed
papuan6278517	MIXED	MIXED	MIXED	MIXED	MIXED	Oceania	Austronesian	MIXED	NA	NA	Bergstrom_et_al	Mixed
papuan6278514	MIXED	MIXED	MIXED	MIXED	MIXED	Oceania	Trans-New_Guinea	MIXED	NA	NA	Bergstrom_et_al	Mixed
papuan6278591	MIXED	MIXED	MIXED	MIXED	MIXED	Oceania	MIXED	MIXED	NA	NA	Bergstrom_et_al	Mixed
papuan6278498	MIXED	MIXED	MIXED	MIXED	MIXED	Oceania	MIXED	MIXED	NA	NA	Bergstrom_et_al	Mixed
papuan6278537	MIXED	MIXED	MIXED	MIXED	MIXED	Oceania	Austronesian	MIXED	NA	NA	Bergstrom_et_al	Mixed
papuan6278595	MIXED	MIXED	MIXED	MIXED	MIXED	Oceania	MIXED	MIXED	NA	NA	Bergstrom_et_al	Mixed
papuan6278588	MIXED	MIXED	MIXED	MIXED	MIXED	Oceania	Lower_Sepik-Ramu	MIXED	NA	NA	Bergstrom_et_al	Mixed
papuan6278059	MIXED	MIXED	MIXED	MIXED	MIXED	Oceania	Trans-New_Guinea	MIXED	144.45	-6.9	Bergstrom_et_al	Mixed
papuan6278350	Bugawac	Morobe	MOROBE	Lae	PNG_Lowland	Oceania	Austronesian	Bugawac	147.25	-6.7	Bergstrom_et_al	PASS
papuan6277843	Yabem	Morobe	MOROBE	Finschhafen	PNG_Lowland	Oceania	Austronesian	Yabem	147.85	-6.65	Bergstrom_et_al	PASS
papuan6278202	Yabem	Morobe	MOROBE	Finschhafen	PNG_Lowland	Oceania	Austronesian	Yabem	147.85	-6.65	Bergstrom_et_al	PASS
papuan6278099	MIXED	MIXED	MOROBE	MIXED	PNG_Lowland	Oceania	MIXED	MIXED	NA	NA	Bergstrom_et_al	Mixed
papuan6278308	MIXED	MIXED	MOROBE	MIXED	PNG_Lowland	Oceania	MIXED	MIXED	NA	NA	Bergstrom_et_al	Mixed
papuan6278370	MIXED	MIXED	MOROBE	MIXED	PNG_Lowland	Oceania	MIXED	MIXED	NA	NA	Bergstrom_et_al	Mixed
papuan6278585	Kate	Morobe	MOROBE	Finschhafen	PNG_Lowland	Oceania	Trans-New_Guinea	Kate	147.7	-6.45	Bergstrom_et_al	PASS
papuan6278573	Kate	Morobe	MOROBE	Finschhafen	PNG_Lowland	Oceania	Trans-New_Guinea	Kate	147.7	-6.45	Bergstrom_et_al	PASS

papuan6278036	Nabak	Morobe	MOROBE	Lae	PNG_Lowland	Oceania	Trans-New_Guinea	Nabak	147	-6.5	Bergstrom_et_al	PASS
papuan6278530	Patpatar	Manus_New_Ireland	NEW_IRELAND	Namatanai	Bismarck_Arch	Oceania	Austronesian	Patpatar	152.5	-3.75	Bergstrom_et_al	PASS
papuan6278067	Kara	Manus_New_Ireland	NEW_IRELAND	northern_New_Ireland	Bismarck_Arch	Oceania	Austronesian	Kara	151.1	-2.8	Bergstrom_et_al	PASS
papuan6278454	MIXED	MIXED	NORTHERN	MIXED	Massim	Oceania	MIXED	MIXED	NA	NA	Bergstrom_et_al	Mixed
papuan6278357	Orokaiva	Northern	NORTHERN	Popondetta	Massim	Oceania	Trans-New_Guinea	Orokaiva	148.2	-8.75	Bergstrom_et_al	PASS
papuan6278384	Korafe	Northern	NORTHERN	Popondetta	Massim	Oceania	Trans-New_Guinea	Korafe	149.25	-9.05	Bergstrom_et_al	PASS
papuan6278313	Korafe	Northern	NORTHERN	Popondetta	Massim	Oceania	Trans-New_Guinea	Korafe	149.25	-9.05	Bergstrom_et_al	PASS
papuan6278284	Korafe	Northern	NORTHERN	Popondetta	Massim	Oceania	Trans-New_Guinea	Korafe	149.25	-9.05	Bergstrom_et_al	PASS
papuan6278026	Angal	Southern_Highlands	SOUTHERN_HIGHLANDS	Mendi	PNG_Highland	Oceania	Trans-New_Guinea	Angal	143.6	-6	Bergstrom_et_al	PASS
papuan6278039	Angal	Southern_Highlands	SOUTHERN_HIGHLANDS	Mendi	PNG_Highland	Oceania	Trans-New_Guinea	Angal	143.6	-6	Bergstrom_et_al	PASS
papuan6278047	Angal	Southern_Highlands	SOUTHERN_HIGHLANDS	Mendi	PNG_Highland	Oceania	Trans-New_Guinea	Angal	143.6	-6	Bergstrom_et_al	PASS
papuan6278073	Angal	Southern_Highlands	SOUTHERN_HIGHLANDS	Mendi	PNG_Highland	Oceania	Trans-New_Guinea	Angal	143.6	-6	Bergstrom_et_al	PASS
papuan6278097	Angal	Southern_Highlands	SOUTHERN_HIGHLANDS	Mendi	PNG_Highland	Oceania	Trans-New_Guinea	Angal	143.6	-6	Bergstrom_et_al	PASS
papuan6278074	Angal	Southern_Highlands	SOUTHERN_HIGHLANDS	Mendi	PNG_Highland	Oceania	Trans-New_Guinea	Angal	143.6	-6	Bergstrom_et_al	PASS
papuan6278023	Angal	Southern_Highlands	SOUTHERN_HIGHLANDS	Mendi	PNG_Highland	Oceania	Trans-New_Guinea	Angal	143.6	-6	Bergstrom_et_al	PASS
papuan6278209	Angal	Southern_Highlands	SOUTHERN_HIGHLANDS	Mendi	PNG_Highland	Oceania	Trans-New_Guinea	Angal	143.6	-6	Bergstrom_et_al	PASS
papuan6278255	East_Kewa_Aiya	Southern_Highlands	SOUTHERN_HIGHLANDS	Ialibu	PNG_Highland	Oceania	Trans-New_Guinea	East_Kewa_Aiya	143.9	-6.4	Bergstrom_et_al	PASS
papuan6278156	East_Kewa_Aiya	Southern_Highlands	SOUTHERN_HIGHLANDS	Ialibu	PNG_Highland	Oceania	Trans-New_Guinea	East_Kewa_Aiya	143.9	-6.4	Bergstrom_et_al	PASS
papuan6278164	East_Kewa_Aiya	Southern_Highlands	SOUTHERN_HIGHLANDS	Ialibu	PNG_Highland	Oceania	Trans-New_Guinea	East_Kewa_Aiya	143.9	-6.4	Bergstrom_et_al	PASS
papuan6278230	East_Kewa_Aiya	Southern_Highlands	SOUTHERN_HIGHLANDS	Ialibu	PNG_Highland	Oceania	Trans-New_Guinea	East_Kewa_Aiya	143.9	-6.4	Bergstrom_et_al	PASS
papuan6278238	East_Kewa_Aiya	Southern_Highlands	SOUTHERN_HIGHLANDS	Ialibu	PNG_Highland	Oceania	Trans-New_Guinea	East_Kewa_Aiya	143.9	-6.4	Bergstrom_et_al	PASS
papuan6278272	East_Kewa_Aiya	Southern_Highlands	SOUTHERN_HIGHLANDS	Ialibu	PNG_Highland	Oceania	Trans-New_Guinea	East_Kewa_Aiya	143.9	-6.4	Bergstrom_et_al	PASS
papuan6278287	East_Kewa_Aiya	Southern_Highlands	SOUTHERN_HIGHLANDS	Ialibu	PNG_Highland	Oceania	Trans-New_Guinea	East_Kewa_Aiya	143.9	-6.4	Bergstrom_et_al	PASS
papuan6277840	East_Kewa_Aliya	Southern_Highlands	SOUTHERN_HIGHLANDS	Ialibu	PNG_Highland	Oceania	Trans-New_Guinea	East_Kewa_Aliya	143.91	-6.41	Bergstrom_et_al	PASS
papuan6278125	East_Kewa_Aliya	Southern_Highlands	SOUTHERN_HIGHLANDS	Ialibu	PNG_Highland	Oceania	Trans-New_Guinea	East_Kewa_Aliya	143.91	-6.41	Bergstrom_et_al	PASS
papuan6278141	East_Kewa_Aliya	Southern_Highlands	SOUTHERN_HIGHLANDS	Ialibu	PNG_Highland	Oceania	Trans-New_Guinea	East_Kewa_Aliya	143.91	-6.41	Bergstrom_et_al	PASS
papuan6277848	East_Kewa_Aliya	Southern_Highlands	SOUTHERN_HIGHLANDS	Ialibu	PNG_Highland	Oceania	Trans-New_Guinea	East_Kewa_Aliya	143.91	-6.41	Bergstrom_et_al	PASS
papuan6277856	East_Kewa_Aliya	Southern_Highlands	SOUTHERN_HIGHLANDS	Ialibu	PNG_Highland	Oceania	Trans-New_Guinea	East_Kewa_Aliya	143.91	-6.41	Bergstrom_et_al	PASS
papuan6278181	East_Kewa_Aliya	Southern_Highlands	SOUTHERN_HIGHLANDS	Ialibu	PNG_Highland	Oceania	Trans-New_Guinea	East_Kewa_Aliya	143.91	-6.41	Bergstrom_et_al	PASS
papuan6278222	East_Kewa_Aliya	Southern_Highlands	SOUTHERN_HIGHLANDS	Ialibu	PNG_Highland	Oceania	Trans-New_Guinea	East_Kewa_Aliya	143.91	-6.41	Bergstrom_et_al	PASS
papuan6278286	East_Kewa_Aliya	Southern_Highlands	SOUTHERN_HIGHLANDS	Ialibu	PNG_Highland	Oceania	Trans-New_Guinea	East_Kewa_Aliya	143.91	-6.41	Bergstrom_et_al	PASS
papuan6278215	East_Kewa_Aliya	Southern_Highlands	SOUTHERN_HIGHLANDS	Ialibu	PNG_Highland	Oceania	Trans-New_Guinea	East_Kewa_Aliya	143.91	-6.41	Bergstrom_et_al	PASS
papuan6278264	East_Kewa_Aliya	Southern_Highlands	SOUTHERN_HIGHLANDS	Ialibu	PNG_Highland	Oceania	Trans-New_Guinea	East_Kewa_Aliya	143.91	-6.41	Bergstrom_et_al	PASS
papuan6278048	Erave	Southern_Highlands	SOUTHERN_HIGHLANDS	Mendi	PNG_Highland	Oceania	Trans-New_Guinea	Erave	143.95	-6.6	Bergstrom_et_al	PASS
papuan6278138	Erave	Southern_Highlands	SOUTHERN_HIGHLANDS	Mendi	PNG_Highland	Oceania	Trans-New_Guinea	Erave	143.95	-6.6	Bergstrom_et_al	PASS
papuan6278122	Erave	Southern_Highlands	SOUTHERN_HIGHLANDS	Mendi	PNG_Highland	Oceania	Trans-New_Guinea	Erave	143.95	-6.6	Bergstrom_et_al	PASS
papuan6278612	Erave	Southern_Highlands	SOUTHERN_HIGHLANDS	Mendi	PNG_Highland	Oceania	Trans-New_Guinea	Erave	143.95	-6.6	Bergstrom_et_al	Kin
papuan6278123	Erave	Southern_Highlands	SOUTHERN_HIGHLANDS	Mendi	PNG_Highland	Oceania	Trans-New_Guinea	Erave	143.95	-6.6	Bergstrom_et_al	PASS
papuan6278131	Erave	Southern_Highlands	SOUTHERN_HIGHLANDS	Mendi	PNG_Highland	Oceania	Trans-New_Guinea	Erave	143.95	-6.6	Bergstrom_et_al	PASS
papuan6278147	Erave	Southern_Highlands	SOUTHERN_HIGHLANDS	Mendi	PNG_Highland	Oceania	Trans-New_Guinea	Erave	143.95	-6.6	Bergstrom_et_al	PASS
papuan6278187	Erave	Southern_Highlands	SOUTHERN_HIGHLANDS	Mendi	PNG_Highland	Oceania	Trans-New_Guinea	Erave	143.95	-6.6	Bergstrom_et_al	PASS
papuan6278140	Erave	Southern_Highlands	SOUTHERN_HIGHLANDS	Mendi	PNG_Highland	Oceania	Trans-New_Guinea	Erave	143.95	-6.6	Bergstrom_et_al	PASS
papuan6278124	Erave	Southern_Highlands	SOUTHERN_HIGHLANDS	Mendi	PNG_Highland	Oceania	Trans-New_Guinea	Erave	143.95	-6.6	Bergstrom_et_al	PASS
papuan6278214	Erave	Southern_Highlands	SOUTHERN_HIGHLANDS	Mendi	PNG_Highland	Oceania	Trans-New_Guinea	Erave	143.95	-6.6	Bergstrom_et_al	PASS (2nd degree Kinship)
papuan6278278	Erave	Southern_Highlands	SOUTHERN_HIGHLANDS	Mendi	PNG_Highland	Oceania	Trans-New_Guinea	Erave	143.95	-6.6	Bergstrom_et_al	PASS
papuan6278246	Erave	Southern_Highlands	SOUTHERN_HIGHLANDS	Mendi	PNG_Highland	Oceania	Trans-New_Guinea	Erave	143.95	-6.6	Bergstrom_et_al	PASS
papuan6278155	Erave	Southern_Highlands	SOUTHERN_HIGHLANDS	Mendi	PNG_Highland	Oceania	Trans-New_Guinea	Erave	143.95	-6.6	Bergstrom_et_al	PASS
papuan6278066	Foi	Southern_Highlands	SOUTHERN_HIGHLANDS	Mendi	PNG_Highland	Oceania	Trans-New_Guinea	Foi	143.5	-6.5	Bergstrom_et_al	PASS
papuan6278078	Foi	Southern_Highlands	SOUTHERN_HIGHLANDS	Mendi	PNG_Highland	Oceania	Trans-New_Guinea	Foi	143.5	-6.5	Bergstrom_et_al	PASS
papuan6278041	Heneng	Southern_Highlands	SOUTHERN_HIGHLANDS	Mendi	PNG_Highland	Oceania	Trans-New_Guinea	Heneng	143.5	-6.1	Bergstrom_et_al	PASS
papuan6278065	Heneng	Southern_Highlands	SOUTHERN_HIGHLANDS	Mendi	PNG_Highland	Oceania	Trans-New_Guinea	Heneng	143.5	-6.1	Bergstrom_et_al	PASS
papuan6278050	Heneng	Southern_Highlands	SOUTHERN_HIGHLANDS	Mendi	PNG_Highland	Oceania	Trans-New_Guinea	Heneng	143.5	-6.1	Bergstrom_et_al	PASS
papuan6278190	Heneng	Southern_Highlands	SOUTHERN_HIGHLANDS	Mendi	PNG_Highland	Oceania	Trans-New_Guinea	Heneng	143.5	-6.1	Bergstrom_et_al	PASS

papuan6278135	Heneng	Southern_Highlands	SOUTHERN_HIGHLANDS	Mendi	PNG_Highland	Oceania	Trans-New_Guinea	Heneng	143.5	-6.1	Bergstrom_et_al	PASS
papuan6278170	Heneng	Southern_Highlands	SOUTHERN_HIGHLANDS	Mendi	PNG_Highland	Oceania	Trans-New_Guinea	Heneng	143.5	-6.1	Bergstrom_et_al	PASS
papuan6278015	Heneng	Southern_Highlands	SOUTHERN_HIGHLANDS	Mendi	PNG_Highland	Oceania	Trans-New_Guinea	Heneng	143.5	-6.1	Bergstrom_et_al	PASS
papuan6278055	Huli	Southern_Highlands	SOUTHERN_HIGHLANDS	Tauri	PNG_Highland	Oceania	Trans-New_Guinea	Huli	143	-5.95	Bergstrom_et_al	PASS
papuan6278009	Huli	Southern_Highlands	SOUTHERN_HIGHLANDS	Tauri	PNG_Highland	Oceania	Trans-New_Guinea	Huli	143	-5.95	Bergstrom_et_al	PASS
papuan6278096	Huli	Southern_Highlands	SOUTHERN_HIGHLANDS	Tauri	PNG_Highland	Oceania	Trans-New_Guinea	Huli	143	-5.95	Bergstrom_et_al	PASS
papuan6278088	Huli	Southern_Highlands	SOUTHERN_HIGHLANDS	Tauri	PNG_Highland	Oceania	Trans-New_Guinea	Huli	143	-5.95	Bergstrom_et_al	PASS
papuan6278080	Huli	Southern_Highlands	SOUTHERN_HIGHLANDS	Tauri	PNG_Highland	Oceania	Trans-New_Guinea	Huli	143	-5.95	Bergstrom_et_al	PASS
papuan6278089	Huli	Southern_Highlands	SOUTHERN_HIGHLANDS	Tauri	PNG_Highland	Oceania	Trans-New_Guinea	Huli	143	-5.95	Bergstrom_et_al	PASS
papuan6278127	Huli	Southern_Highlands	SOUTHERN_HIGHLANDS	Tauri	PNG_Highland	Oceania	Trans-New_Guinea	Huli	143	-5.95	Bergstrom_et_al	PASS
papuan6278151	Huli	Southern_Highlands	SOUTHERN_HIGHLANDS	Tauri	PNG_Highland	Oceania	Trans-New_Guinea	Huli	143	-5.95	Bergstrom_et_al	PASS
papuan6278112	Huli	Southern_Highlands	SOUTHERN_HIGHLANDS	Tauri	PNG_Highland	Oceania	Trans-New_Guinea	Huli	143	-5.95	Bergstrom_et_al	PASS
papuan6278175	Huli	Southern_Highlands	SOUTHERN_HIGHLANDS	Tauri	PNG_Highland	Oceania	Trans-New_Guinea	Huli	143	-5.95	Bergstrom_et_al	PASS
papuan6278103	Huli	Southern_Highlands	SOUTHERN_HIGHLANDS	Tauri	PNG_Highland	Oceania	Trans-New_Guinea	Huli	143	-5.95	Bergstrom_et_al	PASS
papuan6278119	Huli	Southern_Highlands	SOUTHERN_HIGHLANDS	Tauri	PNG_Highland	Oceania	Trans-New_Guinea	Huli	143	-5.95	Bergstrom_et_al	PASS
papuan6278072	Huli	Southern_Highlands	SOUTHERN_HIGHLANDS	Tauri	PNG_Highland	Oceania	Trans-New_Guinea	Huli	143	-5.95	Bergstrom_et_al	PASS
papuan6278120	Huli	Southern_Highlands	SOUTHERN_HIGHLANDS	Tauri	PNG_Highland	Oceania	Trans-New_Guinea	Huli	143	-5.95	Bergstrom_et_al	PASS
papuan6278183	Huli	Southern_Highlands	SOUTHERN_HIGHLANDS	Tauri	PNG_Highland	Oceania	Trans-New_Guinea	Huli	143	-5.95	Bergstrom_et_al	PASS
papuan6278168	Huli	Southern_Highlands	SOUTHERN_HIGHLANDS	Tauri	PNG_Highland	Oceania	Trans-New_Guinea	Huli	143	-5.95	Bergstrom_et_al	PASS
papuan6278105	Huli	Southern_Highlands	SOUTHERN_HIGHLANDS	Tauri	PNG_Highland	Oceania	Trans-New_Guinea	Huli	143	-5.95	Bergstrom_et_al	PASS
papuan6278113	Huli	Southern_Highlands	SOUTHERN_HIGHLANDS	Tauri	PNG_Highland	Oceania	Trans-New_Guinea	Huli	143	-5.95	Bergstrom_et_al	PASS
papuan6278121	Huli	Southern_Highlands	SOUTHERN_HIGHLANDS	Tauri	PNG_Highland	Oceania	Trans-New_Guinea	Huli	143	-5.95	Bergstrom_et_al	PASS
papuan6278232	Huli	Southern_Highlands	SOUTHERN_HIGHLANDS	Tauri	PNG_Highland	Oceania	Trans-New_Guinea	Huli	143	-5.95	Bergstrom_et_al	PASS
papuan6278090	Imbongu	Southern_Highlands	SOUTHERN_HIGHLANDS	Ialibu	PNG_Highland	Oceania	Trans-New_Guinea	Imbongu	144	-6.15	Bergstrom_et_al	PASS
papuan6278082	Imbongu	Southern_Highlands	SOUTHERN_HIGHLANDS	Ialibu	PNG_Highland	Oceania	Trans-New_Guinea	Imbongu	144	-6.15	Bergstrom_et_al	PASS
papuan6278194	Imbongu	Southern_Highlands	SOUTHERN_HIGHLANDS	Ialibu	PNG_Highland	Oceania	Trans-New_Guinea	Imbongu	144	-6.15	Bergstrom_et_al	PASS
papuan6278008	Imbongu	Southern_Highlands	SOUTHERN_HIGHLANDS	Ialibu	PNG_Highland	Oceania	Trans-New_Guinea	Imbongu	144	-6.15	Bergstrom_et_al	PASS
papuan6278250	Samberigi	Southern_Highlands	SOUTHERN_HIGHLANDS	Lake_Kutubu	PNG_Highland	Oceania	Trans-New_Guinea	Samberigi	144	-6.7	Bergstrom_et_al	PASS
papuan6278017	West_Kewa	Southern_Highlands	SOUTHERN_HIGHLANDS	Mendi	PNG_Highland	Oceania	Trans-New_Guinea	West_Kewa	143.75	-6.3	Bergstrom_et_al	PASS
papuan6278153	Wiru	Southern_Highlands	SOUTHERN_HIGHLANDS	Ialibu	PNG_Highland	Oceania	Trans-New_Guinea	Wiru	144.2	-6.4	Bergstrom_et_al	PASS
papuan6278177	Wiru	Southern_Highlands	SOUTHERN_HIGHLANDS	Ialibu	PNG_Highland	Oceania	Trans-New_Guinea	Wiru	144.2	-6.4	Bergstrom_et_al	PASS
papuan6278193	Wiru	Southern_Highlands	SOUTHERN_HIGHLANDS	Ialibu	PNG_Highland	Oceania	Trans-New_Guinea	Wiru	144.2	-6.4	Bergstrom_et_al	PASS
papuan6278106	Wiru	Southern_Highlands	SOUTHERN_HIGHLANDS	Ialibu	PNG_Highland	Oceania	Trans-New_Guinea	Wiru	144.2	-6.4	Bergstrom_et_al	PASS
papuan6278114	Wiru	Southern_Highlands	SOUTHERN_HIGHLANDS	Ialibu	PNG_Highland	Oceania	Trans-New_Guinea	Wiru	144.2	-6.4	Bergstrom_et_al	PASS
papuan6278107	Wiru	Southern_Highlands	SOUTHERN_HIGHLANDS	Ialibu	PNG_Highland	Oceania	Trans-New_Guinea	Wiru	144.2	-6.4	Bergstrom_et_al	PASS
papuan6278178	Wiru	Southern_Highlands	SOUTHERN_HIGHLANDS	Ialibu	PNG_Highland	Oceania	Trans-New_Guinea	Wiru	144.2	-6.4	Bergstrom_et_al	Kin
papuan6278186	Wiru	Southern_Highlands	SOUTHERN_HIGHLANDS	Ialibu	PNG_Highland	Oceania	Trans-New_Guinea	Wiru	144.2	-6.4	Bergstrom_et_al	PASS
papuan6278162	Wiru	Southern_Highlands	SOUTHERN_HIGHLANDS	Ialibu	PNG_Highland	Oceania	Trans-New_Guinea	Wiru	144.2	-6.4	Bergstrom_et_al	PASS
papuan6278145	Wiru	Southern_Highlands	SOUTHERN_HIGHLANDS	Ialibu	PNG_Highland	Oceania	Trans-New_Guinea	Wiru	144.2	-6.4	Bergstrom_et_al	PASS
papuan6278247	Wiru	Southern_Highlands	SOUTHERN_HIGHLANDS	Ialibu	PNG_Highland	Oceania	Trans-New_Guinea	Wiru	144.2	-6.4	Bergstrom_et_al	PASS
papuan6278271	Wiru	Southern_Highlands	SOUTHERN_HIGHLANDS	Ialibu	PNG_Highland	Oceania	Trans-New_Guinea	Wiru	144.2	-6.4	Bergstrom_et_al	PASS
papuan6277815	Kyaka	Western_Highlands	WESTERN_HIGHLANDS	Wabag_Koles	PNG_Highland	Oceania	Trans-New_Guinea	Kyaka	144.1	-5.5	Bergstrom_et_al	PASS
papuan6277875	Maring	Western_Highlands	WESTERN_HIGHLANDS	Hagen	PNG_Highland	Oceania	Trans-New_Guinea	Maring	144.6	-5.5	Bergstrom_et_al	PASS
papuan6278043	Mbo-Ung	Western_Highlands	WESTERN_HIGHLANDS	Hagen	PNG_Highland	Oceania	Trans-New_Guinea	Mbo-Ung	144.1	-5.9	Bergstrom_et_al	PASS
papuan6277863	Mbo-Ung	Western_Highlands	WESTERN_HIGHLANDS	Hagen	PNG_Highland	Oceania	Trans-New_Guinea	Mbo-Ung	144.1	-5.9	Bergstrom_et_al	PASS
papuan6277862	Melpa	Western_Highlands	WESTERN_HIGHLANDS	Hagen	PNG_Highland	Oceania	Trans-New_Guinea	Melpa	144.3	-5.65	Bergstrom_et_al	PASS
papuan6277903	Melpa	Western_Highlands	WESTERN_HIGHLANDS	Hagen	PNG_Highland	Oceania	Trans-New_Guinea	Melpa	144.3	-5.65	Bergstrom_et_al	PASS
papuan6277839	Melpa	Western_Highlands	WESTERN_HIGHLANDS	Hagen	PNG_Highland	Oceania	Trans-New_Guinea	Melpa	144.3	-5.65	Bergstrom_et_al	PASS
papuan6277887	MIXED	Western_Highlands	WESTERN_HIGHLANDS	MIXED	PNG_Highland	Oceania	Trans-New_Guinea	MIXED	NA	NA	Bergstrom_et_al	PASS
papuan6277895	MIXED	Western_Highlands	WESTERN_HIGHLANDS	MIXED	PNG_Highland	Oceania	Trans-New_Guinea	MIXED	NA	NA	Bergstrom_et_al	PASS
papuan6278225	Nii	Western_Highlands	WESTERN_HIGHLANDS	Hagen	PNG_Highland	Oceania	Trans-New_Guinea	Nii	144.5	-5.8	Bergstrom_et_al	PASS
papuan6277942	Nii	Western_Highlands	WESTERN_HIGHLANDS	Hagen	PNG_Highland	Oceania	Trans-New_Guinea	Nii	144.5	-5.8	Bergstrom_et_al	PASS
papuan6278098	Nii	Western_Highlands	WESTERN_HIGHLANDS	Hagen	PNG_Highland	Oceania	Trans-New_Guinea	Nii	144.5	-5.8	Bergstrom_et_al	PASS

papuan6277831	Nii	Western_Highlands	WESTERN_HIGHLANDS	Hagen	PNG_Highland	Oceania	Trans-New_Guinea	Nii	144.5	-5.8	Bergstrom_et_al	PASS
papuan6277855	MIXED	Western_Highlands	WESTERN_HIGHLANDS	MIXED	PNG_Highland	Oceania	Trans-New_Guinea	MIXED	NA	NA	Bergstrom_et_al	PASS
papuan6278233	MIXED	Western_Highlands	WESTERN_HIGHLANDS	MIXED	PNG_Highland	Oceania	Trans-New_Guinea	MIXED	NA	NA	Bergstrom_et_al	PASS
papuan6277838	Umbu-Ungu	Western_Highlands	WESTERN_HIGHLANDS	Tambul-Kaugel	PNG_Highland	Oceania	Trans-New_Guinea	Umbu-Ungu	143.9	-5.9	Bergstrom_et_al	PASS
papuan6277894	Umbu-Ungu	Western_Highlands	WESTERN_HIGHLANDS	Tambul-Kaugel	PNG_Highland	Oceania	Trans-New_Guinea	Umbu-Ungu	143.9	-5.9	Bergstrom_et_al	PASS
papuan6277878	Umbu-Ungu	Western_Highlands	WESTERN_HIGHLANDS	Tambul-Kaugel	PNG_Highland	Oceania	Trans-New_Guinea	Umbu-Ungu	143.9	-5.9	Bergstrom_et_al	PASS
papuan6278013	Umbu-Ungu	Western_Highlands	WESTERN_HIGHLANDS	Tambul-Kaugel	PNG_Highland	Oceania	Trans-New_Guinea	Umbu-Ungu	143.9	-5.9	Bergstrom_et_al	PASS
papuan6277912	Wahgi	Western_Highlands	WESTERN_HIGHLANDS	Minj	PNG_Highland	Oceania	Trans-New_Guinea	Wahgi	144.7	-5.9	Bergstrom_et_al	PASS
papuan6277961	Wahgi	Western_Highlands	WESTERN_HIGHLANDS	Minj	PNG_Highland	Oceania	Trans-New_Guinea	Wahgi	144.7	-5.9	Bergstrom_et_al	PASS
papuan6277914	Wahgi	Western_Highlands	WESTERN_HIGHLANDS	Minj	PNG_Highland	Oceania	Trans-New_Guinea	Wahgi	144.7	-5.9	Bergstrom_et_al	PASS
papuan6277932	Wahgi	Western_Highlands	WESTERN_HIGHLANDS	Minj	PNG_Highland	Oceania	Trans-New_Guinea	Wahgi	144.7	-5.9	Bergstrom_et_al	PASS
papuan6278608	Wahgi	Western_Highlands	WESTERN_HIGHLANDS	Minj	PNG_Highland	Oceania	Trans-New_Guinea	Wahgi	144.7	-5.9	Bergstrom_et_al	PASS
papuan6277830	Wahgi	Western_Highlands	WESTERN_HIGHLANDS	Minj	PNG_Highland	Oceania	Trans-New_Guinea	Wahgi	144.7	-5.9	Bergstrom_et_al	PASS
papuan6277913	Wahgi	Western_Highlands	WESTERN_HIGHLANDS	Minj	PNG_Highland	Oceania	Trans-New_Guinea	Wahgi	144.7	-5.9	Bergstrom_et_al	PASS
papuan6278323	MIXED	MIXED	WESTERN	MIXED	PNG_Lowland	Oceania	MIXED	MIXED	NA	NA	Bergstrom_et_al	Mixed
papuan6278282	Southern_Kiwai	Western	WESTERN	Daru	PNG_Lowland	Oceania	Trans-New_Guinea	Southern_Kiwai	143.3	-8.7	Bergstrom_et_al	PASS
papuan6278305	Southern_Kiwai	Western	WESTERN	Daru	PNG_Lowland	Oceania	Trans-New_Guinea	Southern_Kiwai	143.3	-8.7	Bergstrom_et_al	PASS
papuan6278326	Southern_Kiwai	Western	WESTERN	Daru	PNG_Lowland	Oceania	Trans-New_Guinea	Southern_Kiwai	143.3	-8.7	Bergstrom_et_al	PASS
papuan6278361	Southern_Kiwai	Western	WESTERN	Daru	PNG_Lowland	Oceania	Trans-New_Guinea	Southern_Kiwai	143.3	-8.7	Bergstrom_et_al	PASS
papuan6278345	Southern_Kiwai	Western	WESTERN	Daru	PNG_Lowland	Oceania	Trans-New_Guinea	Southern_Kiwai	143.3	-8.7	Bergstrom_et_al	PASS
LP6005592-DNA_C03	Mbuti	Africa	NA	NA	Non_Oceania	Africa	Central_Sudanic	Efe	29	1	Mallick_et_al	PASS
LP6005441-DNA_B08	Mbuti	Africa	NA	NA	Non_Oceania	Africa	Central_Sudanic	Efe	29	1	Mallick_et_al	PASS
LP6005441-DNA_A08	Mbuti	Africa	NA	NA	Non_Oceania	Africa	Central_Sudanic	Efe	29	1	Mallick_et_al	PASS
SS6004471	Mbuti	Africa	NA	NA	Non_Oceania	Africa	Central_Sudanic	Efe	29	1	Mallick_et_al	PASS
LP6005442-DNA_B02	Yoruba	Africa	NA	NA	Non_Oceania	Africa	Niger-Congo	Yoruba	3.9	7.4	Mallick_et_al	PASS
LP6005442-DNA_A02	Yoruba	Africa	NA	NA	Non_Oceania	Africa	Niger-Congo	Yoruba	3.9	7.4	Mallick_et_al	PASS
SS6004475	Yoruba	Africa	NA	NA	Non_Oceania	Africa	Niger-Congo	Yoruba	3.9	7.4	Mallick_et_al	PASS
LP6005441-DNA_A05	French	WestEurasia	NA	NA	Non_Oceania	WestEurasia	Indo-European	French	2	46	Mallick_et_al	PASS
LP6005441-DNA_B05	French	WestEurasia	NA	NA	Non_Oceania	WestEurasia	Indo-European	French	2	46	Mallick_et_al	PASS
SS6004468	French	WestEurasia	NA	NA	Non_Oceania	WestEurasia	Indo-European	French	2	46	Mallick_et_al	PASS
LP6005442-DNA_A11	Spanish	WestEurasia	NA	NA	Non_Oceania	WestEurasia	Indo-European	Spanish	-4	39.9	Mallick_et_al	PASS
LP6005442-DNA_B11	Spanish	WestEurasia	NA	NA	Non_Oceania	WestEurasia	Indo-European	Spanish	-4	39.9	Mallick_et_al	PASS
LP6005443-DNA_H01	Tu	EastAsia	NA	NA	Non_Oceania	EastAsia	Mongolian	Tu	101	36	Mallick_et_al	PASS
LP6005441-DNA_D12	Tu	EastAsia	NA	NA	Non_Oceania	EastAsia	Mongolian	Tu	101	36	Mallick_et_al	PASS
LP6005441-DNA_D05	Han	EastAsia	NA	NA	Non_Oceania	EastAsia	Sino-Tibetan	Mandarin	114	32.3	Mallick_et_al	PASS
LP6005441-DNA_C05	Han	EastAsia	NA	NA	Non_Oceania	EastAsia	Sino-Tibetan	Mandarin	114	32.3	Mallick_et_al	PASS
LP6005443-DNA_A02	Tujia	EastAsia	NA	NA	Non_Oceania	EastAsia	Sino-Tibetan	Tujia	109	29	Mallick_et_al	PASS
LP6005441-DNA_F12	Tujia	EastAsia	NA	NA	Non_Oceania	EastAsia	Sino-Tibetan	Tujia	109	29	Mallick_et_al	PASS
LP6005443-DNA_E09	Naxi	EastAsia	NA	NA	Non_Oceania	EastAsia	Sino-Tibetan	Naxi	100	26	Mallick_et_al	PASS
LP6005441-DNA_B09	Naxi	EastAsia	NA	NA	Non_Oceania	EastAsia	Sino-Tibetan	Naxi	100	26	Mallick_et_al	PASS
LP6005442-DNA_H01	Yi	EastAsia	NA	NA	Non_Oceania	EastAsia	Sino-Tibetan	Yi	103	28	Mallick_et_al	PASS
LP6005441-DNA_B07	Lahu	EastAsia	NA	NA	Non_Oceania	EastAsia	Sino-Tibetan	Lahu	100	22	Mallick_et_al	PASS
LP6005443-DNA_E01	Lahu	EastAsia	NA	NA	Non_Oceania	EastAsia	Sino-Tibetan	Lahu	100	22	Mallick_et_al	PASS
LP6005441-DNA_C08	Miao	EastAsia	NA	NA	Non_Oceania	EastAsia	Hmong-Mien	Miao	109	28	Mallick_et_al	PASS
LP6005441-DNA_D08	Miao	EastAsia	NA	NA	Non_Oceania	EastAsia	Hmong-Mien	Miao	109	28	Mallick_et_al	PASS
LP6005443-DNA_G01	She	EastAsia	NA	NA	Non_Oceania	EastAsia	Hmong-Mien	She	119	27	Mallick_et_al	PASS
LP6005443-DNA_F01	She	EastAsia	NA	NA	Non_Oceania	EastAsia	Hmong-Mien	She	119	27	Mallick_et_al	PASS
LP6005592-DNA_D03	Dai	EastAsia	NA	NA	Non_Oceania	EastAsia	Tai-Kadai	Central_Tai	100	21	Mallick_et_al	PASS
LP6005443-DNA_B01	Dai	EastAsia	NA	NA	Non_Oceania	EastAsia	Tai-Kadai	Central_Tai	100	21	Mallick_et_al	PASS
LP6005441-DNA_D04	Dai	EastAsia	NA	NA	Non_Oceania	EastAsia	Tai-Kadai	Central_Tai	100	21	Mallick_et_al	PASS
SS6004467	Dai	EastAsia	NA	NA	Non_Oceania	EastAsia	Tai-Kadai	Central_Tai	100	21	Mallick_et_al	PASS
LP6005443-DNA_A07	Thai	EastAsia	NA	NA	Non_Oceania	EastAsia	Tai-Kadai	Central_Tai	100.5	13.8	Mallick_et_al	PASS
LP6005443-DNA_B07	Thai	EastAsia	NA	NA	Non_Oceania	EastAsia	Tai-Kadai	Central_Tai	100.5	13.8	Mallick_et_al	PASS

LP6005442-DNA_D11	Kinh	EastAsia	NA	NA	Non_Oceania	EastAsia	Austro-Asiatic	Vietnamese	105.9	21	Mallick_et_al	PASS
LP6005442-DNA_C11	Kinh	EastAsia	NA	NA	Non_Oceania	EastAsia	Austro-Asiatic	Vietnamese	105.9	21	Mallick_et_al	PASS
LP6005519-DNA_B06	Burmese	EastAsia	NA	NA	Non_Oceania	EastAsia	Sino-Tibetan	Burmese	96.7	17	Mallick_et_al	PASS
LP6005519-DNA_A06	Burmese	EastAsia	NA	NA	Non_Oceania	EastAsia	Sino-Tibetan	Burmese	96.7	17	Mallick_et_al	PASS
LP6005442-DNA_C07	Ami	EastAsia_AN	NA	NA	Non_Oceania	EastAsia	Austronesian	Amis	121.19	22.84	Mallick_et_al	PASS
LP6005443-DNA_G05	Ami	EastAsia_AN	NA	NA	Non_Oceania	EastAsia	Austronesian	Amis	121.19	22.84	Mallick_et_al	PASS
LP6005442-DNA_E07	Atayal	EastAsia_AN	NA	NA	Non_Oceania	EastAsia	Austronesian	Atayal	121.3	24.61	Mallick_et_al	PASS
LP6005519-DNA_C06	Igorot	EastAsia_AN	NA	NA	Non_Oceania	EastAsia	Austronesian	Northern_Luzon	121	17.1	Mallick_et_al	PASS
LP6005519-DNA_D06	Igorot	EastAsia_AN	NA	NA	Non_Oceania	EastAsia	Austronesian	Northern_Luzon	121	17.1	Mallick_et_al	PASS
LP6005519-DNA_F06	Dusun	EastAsia_AN	NA	NA	Non_Oceania	EastAsia	Austronesian	Dusun	114.7	4.7	Mallick_et_al	PASS
LP6005519-DNA_E06	Dusun	EastAsia_AN	NA	NA	Non_Oceania	EastAsia	Austronesian	Dusun	114.7	4.7	Mallick_et_al	PASS
SS6004478	Australian	Australia	NA	NA	Australia	Oceania	Pama-Nyungan	Paman	143	-13	Mallick_et_al	PASS
SS6004477	Australian	Australia	NA	NA	Australia	Oceania	Pama-Nyungan	Paman	143	-13	Mallick_et_al	PASS
LP6005441-DNA_B03	Bougainville	Bougainville	BOUGAINVILLE	Bougainville	Solomon_Arch	Oceania	NA	NA	155	-6	Mallick_et_al	PASS
LP6005441-DNA_A03	Bougainville	Bougainville	BOUGAINVILLE	Bougainville	Solomon_Arch	Oceania	NA	NA	155	-6	Mallick_et_al	PASS
UV500	Lavongai	Manus_New_Ireland	NEW_IRELAND	North_Lavongai	Bismarck_Arch	Oceania	Austronesian	Lavongai	150.27	-2.53	Vernot_et_al	PASS
UV518	Mussau	Manus_New_Ireland	NEW_IRELAND	Kaupgu	Bismarck_Arch	Oceania	Austronesian	Mussau	149.73	-1.58	Vernot_et_al	PASS
UV573	Nailik	Manus_New_Ireland	NEW_IRELAND	Nailik	Bismarck_Arch	Oceania	Austronesian	Nalik	151.3	-2.94	Vernot_et_al	PASS
UV580	Nailik	Manus_New_Ireland	NEW_IRELAND	Nailik	Bismarck_Arch	Oceania	Austronesian	Nalik	151.3	-2.94	Vernot_et_al	Kin
UV043	Baining	East_New_Britain	EAST_NEW_BRITAIN	Mali	Bismarck_Arch	Oceania	East_New_Britain	Mali	152	-4.52	Vernot_et_al	PASS
UV305	Baining	East_New_Britain	EAST_NEW_BRITAIN	Kaket	Bismarck_Arch	Oceania	East_New_Britain	Qaqet	152	-4.52	Vernot_et_al	PASS
UV1134	Ata	West_New_Britain	WEST_NEW_BRITAIN	Luge	Bismarck_Arch	Oceania	Yele-West_New_Britain	Pele-Ata	151.03	-5.57	Vernot_et_al	PASS
UV1230	Ata	West_New_Britain	WEST_NEW_BRITAIN	Uasilau	Bismarck_Arch	Oceania	Yele-West_New_Britain	Pele-Ata	151.03	-5.57	Vernot_et_al	PASS
UV1042	Mamusi	West_New_Britain	WEST_NEW_BRITAIN	Kisiluvi	Bismarck_Arch	Oceania	Austronesian	Mamusi	150.97	-5.87	Vernot_et_al	PASS
UV1224	Mamusi	West_New_Britain	WEST_NEW_BRITAIN	Paleabu	Bismarck_Arch	Oceania	Austronesian	Mamusi	150.97	-5.87	Vernot_et_al	PASS
UV1196	Melamala	West_New_Britain	WEST_NEW_BRITAIN	Ubili	Bismarck_Arch	Oceania	Austronesian	Meramera	151.33	-5.02	Vernot_et_al	PASS
UV1263	Pasismanua	West_New_Britain	WEST_NEW_BRITAIN	Poronga	Bismarck_Arch	Oceania	Austronesian	Pasismanua	150.09	-6.27	Vernot_et_al	PASS
UV1266	Pasismanua	West_New_Britain	WEST_NEW_BRITAIN	Poronga	Bismarck_Arch	Oceania	Austronesian	Pasismanua	150.09	-6.27	Vernot_et_al	Kin
UV886	Nakanai_bileki	West_New_Britain	WEST_NEW_BRITAIN	Bileki	Bismarck_Arch	Oceania	Austronesian	Nakanai	150.66	-5.67	Vernot_et_al	PASS
UV897	Nakanai_bileki	West_New_Britain	WEST_NEW_BRITAIN	Bileki	Bismarck_Arch	Oceania	Austronesian	Nakanai	150.66	-5.67	Vernot_et_al	PASS
UV910	Nakanai_bileki	West_New_Britain	WEST_NEW_BRITAIN	Bileki	Bismarck_Arch	Oceania	Austronesian	Nakanai	150.66	-5.67	Vernot_et_al	PASS
UV919	Nakanai_bileki	West_New_Britain	WEST_NEW_BRITAIN	Bileki	Bismarck_Arch	Oceania	Austronesian	Nakanai	150.66	-5.67	Vernot_et_al	PASS
UV923	Nakanai_bileki	West_New_Britain	WEST_NEW_BRITAIN	Bileki	Bismarck_Arch	Oceania	Austronesian	Nakanai	150.66	-5.67	Vernot_et_al	PASS
UV925	Nakanai_bileki	West_New_Britain	WEST_NEW_BRITAIN	Bileki	Bismarck_Arch	Oceania	Austronesian	Nakanai	150.66	-5.67	Vernot_et_al	PASS
UV927	Nakanai_bileki	West_New_Britain	WEST_NEW_BRITAIN	Bileki	Bismarck_Arch	Oceania	Austronesian	Nakanai	150.66	-5.67	Vernot_et_al	Kin
UV929	Nakanai_bileki	West_New_Britain	WEST_NEW_BRITAIN	Bileki	Bismarck_Arch	Oceania	Austronesian	Nakanai	150.66	-5.67	Vernot_et_al	PASS
UV931	Nakanai_bileki	West_New_Britain	WEST_NEW_BRITAIN	Bileki	Bismarck_Arch	Oceania	Austronesian	Nakanai	150.66	-5.67	Vernot_et_al	PASS
UV940	Nakanai_bileki	West_New_Britain	WEST_NEW_BRITAIN	Bileki	Bismarck_Arch	Oceania	Austronesian	Nakanai	150.66	-5.67	Vernot_et_al	PASS
UV944	Nakanai_bileki	West_New_Britain	WEST_NEW_BRITAIN	Bileki	Bismarck_Arch	Oceania	Austronesian	Nakanai	150.66	-5.67	Vernot_et_al	PASS
UV946	Nakanai	West_New_Britain	WEST_NEW_BRITAIN	Bileki	Bismarck_Arch	Oceania	Austronesian	Nakanai	150.66	-5.67	Vernot_et_al	PASS
UV952	Nakanai	West_New_Britain	WEST_NEW_BRITAIN	Bileki	Bismarck_Arch	Oceania	Austronesian	Nakanai	150.66	-5.67	Vernot_et_al	Kin
UV956	Nakanai	West_New_Britain	WEST_NEW_BRITAIN	Bileki	Bismarck_Arch	Oceania	Austronesian	Nakanai	150.66	-5.67	Vernot_et_al	PASS (2nd degree Kinship)
UV958	Nakanai	West_New_Britain	WEST_NEW_BRITAIN	Bileki	Bismarck_Arch	Oceania	Austronesian	Nakanai	150.66	-5.67	Vernot_et_al	PASS
UV964	Nakanai	West_New_Britain	WEST_NEW_BRITAIN	Bileki	Bismarck_Arch	Oceania	Austronesian	Nakanai	150.66	-5.67	Vernot_et_al	PASS
UV971	Nakanai	West_New_Britain	WEST_NEW_BRITAIN	Bileki	Bismarck_Arch	Oceania	Austronesian	Nakanai	150.66	-5.67	Vernot_et_al	PASS
UV979	Nakanai	West_New_Britain	WEST_NEW_BRITAIN	Bileki	Bismarck_Arch	Oceania	Austronesian	Nakanai	150.66	-5.67	Vernot_et_al	Kin
UV986	Nakanai	West_New_Britain	WEST_NEW_BRITAIN	Bileki	Bismarck_Arch	Oceania	Austronesian	Nakanai	150.66	-5.67	Vernot_et_al	PASS
B00FLGA	Atayal	EastAsia_AN	NA	NA	Non_Oceania	EastAsia	Austronesian	Atayal	121.3	24.61	Choin_et_al	PASS
B00FLGM	Atayal	EastAsia_AN	NA	NA	Non_Oceania	EastAsia	Austronesian	Atayal	121.3	24.61	Choin_et_al	PASS
B00FLGQ	Atayal	EastAsia_AN	NA	NA	Non_Oceania	EastAsia	Austronesian	Atayal	121.3	24.61	Choin_et_al	PASS
B00FLGU	Atayal	EastAsia_AN	NA	NA	Non_Oceania	EastAsia	Austronesian	Atayal	121.3	24.61	Choin_et_al	PASS
B00FLH2	Atayal	EastAsia_AN	NA	NA	Non_Oceania	EastAsia	Austronesian	Atayal	121.3	24.61	Choin_et_al	PASS

B00FLH6	Atayal	EastAsia_AN	NA	NA	Non_Oceania	EastAsia	Austronesian	Atayal	121.3	24.61	Choin_et_al	PASS
B00FLHA	Atayal	EastAsia_AN	NA	NA	Non_Oceania	EastAsia	Austronesian	Atayal	121.3	24.61	Choin_et_al	PASS
B00FLGE	Atayal	EastAsia_AN	NA	NA	Non_Oceania	EastAsia	Austronesian	Atayal	121.3	24.61	Choin_et_al	PASS
B00FLGI	Atayal	EastAsia_AN	NA	NA	Non_Oceania	EastAsia	Austronesian	Atayal	121.3	24.61	Choin_et_al	PASS
B00FLGY	Atayal	EastAsia_AN	NA	NA	Non_Oceania	EastAsia	Austronesian	Atayal	121.3	24.61	Choin_et_al	PASS
B00FLHE	Paiwan	EastAsia_AN	NA	NA	Non_Oceania	EastAsia	Austronesian	Paiwan	120.69	22.68	Choin_et_al	PASS
B00FLHM	Paiwan	EastAsia_AN	NA	NA	Non_Oceania	EastAsia	Austronesian	Paiwan	120.69	22.68	Choin_et_al	Kin
B00FLHQ	Paiwan	EastAsia_AN	NA	NA	Non_Oceania	EastAsia	Austronesian	Paiwan	120.69	22.68	Choin_et_al	PASS
B00FLHU	Paiwan	EastAsia_AN	NA	NA	Non_Oceania	EastAsia	Austronesian	Paiwan	120.69	22.68	Choin_et_al	PASS
B00FLI2	Paiwan	EastAsia_AN	NA	NA	Non_Oceania	EastAsia	Austronesian	Paiwan	120.69	22.68	Choin_et_al	PASS (2nd degree Kinship)
B00FLIA	Paiwan	EastAsia_AN	NA	NA	Non_Oceania	EastAsia	Austronesian	Paiwan	120.69	22.68	Choin_et_al	PASS
B00FLHI	Paiwan	EastAsia_AN	NA	NA	Non_Oceania	EastAsia	Austronesian	Paiwan	120.69	22.68	Choin_et_al	PASS
B00FLHY	Paiwan	EastAsia_AN	NA	NA	Non_Oceania	EastAsia	Austronesian	Paiwan	120.69	22.68	Choin_et_al	PASS
B00FLIE	Paiwan	EastAsia_AN	NA	NA	Non_Oceania	EastAsia	Austronesian	Paiwan	120.69	22.68	Choin_et_al	PASS
B00I2BM	Vella_Lavella	Vella_Lavella	NA	Vella_Lavella	Solomon_Arch	Oceania	Central_Solomons	Bilua	156.37	-7.76	Choin_et_al	PASS
B00I2BN	Vella_Lavella	Vella_Lavella	NA	Vella_Lavella	Solomon_Arch	Oceania	Central_Solomons	Bilua	156.37	-7.76	Choin_et_al	PASS
B00I2BP	Vella_Lavella	Vella_Lavella	NA	Vella_Lavella	Solomon_Arch	Oceania	Central_Solomons	Bilua	156.37	-7.76	Choin_et_al	PASS
B00I2BQ	Vella_Lavella	Vella_Lavella	NA	Vella_Lavella	Solomon_Arch	Oceania	Central_Solomons	Bilua	156.37	-7.76	Choin_et_al	PASS
B00I2BR	Vella_Lavella	Vella_Lavella	NA	Vella_Lavella	Solomon_Arch	Oceania	Central_Solomons	Bilua	156.37	-7.76	Choin_et_al	PASS
B00I2BS	Vella_Lavella	Vella_Lavella	NA	Vella_Lavella	Solomon_Arch	Oceania	Central_Solomons	Bilua	156.37	-7.76	Choin_et_al	PASS (2nd degree Kinship)
B00I2BT	Vella_Lavella	Vella_Lavella	NA	Vella_Lavella	Solomon_Arch	Oceania	Central_Solomons	Bilua	156.37	-7.76	Choin_et_al	PASS
B00I2BU	Vella_Lavella	Vella_Lavella	NA	Vella_Lavella	Solomon_Arch	Oceania	Central_Solomons	Bilua	156.37	-7.76	Choin_et_al	PASS
B00I2BV	Vella_Lavella	Vella_Lavella	NA	Vella_Lavella	Solomon_Arch	Oceania	Central_Solomons	Bilua	156.37	-7.76	Choin_et_al	PASS
B00I2BW	Vella_Lavella	Vella_Lavella	NA	Vella_Lavella	Solomon_Arch	Oceania	Central_Solomons	Bilua	156.37	-7.76	Choin_et_al	PASS
B00I2BX	Vella_Lavella	Vella_Lavella	NA	Vella_Lavella	Solomon_Arch	Oceania	Central_Solomons	Bilua	156.37	-7.76	Choin_et_al	PASS
B00I2BY	Vella_Lavella	Vella_Lavella	NA	Vella_Lavella	Solomon_Arch	Oceania	Central_Solomons	Bilua	156.37	-7.76	Choin_et_al	PASS
B00I2BZ	Vella_Lavella	Vella_Lavella	NA	Vella_Lavella	Solomon_Arch	Oceania	Central_Solomons	Bilua	156.37	-7.76	Choin_et_al	PASS
B00I2C0	Vella_Lavella	Vella_Lavella	NA	Vella_Lavella	Solomon_Arch	Oceania	Central_Solomons	Bilua	156.37	-7.76	Choin_et_al	PASS
B00I2C1	Vella_Lavella	Vella_Lavella	NA	Vella_Lavella	Solomon_Arch	Oceania	Central_Solomons	Bilua	156.37	-7.76	Choin_et_al	PASS
B00I2C2	Vella_Lavella	Vella_Lavella	NA	Vella_Lavella	Solomon_Arch	Oceania	Central_Solomons	Bilua	156.37	-7.76	Choin_et_al	PASS
B00I2C3	Vella_Lavella	Vella_Lavella	NA	Vella_Lavella	Solomon_Arch	Oceania	Central_Solomons	Bilua	156.37	-7.76	Choin_et_al	PASS
B00I2C4	Vella_Lavella	Vella_Lavella	NA	Vella_Lavella	Solomon_Arch	Oceania	Central_Solomons	Bilua	156.37	-7.76	Choin_et_al	PASS
B00I2B2	Malaita	Malaita	NA	Malaita	Solomon_Arch	Oceania	Austronesian	Malaitan	159.95	-9.02	Choin_et_al	PASS
B00I2B8	Malaita	Malaita	NA	Malaita	Solomon_Arch	Oceania	Austronesian	Malaitan	159.95	-9.02	Choin_et_al	PASS
B00I2B3	Malaita	Malaita	NA	Malaita	Solomon_Arch	Oceania	Austronesian	Malaitan	159.95	-9.02	Choin_et_al	PASS
B00I2B4	Malaita	Malaita	NA	Malaita	Solomon_Arch	Oceania	Austronesian	Malaitan	159.95	-9.02	Choin_et_al	PASS
B00I2B5	Malaita	Malaita	NA	Malaita	Solomon_Arch	Oceania	Austronesian	Malaitan	159.95	-9.02	Choin_et_al	PASS
B00I2B6	Malaita	Malaita	NA	Malaita	Solomon_Arch	Oceania	Austronesian	Malaitan	159.95	-9.02	Choin_et_al	PASS
B00I2B7	Malaita	Malaita	NA	Malaita	Solomon_Arch	Oceania	Austronesian	Malaitan	159.95	-9.02	Choin_et_al	PASS
B00I2B9	Malaita	Malaita	NA	Malaita	Solomon_Arch	Oceania	Austronesian	Malaitan	159.95	-9.02	Choin_et_al	PASS
B00I2BA	Malaita	Malaita	NA	Malaita	Solomon_Arch	Oceania	Austronesian	Malaitan	159.95	-9.02	Choin_et_al	PASS
B00I2BB	Malaita	Malaita	NA	Malaita	Solomon_Arch	Oceania	Austronesian	Malaitan	159.95	-9.02	Choin_et_al	PASS
B00I2BC	Malaita	Malaita	NA	Malaita	Solomon_Arch	Oceania	Austronesian	Malaitan	159.95	-9.02	Choin_et_al	PASS
B00I2BD	Malaita	Malaita	NA	Malaita	Solomon_Arch	Oceania	Austronesian	Malaitan	159.95	-9.02	Choin_et_al	PASS
B00I2BE	Malaita	Malaita	NA	Malaita	Solomon_Arch	Oceania	Austronesian	Malaitan	159.95	-9.02	Choin_et_al	PASS
B00I2BF	Malaita	Malaita	NA	Malaita	Solomon_Arch	Oceania	Austronesian	Malaitan	159.95	-9.02	Choin_et_al	PASS
B00I2BG	Malaita	Malaita	NA	Malaita	Solomon_Arch	Oceania	Austronesian	Malaitan	159.95	-9.02	Choin_et_al	PASS
B00I2BH	Malaita	Malaita	NA	Malaita	Solomon_Arch	Oceania	Austronesian	Malaitan	159.95	-9.02	Choin_et_al	PASS
B00I2BJ	Malaita	Malaita	NA	Malaita	Solomon_Arch	Oceania	Austronesian	Malaitan	159.95	-9.02	Choin_et_al	PASS
B00I2BL	Malaita	Malaita	NA	Malaita	Solomon_Arch	Oceania	Austronesian	Malaitan	159.95	-9.02	Choin_et_al	PASS
B00I2AI	Santa_Cruz	Santa_Cruz	NA	Santa_Cruz	Solomon_Arch	Oceania	Austronesian	Reefs_Santa_Cruz	165.85	-10.76	Choin_et_al	PASS
B00I2AJ	Santa_Cruz	Santa_Cruz	NA	Santa_Cruz	Solomon_Arch	Oceania	Austronesian	Reefs_Santa_Cruz	165.85	-10.76	Choin_et_al	PASS

B00I2AL	Santa_Cruz	Santa_Cruz	NA	Santa_Cruz	Solomon_Arch	Oceania	Austronesian	Reefs_Santa_Cruz	165.85	-10.76	Choin_et_al	PASS
B00I2AM	Santa_Cruz	Santa_Cruz	NA	Santa_Cruz	Solomon_Arch	Oceania	Austronesian	Reefs_Santa_Cruz	165.85	-10.76	Choin_et_al	PASS
B00I2AN	Santa_Cruz	Santa_Cruz	NA	Santa_Cruz	Solomon_Arch	Oceania	Austronesian	Reefs_Santa_Cruz	165.85	-10.76	Choin_et_al	PASS
B00I2AO	Santa_Cruz	Santa_Cruz	NA	Santa_Cruz	Solomon_Arch	Oceania	Austronesian	Reefs_Santa_Cruz	165.85	-10.76	Choin_et_al	PASS
B00I2AP	Santa_Cruz	Santa_Cruz	NA	Santa_Cruz	Solomon_Arch	Oceania	Austronesian	Reefs_Santa_Cruz	165.85	-10.76	Choin_et_al	PASS
B00I2AQ	Santa_Cruz	Santa_Cruz	NA	Santa_Cruz	Solomon_Arch	Oceania	Austronesian	Reefs_Santa_Cruz	165.85	-10.76	Choin_et_al	PASS
B00I2AR	Santa_Cruz	Santa_Cruz	NA	Santa_Cruz	Solomon_Arch	Oceania	Austronesian	Reefs_Santa_Cruz	165.85	-10.76	Choin_et_al	PASS
B00I2AS	Santa_Cruz	Santa_Cruz	NA	Santa_Cruz	Solomon_Arch	Oceania	Austronesian	Reefs_Santa_Cruz	165.85	-10.76	Choin_et_al	PASS
B00I2AU	Santa_Cruz	Santa_Cruz	NA	Santa_Cruz	Solomon_Arch	Oceania	Austronesian	Reefs_Santa_Cruz	165.85	-10.76	Choin_et_al	PASS
B00I2AV	Santa_Cruz	Santa_Cruz	NA	Santa_Cruz	Solomon_Arch	Oceania	Austronesian	Reefs_Santa_Cruz	165.85	-10.76	Choin_et_al	PASS
B00I2AX	Santa_Cruz	Santa_Cruz	NA	Santa_Cruz	Solomon_Arch	Oceania	Austronesian	Reefs_Santa_Cruz	165.85	-10.76	Choin_et_al	PASS
B00I2AY	Santa_Cruz	Santa_Cruz	NA	Santa_Cruz	Solomon_Arch	Oceania	Austronesian	Reefs_Santa_Cruz	165.85	-10.76	Choin_et_al	PASS
B00FLID	Bellona	Bellona_Rennell	NA	Bellona	Solomon_Arch	Oceania	Austronesian	Rennell_Bellona	159.79	-11.3	Choin_et_al	PASS
B00FLIH	Bellona	Bellona_Rennell	NA	Bellona	Solomon_Arch	Oceania	Austronesian	Rennell_Bellona	159.79	-11.3	Choin_et_al	PASS
B00FLS1	Bellona	Bellona_Rennell	NA	Bellona	Solomon_Arch	Oceania	Austronesian	Rennell_Bellona	159.79	-11.3	Choin_et_al	PASS
B00FLS5	Bellona	Bellona_Rennell	NA	Bellona	Solomon_Arch	Oceania	Austronesian	Rennell_Bellona	159.79	-11.3	Choin_et_al	PASS
B00FLIL	Bellona	Bellona_Rennell	NA	Bellona	Solomon_Arch	Oceania	Austronesian	Rennell_Bellona	159.79	-11.3	Choin_et_al	PASS
B00FLIP	Bellona	Bellona_Rennell	NA	Bellona	Solomon_Arch	Oceania	Austronesian	Rennell_Bellona	159.79	-11.3	Choin_et_al	PASS
B00FLI1	Bellona	Bellona_Rennell	NA	Bellona	Solomon_Arch	Oceania	Austronesian	Rennell_Bellona	159.79	-11.3	Choin_et_al	PASS
B00FLI5	Bellona	Bellona_Rennell	NA	Bellona	Solomon_Arch	Oceania	Austronesian	Rennell_Bellona	159.79	-11.3	Choin_et_al	PASS
B00FLI9	Bellona	Bellona_Rennell	NA	Bellona	Solomon_Arch	Oceania	Austronesian	Rennell_Bellona	159.79	-11.3	Choin_et_al	PASS
B00FLIT	Bellona	Bellona_Rennell	NA	Bellona	Solomon_Arch	Oceania	Austronesian	Rennell_Bellona	159.79	-11.3	Choin_et_al	PASS
B00FLS9	Bellona	Bellona_Rennell	NA	Bellona	Solomon_Arch	Oceania	Austronesian	Rennell_Bellona	159.79	-11.3	Choin_et_al	PASS
B00FLHX	Bellona	Bellona_Rennell	NA	Bellona	Solomon_Arch	Oceania	Austronesian	Rennell_Bellona	159.79	-11.3	Choin_et_al	PASS
B00FLIX	Bellona	Bellona_Rennell	NA	Bellona	Solomon_Arch	Oceania	Austronesian	Rennell_Bellona	159.79	-11.3	Choin_et_al	PASS
B00FLJ1	Bellona	Bellona_Rennell	NA	Bellona	Solomon_Arch	Oceania	Austronesian	Rennell_Bellona	159.79	-11.3	Choin_et_al	PASS
B00FLJ5	Bellona	Bellona_Rennell	NA	Bellona	Solomon_Arch	Oceania	Austronesian	Rennell_Bellona	159.79	-11.3	Choin_et_al	PASS
B00FLHH	Rennell	Bellona_Rennell	NA	Rennell	Solomon_Arch	Oceania	Austronesian	Rennell_Bellona	160.29	-11.64	Choin_et_al	PASS
B00FLHP	Rennell	Bellona_Rennell	NA	Rennell	Solomon_Arch	Oceania	Austronesian	Rennell_Bellona	160.29	-11.64	Choin_et_al	PASS
B00FLHD	Rennell	Bellona_Rennell	NA	Rennell	Solomon_Arch	Oceania	Austronesian	Rennell_Bellona	160.29	-11.64	Choin_et_al	PASS
B00FLHL	Rennell	Bellona_Rennell	NA	Rennell	Solomon_Arch	Oceania	Austronesian	Rennell_Bellona	160.29	-11.64	Choin_et_al	PASS
B00FLG9	Tikopia	Tikopia	NA	Tikopia	Solomon_Arch	Oceania	Austronesian	Tikopia	168.3	-12.29	Choin_et_al	PASS
B00FLGL	Tikopia	Tikopia	NA	Tikopia	Solomon_Arch	Oceania	Austronesian	Tikopia	168.3	-12.29	Choin_et_al	PASS
B00FLGT	Tikopia	Tikopia	NA	Tikopia	Solomon_Arch	Oceania	Austronesian	Tikopia	168.3	-12.29	Choin_et_al	PASS
B00FLH5	Tikopia	Tikopia	NA	Tikopia	Solomon_Arch	Oceania	Austronesian	Tikopia	168.3	-12.29	Choin_et_al	PASS
B00FLGD	Tikopia	Tikopia	NA	Tikopia	Solomon_Arch	Oceania	Austronesian	Tikopia	168.3	-12.29	Choin_et_al	PASS
B00FLGH	Tikopia	Tikopia	NA	Tikopia	Solomon_Arch	Oceania	Austronesian	Tikopia	168.3	-12.29	Choin_et_al	PASS
B00FLGP	Tikopia	Tikopia	NA	Tikopia	Solomon_Arch	Oceania	Austronesian	Tikopia	168.3	-12.29	Choin_et_al	PASS
B00FLGX	Tikopia	Tikopia	NA	Tikopia	Solomon_Arch	Oceania	Austronesian	Tikopia	168.3	-12.29	Choin_et_al	PASS
B00FLH9	Tikopia	Tikopia	NA	Tikopia	Solomon_Arch	Oceania	Austronesian	Tikopia	168.3	-12.29	Choin_et_al	PASS
B00FLH1	Tikopia	Tikopia	NA	Tikopia	Solomon_Arch	Oceania	Austronesian	Tikopia	168.3	-12.29	Choin_et_al	PASS

Table S2. Austronesian- and Papuan-related ancestry proportions for the Massim groups, estimation by ADMIXTURE, GLOBETROTTER, and RFMix.

		ADMIXTURE		GLOBETROTTER		RFMix		
Massim region	Group	Austronesian	Papuan	Austronesian	Papuan	Austronesian	Papuan	Uncertain
Collingwood Bay	Northern	0.3	0.7	0.28	0.72	0.23	0.67	0.1
	Wanigela	0.33	0.68	0.32	0.68	0.25	0.65	0.1
	Airara	0.3	0.7	0.28	0.72	0.23	0.67	0.09
Western Massim	Mainland eastern tip	0.38	0.62	0.33	0.67	0.27	0.61	0.11
	Normanby	0.37	0.63	0.33	0.67	0.27	0.61	0.11
	Fergusson	0.36	0.64	0.34	0.66	0.26	0.63	0.11
Northern Massim	Trobriand	0.52	0.48	0.42	0.58	0.43	0.45	0.12
	Gawa	0.52	0.48	0.42	0.58	0.43	0.45	0.12
	Woodlark	0.51	0.49	0.42	0.58	0.42	0.46	0.12
	Laughlan	0.52	0.49	0.41	0.59	0.41	0.46	0.13
Southern Massim	Misima	0.43	0.57	0.37	0.63	0.31	0.57	0.12
	Western Calvados	0.42	0.58	0.38	0.62	0.32	0.56	0.12
	Eastern Calvados	0.4	0.6	0.35	0.65	0.26	0.63	0.11
	Sudest	0.27	0.73	0.31	0.69	0.17	0.73	0.1
	Rossel	0.2	0.8	0.27	0.73	0.13	0.8	0.08
